# Investigating the Flexibility of H-ZSM-5 Zeolite Upon Adsorption of Coke Precursors: A Theoretical and Experimental Approach

**DOI:** 10.1021/acs.jpcc.4c07349

**Published:** 2025-01-02

**Authors:** Agnieszka Seremak, Izar Capel Berdiell, Bjørnar Arstad, Torstein Fjermestad, Stian Svelle

**Affiliations:** † Center for Materials Science and Nanotechnology (SMN), Department of Chemistry, University of Oslo, P.O. Box 1033, Blindern, Oslo N-0315, Norway; ‡ SINTEF Industry, Forskningveien 1, Oslo 0314, Norway

## Abstract

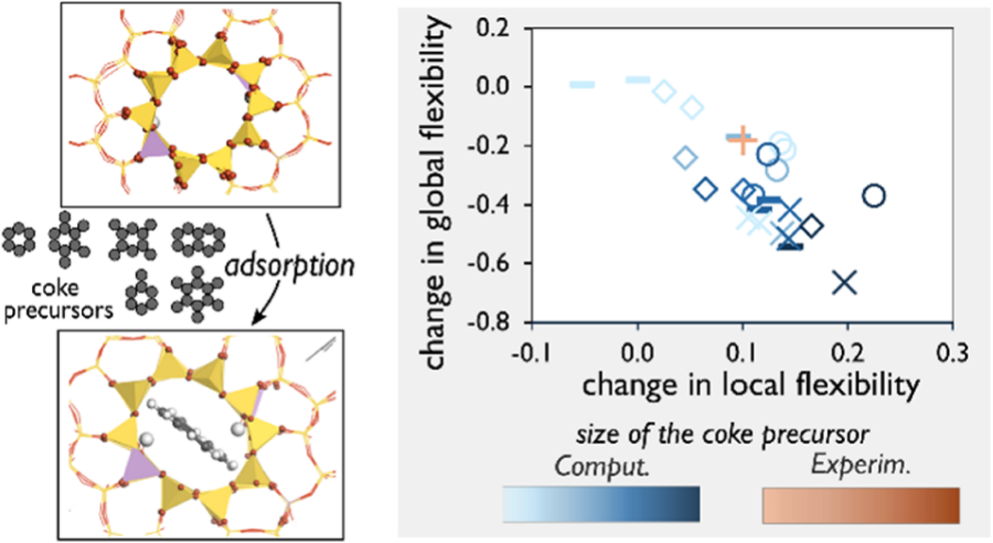

The flexibility of the H-ZSM-5 zeolite upon adsorption of selected coke precursors was investigated using both theoretical and experimental approaches. Four structural models with varying active site locations were analyzed through density functional theory (DFT) simulations to determine their responses to different types and quantities of aromatic molecules. Complementary experimental analysis was performed, allowing for a direct comparison with the theoretical findings, using thermogravimetric analysis (TGA), nitrogen adsorption (N_2_ adsorption), solid-state NMR, and X-ray diffraction (XRD). By employing proposed flexibility descriptors, significant structural changes in the MFI-type zeolite framework were identified, particularly in the unit cell parameters and the morphology of the straight channels. These changes were driven by electrostatic repulsion between adsorbates and by electrostatic attraction between adsorbates and the zeolite framework. The observed structural changes depended on both the active site location and the size and number of coke precursors. Consistent trends in structural flexibility were observed in both experimental and theoretical studies, primarily driven by variations in organic species loading. Our findings show the critical importance of active site location in influencing the magnitude of framework flexibility, which, in turn, affects the stabilization and accommodation of different coke precursors within the zeolite structure.

## Introduction

Zeolites are a prominent class of microporous materials commonly used as heterogeneous catalysts due to their various advantageous characteristics, including shape selectivity.^1,2^ There are hundreds of zeolite frameworks, each defined by the unique three-dimensional (3D) arrangement of their aluminosilicate tetrahedral building blocks. Among these, zeolite H-ZSM-5 has been extensively studied and industrially applied, particularly in the methanol-to-hydrocarbons (MTH) reaction. Topologically, it is an MFI-type zeolite, characterized by a three-dimensional system of cross-linked pores, formed by sinusoidal and straight channels, along *a*and *b*-directions, respectively, with pore diameters of around 5.5 Å.^3^ Its orthorhombic crystal structure is formed by 12 distinct T-sites, and each unit cell (uc) contains 192 oxygen atoms and 96 tetrahedrally coordinated T atoms. These T atoms can either be silicon or be substituted by aluminum. The incorporation of aluminum into the framework results in an excess negative charge, which is compensated by adding a proton, thereby creating a Brønsted acid site (BAS) on one of the adjacent oxygen atoms. The combination of Brønsted acidity and large specific surface area (ranging between 350 and 450 m^2^/g)^4^ provides an ideal environment for the adsorption of methanol and its subsequent conversion to desired products.^5^ However, Brønsted acidity is also the main cause of zeolite catalyst deactivation,^6,7^ and the inherent high porosity of the material enables the accumulation of nonreactive carbonaceous species known as coke. These polyaromatic and methylated aromatic molecules build up into graphene-like entities, blocking access to the BAS and eventually causing a complete loss of catalytic activity. Numerous studies have focused on understanding the formation mechanism of these aromatic ring-based molecules.^8−11^ It is generally accepted that coke deactivation begins at the BAS and occurs both on the external surface and within the pores of the zeolite.^12^

Parallel investigations by various research groups have examined the effect of internal coke species on the structural changes of MFI-type zeolites.^13−16^ One of the earliest investigations into ZSM-5 guest-induced flexibility was conducted by Mentzen and Lefebvre, using silicalite-1, a zeolite with no acid sites, as a host structure and benzene as guest molecules. They applied X-ray diffraction (XRD) with Rietveld refinement and simple energy calculations to observe that at high loading of benzene, the zeolite’s straight channels deform from circular into an elliptical shape. The unit cell parameters also change to a tetragonal-like crystal system.^13^ Around the same time, Weitkamp and co-workers used a similar approach to study acidic H-ZSM-5 and its interaction with naphthalene. Their results showed structural distortion of the zeolite catalyst upon naphthalene sorption.^14^ More recently, Wei’s group captured similar interactions using in situ electron microscopy, with high-quality images clearly demonstrating the flexibility of zeolite ZSM-5 upon benzene sorption.^15^ These investigations have revealed that the MFI-type zeolite should not be perceived as a rigid structure but rather as displaying flexibility in response to interactions with guest species. An additional study using *operando* XRD investigated the influence of coke molecules on the MFI framework, showing that the presence of nonreactive carbonaceous species deforms the catalyst architecture. This deformation affects the shape of the straight channels, which can be referred to as a local distortion, and causes changes in the *a* and *b* unit cell parameters, affecting the material’s structure globally.^16^ These experimental results suggested a correlation between the local and global structural distortions. The potential to utilize structural deformation of the H-ZSM-5 framework as a measure of activity loss has thus become apparent.

In a related study conducted by Brogaard et al.,^17^ a theoretical investigation using periodic density functional theory (DFT) was conducted to examine the adsorption energies of different arenes (as coke precursors) in H-ZSM-5. Multiple models with distinct BAS locations were examined to gain insights into the effect of these molecules on zeolite catalyst deactivation. The study also provided valuable information regarding the preferred adsorption sites. Each molecule occupied the intersection between the straight and sinusoidal channels.

**Figure 1:**
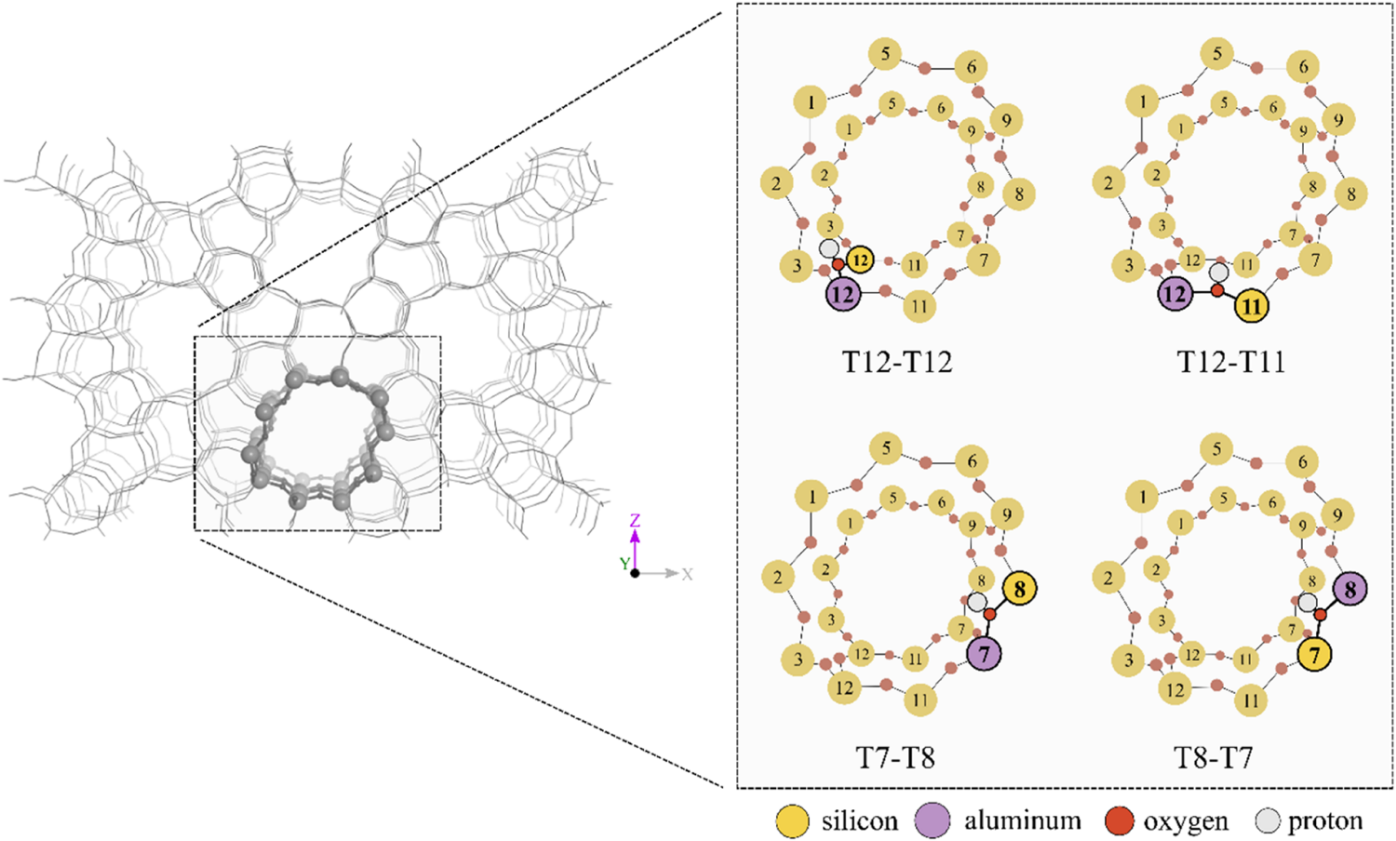
Graphical representations of the active site models of zeolite H-ZSM-5. The active sites are labeled as T*X*–T*Y*, where *X* indicates the location of aluminum (Al) and *Y* indicates silicon (Si). The active site (BAS) is positioned between them.

This work aims to build on these findings by investigating the coke-induced flexibility of MFI-type zeolites through a combined theoretical and experimental approach. Focus is placed on the impact of sorption of various coke precursors on the response of the zeolite framework, emphasizing the number of these molecules and their influence on the framework’s flexibility. Additionally, the aim is to understand the potential impact of the active site location and aluminum distribution. Given that exploring all possible aluminum site locations and corresponding Brønsted acid sites is computationally intense and may lead to combinatorial explosion issues, our study adopts a focused approach. We examine two models with identical aluminum siting but different active site locations and two models with the same active site location but varying aluminum siting (Figure 1). Although it is important to highlight that the BAS location is not restricted to one specific position and can jump from one oxygen to another, a phenomenon known as proton hopping,^18,19^ this study does not consider potential proton mobility.

The objective of this work is to characterize guest-induced distortions in the framework and elucidate the relationship between changes in the local environment, identified with pore shape, and the global structure, defined by the unit cell lattice. For this purpose, local and global flexibility descriptors have been defined. Furthermore, identifying the most stable H-ZSM-5 model for interacting with coke precursors from the studied pool could provide insight into designing a zeolite catalyst with better performance and greater resistance to coke formation. Finally, to test theoretical trends, adsorption experiments using different organic compounds as adsorbates were performed, providing valuable insight into the flexibility of zeolite under experimental conditions.

## Methods

### Computational Section

As a catalyst model, in full, we used a 1 × 1 × 1 unit cell of the H-ZSM-5 structure with the unit cell parameters taken from IZA-SC, i.e., *a* = 20.09 Å, *b* = 19.738 Å, *c* = 13.142 Å.^20^ To transform a siliceous zeolite into a solid acid, a Si atom was substituted by a trivalent Al atom with a proton on one of the adjacent oxygen atoms to compensate for the excess negative charge.

All periodic DFT calculations were performed with the CP2K software package version 9.2 and the Gaussian and plane wave methods.^21^ The Gaussian-type pseudopotentials Goedecker–Teter–Hutter (GTH) with PBE exchange–correlation functional and TZVP MOLOPT basis set were implemented.^22,23^ Grimme D3 dispersion corrections were used for all calculations.^24^ To ensure convergence of the studied system, a plane wave cutoff was set to 800 Ry, and a relative plane wave cutoff, which determines the grid at which a Gaussian is mapped, was set to 60 Ry. (Supporting Information 1)

#### Model Construction

The MFI zeolite structure model investigated in this study has a Si/Al ratio of 31, which corresponds to 3 aluminums and 3 protons per unit cell. The aluminum atoms were distributed between two straight channels, with two atoms placed in one channel at a distance greater than 13 Å apart, measured from the center of the atoms, and the remaining atom positioned in the second channel. Images depicting the entire unit cell with the distribution of Al and protons are provided in Supporting Information 3, Figure S4. All four oxygen atoms adjacent to aluminum were examined for potential proton locations, and the focus was placed on determining the location with the lowest electronic energy. Several aluminum (and active site) locations were considered: T7 (with a proton between T7–T8), T8 (proton between T8–T7), and T12 (proton between T12–T12 and T12–T11), as depicted in Figure 1. These particular sites were chosen due to their high stability (in the case of T7),^25^ the accessibility of the pores to the guest species (in the case of T12), and to compare the influence of the active site when aluminum is in the same T site (T12–T12 vs T12–T11). Furthermore, the investigation aims to assess the potential for different responses of the framework when the proton is situated between the same T-sites (T7–T8 vs T8–T7), based on the location of aluminum. Calculations for a siliceous system were also conducted, demonstrating how a zeolite without acid sites responded to various guest species (Supporting Information 11).

**Figure 2:**
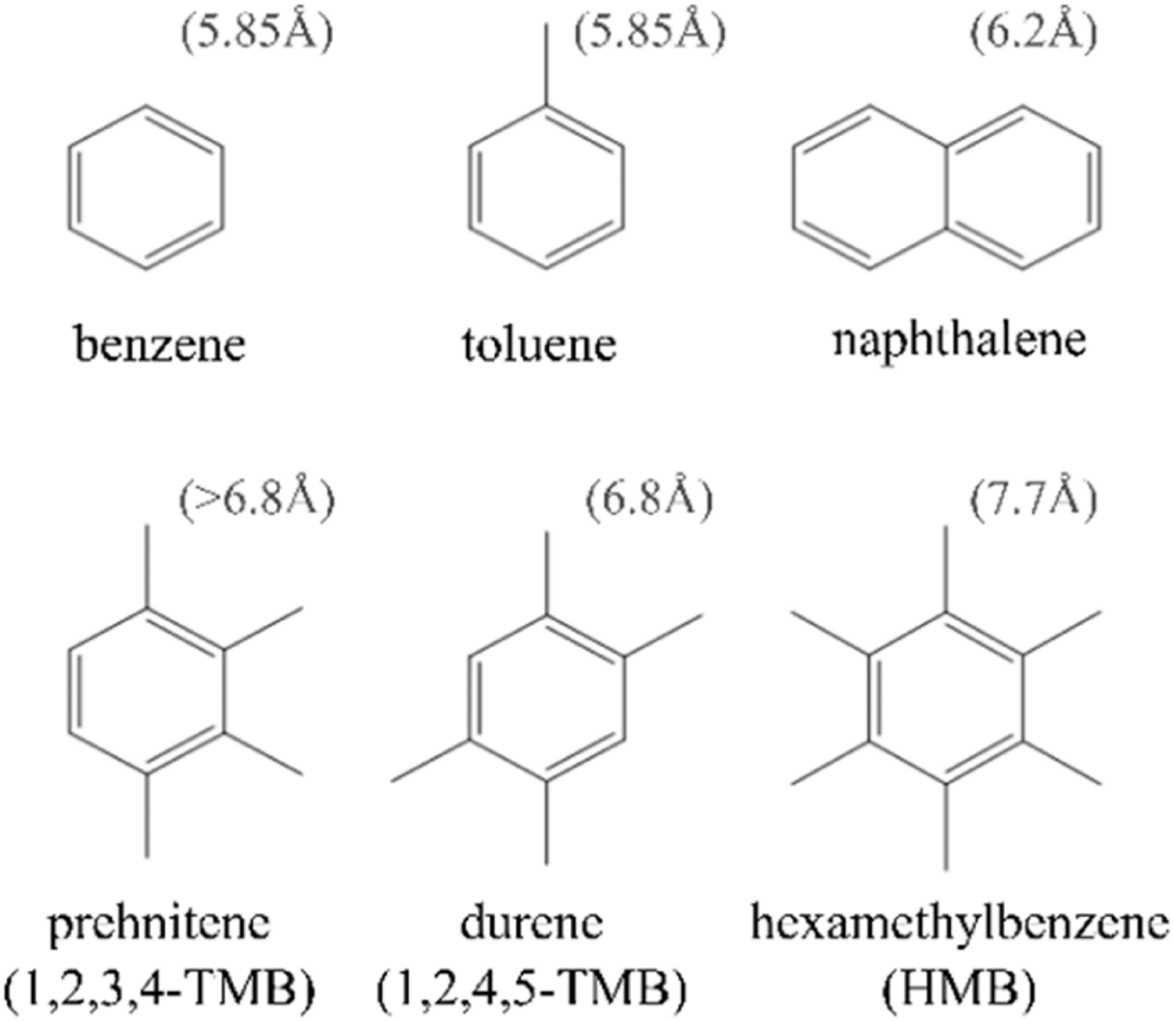
Molecules studied as coke precursors. The kinetic diameter value for each molecule is provided in brackets above and is taken from refs (27−29).

Hydrocarbons were placed in either the intersection, straight, or sinusoidal channel in a stepwise manner, up to 4 molecules per unit cell (uc), ensuring the first three molecules were always in proximity to the active site. The first and second molecules were added along the same straight channel, while the third and fourth molecules were positioned inside the other straight channel. This process is visually explained in Supporting Information 3, Figure S5. This configuration could potentially influence the extent of flexibility following the addition of the second coke precursor. However, examining the preferred site after each successive molecule’s addition or for each zeolite model would result in a combinatorial problem, making it challenging to compare the models. Therefore, the same order of adding molecules into the zeolite’s pore was consistently used. The molecules representing coke precursor were selected based on the identification of coke species, as reported in the work of Rojo-Gama et al.,^26^ i.e., benzene, toluene, naphthalene, prehnitene (1,2,3,4-TMB), durene (1,2,4,5-TMB), and hexamethylbenzene (HMB) (Figure 2).

Geometry optimization was carried out for empty models and models with guest molecules placed at various locations and orientations. The Broyden–Fletcher–Goldfarb–Shanno (BFGS) geometry optimization algorithm^30−33^ was utilized to identify the local minima of the potential energy surface (PES). Although this study cannot claim to have found the global minimum energy for each model, a few steps have been taken to ensure the reliability of the presented results. For each guest molecule, the same geometry optimization algorithms were applied while the positions and orientations were varied within each active site model. This approach allows for the accounting for different possible configurations and their effects on electronic energies, ultimately enabling the identification of the most stable model. Then, a cell optimization protocol was carried out, enabling unit cell relaxation while preserving fixed angles at 90°. This final calculation yields information on unit cell parameters and electronic energies and provides the optimized structures of the zeolite with distinct coke precursors loaded from one up to four molecules/uc.

#### Quantifying Flexibility Descriptors

To characterize the flexibility of the zeolite catalyst, this study focuses on quantifying changes in atomic coordinates, lattice parameters, and pore shape. Correlating these quantified changes to the number and type of coke precursors could yield an accurate descriptor for zeolite deactivation.

The first primary method employed in this study is the calculation of the root-mean-square deviation (RMSD) of local atomic environments. The RMSD provides a comprehensive measure of the overall structural differences by comparing the local environment for each atom of the zeolite in its empty form and when loaded with varying numbers of coke precursor molecules. A more complete picture of the local structural environment and even subtle differences in local geometries between the studied systems can be captured by using the atomic environment instead of raw atomic coordinates. This calculation was performed using the atomic simulation environment (ASE)^34^ Python library. For each atom *i*, the local environments around that atom ***E***_*i*_^(empty)^ and ***E***_*i*_^(loaded)^ were defined. The local RMSD for atom *i* is defined by eq 1.
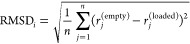
1

where *r*_*j*_^(empty)^ and *r*_*j*_^(loaded)^ are the coordinates of the *j*th atom in the local environment of empty and loaded structures, respectively. Here, *n* is the number of atoms in the largest local environment with a cutoff distance of 5 Å applied.

Subsequently, the average RMSD for the entire structure is calculated by using eq 2.
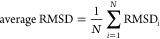
2

where *N* is the total number of atoms in the structure.

From the obtained Average RMSD values for each structure, we also determined the normalized root-mean-square deviation (NRMSD) through eq 3:
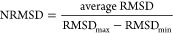
3

Normalized root-mean-square deviation (NRMSD), calculated by dividing the average RMSD by the difference between the maximum (RMSD_max_) and minimum (RMSD_min_) average RMSD values among all studied structures (Supporting Information 6).

In addition to the RMSD, another descriptor previously introduced for the H-ZSM-5 catalyst focuses on changes in the lattice parameters when the material is filled with different coke species. Specifically, it considers the difference in length between the **a** and **b** unit cell vectors (referred to as *a* minus *b* or simply “*a*–*b*”).^26^ This parameter was adopted in the current work to identify global structural changes in the studied material, serving as an indication of flexibility. The choice of (*a*–*b*) is justified by the significant changes in **a** and **b** unit cell vectors upon coke loading, making it a more sensitive descriptor than either (*a*–*c*) or (*b*–*c*). For a detailed discussion on the selection of this specific descriptor, see the Supporting Information from our previous paper.^35^

To quantitatively assess the pore flexibility and the local distortion, we adopted a method from Fei Wei’s group to examine the shape of the 10-membered ring within the straight channel.^15^ By measuring the distance between the centers of two opposite T atoms, identifying the longest and shortest diameter, and calculating the ratio between them (*D*_max_/*D*_min_), we characterized the pore’s ovality and, hence, the local flexibility. These measurements were taken for each of the pore openings, and the average value was used.

### Experimental Section

Adsorption experiments were performed with a commercial H-ZSM-5 zeolite, CBV-8014 (Zeolyst International), with a Si/Al ratio equal to 40. Heated overnight quartz ampules (1 cm ID, 50 cm length, 80 °C) were loaded with 150 mg of pressed and sieved to 250–425 μm fraction catalyst that had also been dried at 300 °C overnight. Several organic molecules in a nominal loading of 4 molecules per zeolite unit cell were added into the ampule, which was flame-sealed under vacuum using a liquid nitrogen dewar in the bottom of the ampule to avoid organic evaporation. Benzene and toluene were initially considered for the experiments, but their high volatility and low boiling points (benzene: 80.1 °C, toluene: 110.6 °C) presented impractical challenges in maintaining their presence within the ampule. An attempt with toluene resulted in its complete removal under a vacuum. Consequently, naphthalene, tetramethylbenzene isomers (prehnitene and durene), and hexamethylbenzene were selected for the adsorption experiments. The sealed ampules were equilibrated at 200 °C for different durations: 50 h for naphthalene, 100 h for tetramethylbenzenes, and 180 h for hexamethylbenzene. All samples were stored inside a glovebox prior to characterization with strict measures to prevent water contamination.

#### Catalyst Characterization

The number of organic species adsorbed in the catalysts was quantified by thermogravimetric analysis (TGA) on a Netzsch STA 449 TG. Approximately 18 mg of catalyst was heated under 25 mL/min of synthetic air to 800 °C at a rate of 10 °C/min. Adsorption properties of the hydrocarbon loaded materials were evaluated by nitrogen adsorption–desorption measurements performed at the nitrogen boiling point (−196 °C) on a Bel Belsorp-mini II instrument. Before the measurements, the samples loaded in the cells inside the glovebox were pretreated under vacuum for 3 h at 35 °C. Specific surface area values were calculated using the BET equation.^36^ Micropore and mesopore volumes were estimated using the *t*-plot and BJH methods, respectively. For structural analysis, empty and loaded zeolites were analyzed by XRD using a Bruker D8-A25 in transmission capillary geometry with a Ge(111) Johanssen monochromator and Lynxeye detector with Cu K-α-1 radiation (λ = 1.5406 Å). Samples were placed in 0.7 mm glass capillaries inside a glovebox, and the capillaries were flame-sealed. X-ray diffractograms were recorded at 25 °C.

Data analysis by the Rietveld method was carried out in TOPAS V6.^37^ The model for the ZSM-5 structure with organic guest species was adapted from our previous work and includes 7 dummy atom sites corresponding to the highest electron density peaks in difference Fourier maps ZSM-5 samples.^38^ The 2θ range fitted was 6 to 70°. The same model of ZSM-5 was used for all refinements: (zero error, lattice parameters, scale factor, Gaussian size broadening, strain broadening, and occupancy of 7 dummy carbon atoms). Isotropic thermal parameters of oxygen and silicon are assumed to be equal for all Si and all O to reduce the number of parameters for each refinement. Carbon (dummy atom) thermal parameters were fixed to 10. The nonframework species mass (NFSM) and (*a*–*b*) and unit cell volume (UCV) parameters were calculated in the input file for each powder pattern. The use of angle and distance restraints to the tetrahedral building units allowed atomic position refinement of the framework and to measure the length between the centers of two opposite T atoms and calculate the aspect ratio between the longest and shortest distances (*D*_max_/*D*_min_).

Solid-state NMR was carried out using a Bruker AVANCE Neo instrument operating at a field of 18.7 T (1H resonance frequency, 800 MHz). ^1^H–^13^C cross-polarization (CP) experiments were conducted for the ^13^C NMR spectra. 10000 scans were accumulated for each spectrum, except for one sample (pure 1,2,4,5-TMB), which was acquired with only 200 scans. For the experiments, we applied a recycle delay of 5 s and a shaped ramp contact pulse for 2 ms. All experiments were carried out using a double-channel H-X 3.2 MAS probe at 298 K. The MAS rates were either 15 or 20 kHz. A background from carbon was removed from all spectra. Before Fourier transformation, zero filling and apodization were done to improve the spectra. Apodization was performed by multiplying the accumulated FIDs with a decaying exponential function with a broadening factor of 200 Hz.

## Results and Discussion

### Computational Section

#### Empty H-ZSM-5

**Figure 3:**
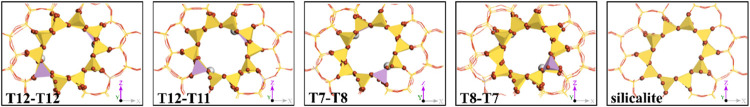
Graphical representation of the snapshots of optimized empty zeolite MFI models zoomed in on the straight pore channel.

The simulation cells of the empty H-ZSM-5 models were optimized. The resulting unit cell parameters for empty zeolite models with various active site distributions are presented in Table 1, and detailed snapshots of the straight pores are shown in Figure 3. Analysis indicates that the global flexibility descriptor (*a*–*b*) ranged from 0.25 to 0.3 when the active site was within the same 10-member ring (T12–T11, T7–T7, and T8–T7). For the T12–T12 configuration, however, an elongation along the sinusoidal channel direction was observed, indicated by an increased (*a*–*b*) parameter, similar to that in the T7–T7 case (Supporting Information 2). Small variations in the local flexibility descriptor (*D*_max_/*D*_min_) were also observed depending on the active site locations; however, no clear correlation was evident between the global and local flexibility descriptors for the empty zeolite. These results emphasize the significant influence of BAS positioning on the structural characteristics of zeolite H-ZSM-5, showing how even subtle changes in the active site distribution can affect its framework.

**Table 1:** Empty H-ZSM-5 Structural Parameters: Values Are Reported in Å

		active site location				
		T12–T12	T12–T11	T7–T8	T8–T7	silicalite
unit cell parameters	*a*	20.29	20.15	20.14	20.16	20.24
	*b*	19.86	19.85	19.88	19.91	19.80
	*c*	13.46	13.45	13.44	13.41	13.38
	volume	5423	5376	5379	5382	5369
global descriptor	***a–b***	**0.43**	**0.30**	**0.27**	**0.25**	**0.44**
	*D*_max_	9.91	9.67	10.05	9.79	10.00
	*D*_min_	8.85	8.97	8.50	8.80	8.70
local descriptor	***D***_**max**_**/*****D***_**min**_	**1.12**	**1.08**	**1.18**	**1.11**	**1.15**

#### Filling the Pores with Coke Precursors

To accurately model and assess the flexibility of MFI-type zeolites when exposed to various guest species, an iterative process was applied to explore potential locations and orientations for each molecule within the H-ZSM-5 pores. This process ultimately identified the structures with the lowest electronic energy. Our findings revealed that each molecule preferentially occupies the more accessible intersections between the straight and sinusoidal channels. This observation aligns with previous research,^17^ as these intersections offer reduced confinement.

**Figure 4:**
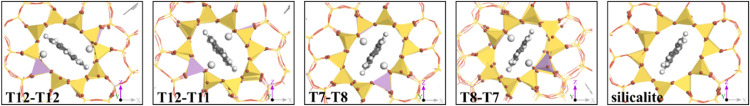
Graphical representation of the naphthalene molecule orientation inside the intersection between the straight and sinusoidal channels of H-ZSM-5 at a loading of 4 molecules per unit cell.

We then analyzed the differences in molecular orientation within these intersections based on the location of the active site. Figure 4 presents snapshots of a straight pore containing naphthalene at a loading of 4 molecules per unit cell, demonstrating variations in molecular orientation. These variations suggest that interactions between the proton and the π system of the organic species play a crucial role in determining the preferred orientation of the coke molecules within the zeolite channels. A comparison between the empty model (Figure 3) and the model loaded with naphthalene (Figure 4) reveals significant changes in the pore shape. In particular, for the zeolite structure with aluminum in the T12 site, the framework undergoes a major rearrangement of tetrahedral building blocks to accommodate the guest molecules. This adjustment results in the longest diameter of the 10-member ring becoming the shortest. The tilting of the pore is especially evident when compared to models with active sites between the T7 and T8 atoms. Alternatively, in the silicalite structure, the molecules align with the longest diameter of the straight channel. The differences between the empty silicalite model and the one loaded with coke precursor molecules are subtle and not easily distinguished through simple observation. Therefore, a more detailed quantitative analysis was carried out, with the results available in Supporting Information 11.

#### Electronic Energy of Studied Models

To evaluate the stability of each studied system, their electronic energy was assessed by calculating the difference between the electronic energies of a zeolite model containing N guest molecules and the empty zeolite model. The active site model with the highest energy for each molecule type and quantity was identified, indicating the least stable configuration. This specific configuration was assigned a reference energy of 0 kJ mol^–1^ with all other energies reported relative to it. This process was carried out for every type and number of loaded molecules, providing insights into identifying the optimal position of the BAS for accommodating coke precursors. The model with the lowest energy for each specific combination of molecule type and quantity is shown in bold in Table 2.

**Table 2:** Relative Electronic Energies (kJ mol^–1^) of Each Active Site Model Are Dependent on the Type and Number of Molecules

coke precursor	benzene	toluene	naphthalene	1,2,3,4-TMB	1,2,4,5-TMB	HMB
one molecule/uc						
T12T12	–7	0	0	–6	–15	–34
T12T11	–**42**	–5	–2	0	–**67**	–**46**
T7T8	–8	–3	–4	–5	–1	–1
T8T7	0	–**6**	–**11**	–**8**	0	0
two molecules/uc						
T12T12	–7	0	0	0	0	–37
T12T11	–**50**	–**28**	–**63**	–**39**	–**65**	–**52**
T7T8	–2	–2	–17	–3	–14	–16
T8T7	0	–7	–33	–4	–23	0
three molecules/uc						
T12T12	–2	0	0	–9	–44	–44
T12T11	–**56**	–**37**	–**73**	–**56**	–**89**	–**52**
T7T8	–10	–6	–26	–2	–32	0
T8T7	0	–8	–30	0	0	–1
four molecules/uc						
T12T12	0	0	0	0	–39	–48
T12T11	–**57**	–**41**	–**104**	–**48**	–**79**	–**62**
T7T8	–10	–8	–52	–4	–45	–18
T8T7	–7	–10	–53	–20	0	0

Comparing the different models explored in our study, it is evident that the structural configuration of H-ZSM-5 with aluminum positioned at the T12 site and the Brønsted acid site located on the oxygen between the T12–T11 atoms consistently exhibits the lowest electronic energy when loaded with any amount of the studied coke precursor molecules with only minor exceptions. These exceptions may arise from local energy minima with characteristics that differ from those of the other models. Other active site distribution structures result in significantly higher energy values, indicating reduced favorability for guest molecule accommodation. These findings emphasize the critical role of the specific arrangement of active sites in influencing the energetic behavior and guest molecule accommodation preferences in the MFI-type zeolite.

#### Atomic Distortion Trends

**Figure 5:**
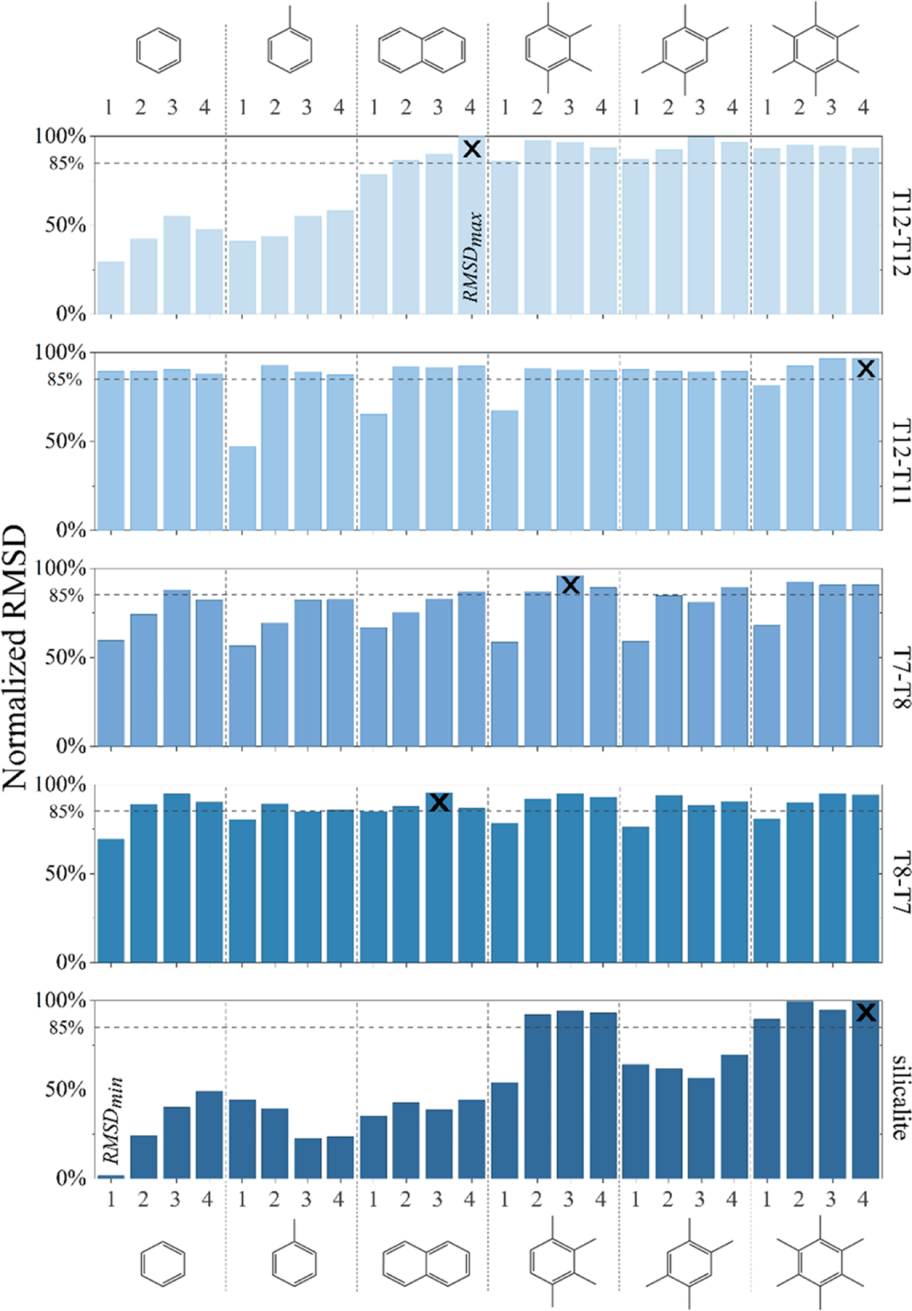
Normalized root-mean-square deviation (NRMSD) values for each analyzed structure, comparing the empty zeolite model to those loaded with various types and numbers of aromatic molecules. The *X*-axis marks the number and type of molecules adsorbed inside the zeolite, while the *Y*-axis reports the NRMSD as a percentage. The *X* indicates the most distorted structure for each active site model. RMSD_max_ and RMSD_min_ are the maximum and minimum average RMSD values chosen for the RMSD normalization.

To investigate the H-ZSM-5 framework flexibility, a normalized root-mean-square deviation (NRMSD) analysis was performed (eq 3). The results are listed in Figure 5.

For zeolite models with BAS located between the atoms of a 10-member ring of the straight channels (T12–T11, T7–T8, and T8–T7), the NRMSD exceeds 85% at a loading of at least two molecules per unit cell, regardless of the size of the analyzed coke precursors. In a model with BAS between two ten-membered rings (T12–T12), a similar NRMSD magnitude is observed only for molecules with a kinetic diameter ≥6.2 Å. In the absence of an active site, the distortion is much less pronounced; the NRMSD exceeds 85% only for molecules with a kinetic diameter of ≥6.8 Å. This indicates that the presence of an active site significantly contributes to the higher flexibility of the framework, evident even when loaded with the simplest aromatic molecule.

This analysis also enables investigation of the local RMSD*_i_* values of individual atoms within the structure (eq 1). The values of the local RMSD_*i*_ in the most distorted structure for each active site model were examined (indicated by *X* in Figure 5). The highest RMSD_*i*_ values, exceeding 3 Å, are recorded for the oxygen atoms in the 10-member ring of the straight channel (Supporting Information 5). This value does not indicate a physical movement of 3 Å but reflects the displacement relative to the local environment of a specific atom in the empty model, indicating significant local structural rearrangements.

These observations suggest that parts of the zeolite framework, particularly those near the guest molecules, are more susceptible to structural changes upon molecule adsorption. Analyzing the shape changes of the straight channel, i.e., the 10-member ring, can provide further insights into the zeolite flexibility. Furthermore, correlating these localized distortions with global structural changes, such as how the unit cell parameters respond to the adsorption of various coke precursors, can enhance our understanding of the structural effects of inner coke precursors on MFI-type zeolites.

#### Relationship between Local and Global Flexibility

**Figure 6:**
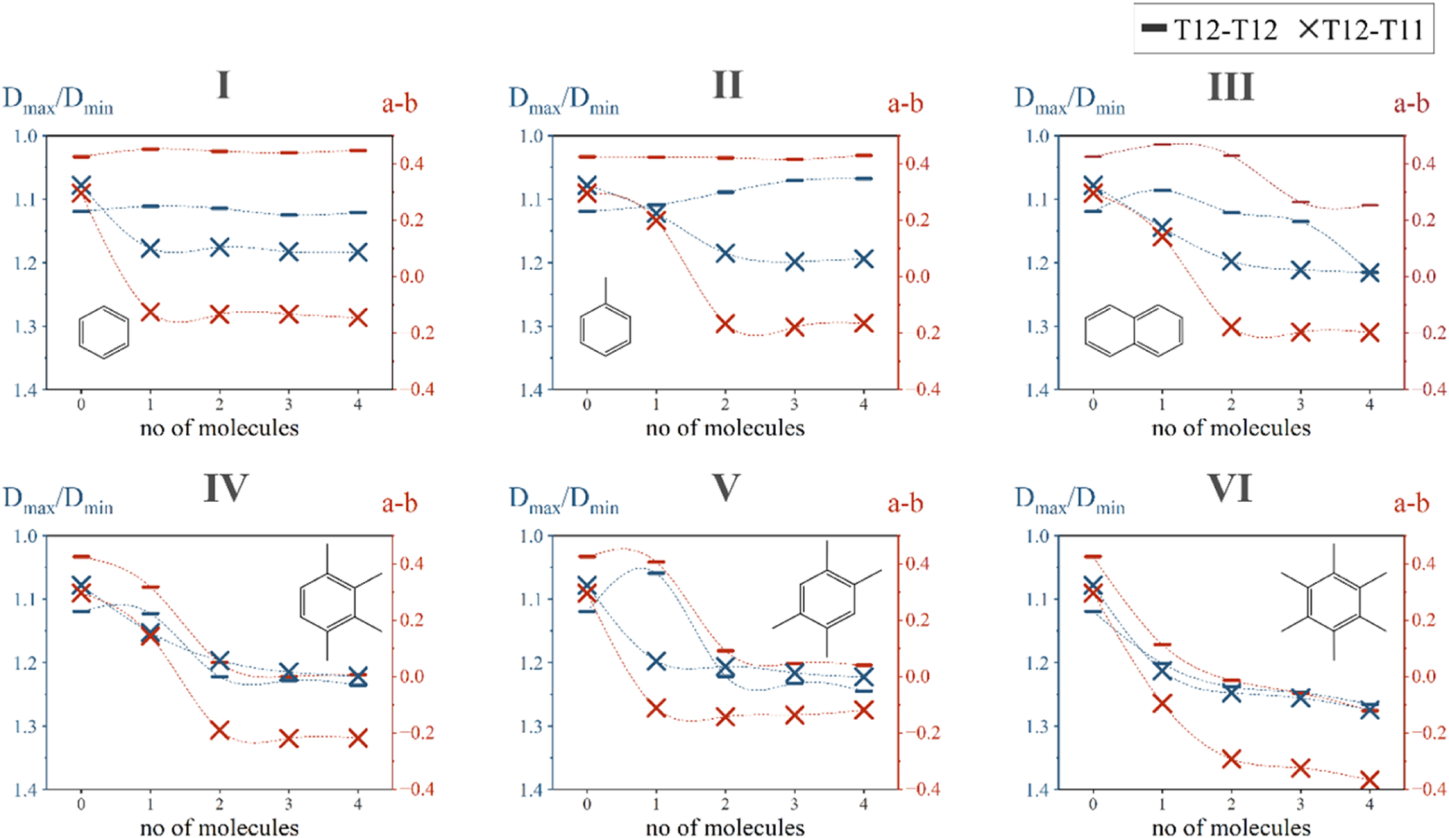
Relationship between local (*D*_max_/*D*_min_) and global (*a*–*b*) flexibility descriptors of models sharing the same Al location but with different BAS positions, dependent on the number and type of guest molecule (Roman numerals).

The changes in local and global flexibility descriptors in response to different types and amounts of adsorbed coke precursor molecules are illustrated in Figure 6. Notably, the axis for local distortion (*D*_max_/*D*_min_) is inverted, while the axis for the global descriptor (*a*–*b*) remains in its regular orientation. Regardless of the active site location and type of coke precursor, an inverse relationship between local and global structural parameters is observed. With increasing coke precursor loading, the local flexibility descriptor values increase while global flexibility descriptor values decrease. This indicates two simultaneous material responses: as the straight channel pore becomes more oval-shaped (higher *D*_max_/*D*_min_), the (*b*) unit cell parameter expands, and/or the (*a*) unit cell parameter contracts (explained further later). This trend is consistent with previous findings by Kalantzopoulos et al.^16^ A similar trend is found in our experimental results presented in Table 3 (see below).

**Table 3:** Data Collected from Refinement Results for Empty H-ZSM-5 and with Naphthalene, Prehnitene (1,2,3,4-TMB), Durene (1,2,4,5-TMB), and Hexamethylbenzene

	empty	naphthalene	1,2,3,4-TMB	1,2,4,5-TMB	HMB
*a* (Å)	20.088	19.962	20.068	20.070	20.094
*b* (Å)	19.915	19.970	19.932	19.938	19.918
*c* (Å)	13.395	13.381	13.392	13.394	13.398
volume (Å^3^)	5359	5334	5357	5360	5362
***a*–*b***	**0.173**	–**0.007**	**0.136**	**0.132**	**0.176**
***D***_**max**_**/*D***_**min**_	**1.06**	**1.16**	**1.08**	**1.09**	**1.06**
NFSM (%)	0.39	8.83	5.01	5.73	2.03

Our findings also indicate that the framework’s response is nonlinear with respect to the number of coke molecules per unit cell across all studied models, as presented in Figure 6. Our results indicate that as the loading increases, the structural changes in the zeolite framework initially increase rapidly; however, the rate of these changes slows and eventually reaches a point where no further significant structural changes occur at both local and global levels. These findings imply that the H-ZSM-5 zeolite catalyst’s structural flexibility is highly sensitive to coke loading, which could significantly influence its catalytic efficiency and durability.

Examining the differences between the studied active site models, we found that the zeolite model with an active site between T12 and T11 (crosses in Figure 6) shows structural changes even with small amounts of the simplest arene molecule (Figure 6I). This model was generally the most stable for accommodating various coke precursors, except for three outliers (Table 2). These outliers exhibit reduced distortion after the addition of the first molecule (Figure 6II–IV), indicating a direct relationship between geometric parameters and energy profiles. For these systems, the identified local minimum has a different structural characteristic compared to other structures with an active site between T12–T11. This is also reflected in the NRMSD data (Figure 5), where these three structures exhibited less pronounced structural distortions compared with other structures within this active site model. Conversely, when the active site is located between two 10-membered rings (T12–T12, minuses in Figure 6), the zeolite catalyst showed lower sensitivity to small organic compounds deforming only when loaded with aromatics of a kinetic diameter above 5.85 Å. The same observation was corroborated by the NRMSD data (Figure 5). These findings emphasize clear differences in zeolite behavior upon adsorption of various aromatic compounds and depending on the active site location.

Further analysis was done using models with the same active site location but different Al distributions, i.e., T7–T8 and T8–T7 (Supporting Information 7, Figure S6). The structural changes upon increasing the loading of various coke precursors between these models are very similar.

#### Detailed Unit Cell Parameter Analysis

**Figure 7:**
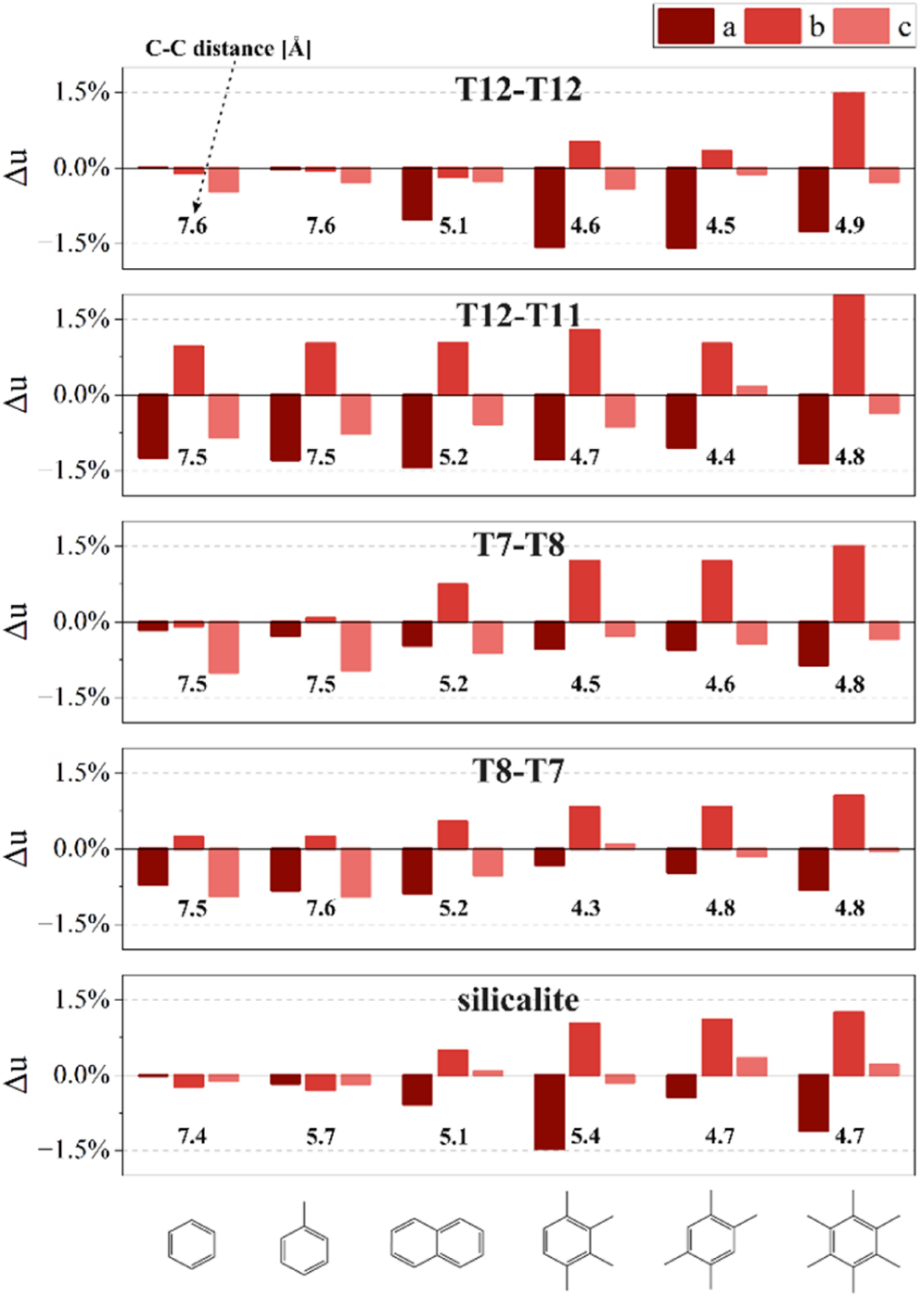
Relative changes (in %) in the cell parameter vector lengths between fully loaded and empty H-ZSM-5 structures.

To better understand how the unit cell is deforming, the analysis was expanded to include unit cell parameter data derived from a cell optimization protocol (Supporting Information 3). Changes in the **a**, **b**, and **c** unit cell vectors were computed by comparing unit cell parameters of zeolite loaded with four molecules per unit cell to those of the empty zeolite, as illustrated in Figure 7 (Supporting Information 8). For zeolites loaded with smaller molecules (e.g., benzene, toluene, and naphthalene), a significant contraction in the *a*-direction, lesser contraction in the *c*-direction, and minor expansion in the *b*-direction were observed. This indicates that the change in the (*a*–*b*) parameter upon loading is primarily influenced by the decrease in the **a** unit cell vector for these molecules. Conversely, when zeolites were loaded with larger methylated benzenes, more expansion in the *b*-direction than contraction in the a-direction was observed, with minor changes in *c*. In this case, the (*a*–*b*) change is predominantly governed by the expansion in the **b** unit cell vector.

Average intermolecular distances between carbon atoms of neighboring molecules were measured (as depicted in Figure S5) and reported in Figure 7 (indicated by the arrow). All molecules demonstrated intermolecular C–C distances greater than the van der Waals radii sum (3.4 Å), indicating an absence of Pauli repulsion.^39^ To better understand the interactions influencing these structural changes, single-point energy calculations with Hirshfeld charges were performed on the optimized T12–T11 model loaded with four benzene and four hexamethylbenzene molecules and on a silicalite model loaded with four hexamethylbenzene molecules (Supporting Information 9). For the zeolite model T12–T11 with benzene, each molecule had a net charge of about 0.33 (1.30 in total), whereas the zeolite framework had a net charge of −1.30. The intermolecular C–C distances greater than 7 Å suggest that the unit cell contraction in the a-direction can be attributed to electrostatic attractive forces between benzene molecules and the zeolite framework, particularly the zeolite oxygen atoms. For larger methylated benzenes, each hexamethylbenzene molecule had net charges of approximately 1.33 and 1.28, while the zeolite frameworks had net charges of −5.33 and −5.12 for the T12–T11 and silicalite models, respectively. No proton transfer was observed. The smaller intermolecular C–C distances imply that the pronounced elongation along the **b** unit cell vector is due to electrostatic repulsion between these larger organic molecules inside the pore. The study of charges demonstrates that electrostatic interactions between adsorbates and between adsorbates and the zeolite framework are the primary drivers influencing the structural deformation of the H-ZSM-5 framework.

The volume changes upon coke precursor adsorption were analyzed for each model of the zeolite H-ZSM-5 (Supporting Information 4). Inconsistent trends were observed, depending on the size of the coke precursor molecules. Upon adsorption of molecules with a kinetic diameter ≤6.2 Å, a volume reduction is observed. This change can be attributed to the contractions along the (*a*) and (*c*) unit cell parameters, driven by attractive forces between the zeolite oxygen atoms and coke molecules. The introduction of larger molecules into the zeolite framework initially results in volume contraction with a loading of up to two molecules per unit cell, but further molecule additions lead to unit cell expansion and volume increase. The cell expansion is due to the elongation of the (*b*) unit cell parameter, attributed to the repulsive forces between larger molecules along the straight channel. These inconsistent trends in the unit cell volume (UCV) with different guest species underscore why UCV can be an unreliable XRD descriptor of zeolite H-ZSM-5 deactivation.^35^

#### The Magnitude of Guest-Induced Flexibility

To systematically understand how various types and amounts of coke precursors influence the zeolite’s structural flexibility, we focused on two key parameters: Δ(*a*–*b*) and Δ(*D*_max_/*D*_min_). These parameters represent the relative magnitude of structural changes at different coke loadings and were calculated by subtracting the parameter values of the zeolite containing guest molecules from those of the empty zeolite, as outlined in eq 4.
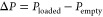
4

where P is the flexibility parameter of interest: (*a*–*b*) or (*D*_max_/*D*_min_).

**Figure 8:**
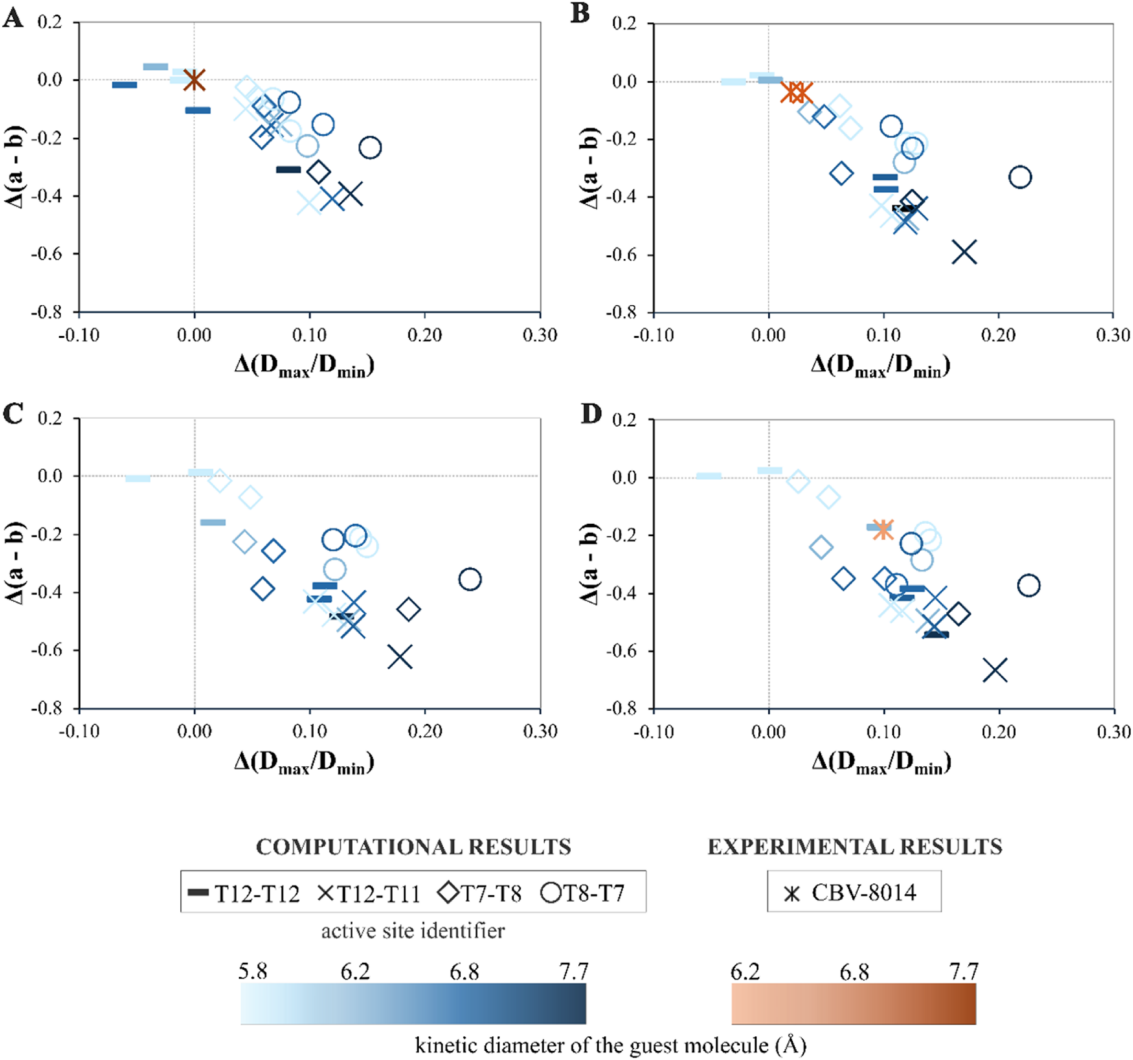
Scatter plot representing differences in local and global flexibility descriptors between loaded zeolite with (A) one molecule/uc, (B) two molecules/uc, (C) three molecules/uc, and (D) four molecules/uc and empty material.

The plots shown in Figure 8 illustrate the changes in both flexibility descriptors. As previously determined from examining the relationship between local and global flexibility descriptors depending on the type and amount of various coke precursors (Figure 6), we observed that the change in the (*D*_max_/*D*_min_) parameter is predominantly positive when comparing loaded and empty structures, whereas the change in the (*a*–*b*) parameter is predominantly negative for most structures. Independent of the active site location, number of coke molecules, or type of coke species, the relationship between changes in the local and global flexibility descriptors is both inverse and linear.

The insertion of the first organic molecule already triggers noticeable changes in the structure. As depicted in Figure 8A, the data points are primarily grouped based on the location of the active sites rather than the size of the guest molecule. Consequently, the arrangement of the active sites exerts a stronger influence on the framework’s response compared to the size of the guest molecule. Increasing the number of molecules generally leads to more significant structural deformations. However, no noticeable distinction is observed between adding the third and fourth coke species to the zeolite structure (Figure 8C,D). This observation could be attributed to the material’s flexibility limit being reached after filling it with three molecules per unit cell, as the same trend is apparent in the material without an active site (Supporting Information 11, Figures S9 and S10).

At a loading of 3 and 4 molecules per unit of charge (uc), distinct clustering of quantified structural changes is observed, depending on the active site position. This is particularly apparent for models with the same Al position, such as T12–T12 (represented by minuses in Figure 8) versus T12–T11 (represented by crosses in Figure 8). In the T12–T12 model, the framework’s response is significantly influenced by the size of the guest molecule, with smaller molecules inducing less material distortion compared to larger molecules of kinetic diameter larger than or equal to 6.2 Å. In the T12–T11 model, the data show tighter clustering, indicating that the framework’s response remains similar regardless of the size of the guest molecule. This discrepancy can primarily be attributed to the position of the active site; when the proton is located between two 10-member rings of the straight channel, the effect of smaller molecules on the framework is negligible.

Variations in structural response are also evident in H-ZSM-5 models with the same proton location but differing aluminum distributions (T7–T8 vs T8–T7). In the T7–T8 model (represented by diamonds in Figure 8), local distortions are negligible, regardless of the coke precursor. However, the (*a*–*b*) parameter decreases significantly as the size of the guest species increases. This structure also has the lowest electronic energy for an empty zeolite, suggesting lower flexibility and greater stability with less distorted 10-member ring shapes. This insight is not apparent from atomic coordinate deviations alone (Figure 5). In the model with active sites between Al8 and Si7 (represented by open circles in Figure 8), data points cluster similarly to the T12–T11 model but show smaller global structural changes. Comparing NRMSD values (Figure 5) between these models reveals minimal differences in atomic position deviations, emphasizing the necessity for diverse flexibility descriptors to fully understand the structural responses of the framework to various coke precursors.

### Experimental Section

#### Catalyst Characterization

**Figure 9:**
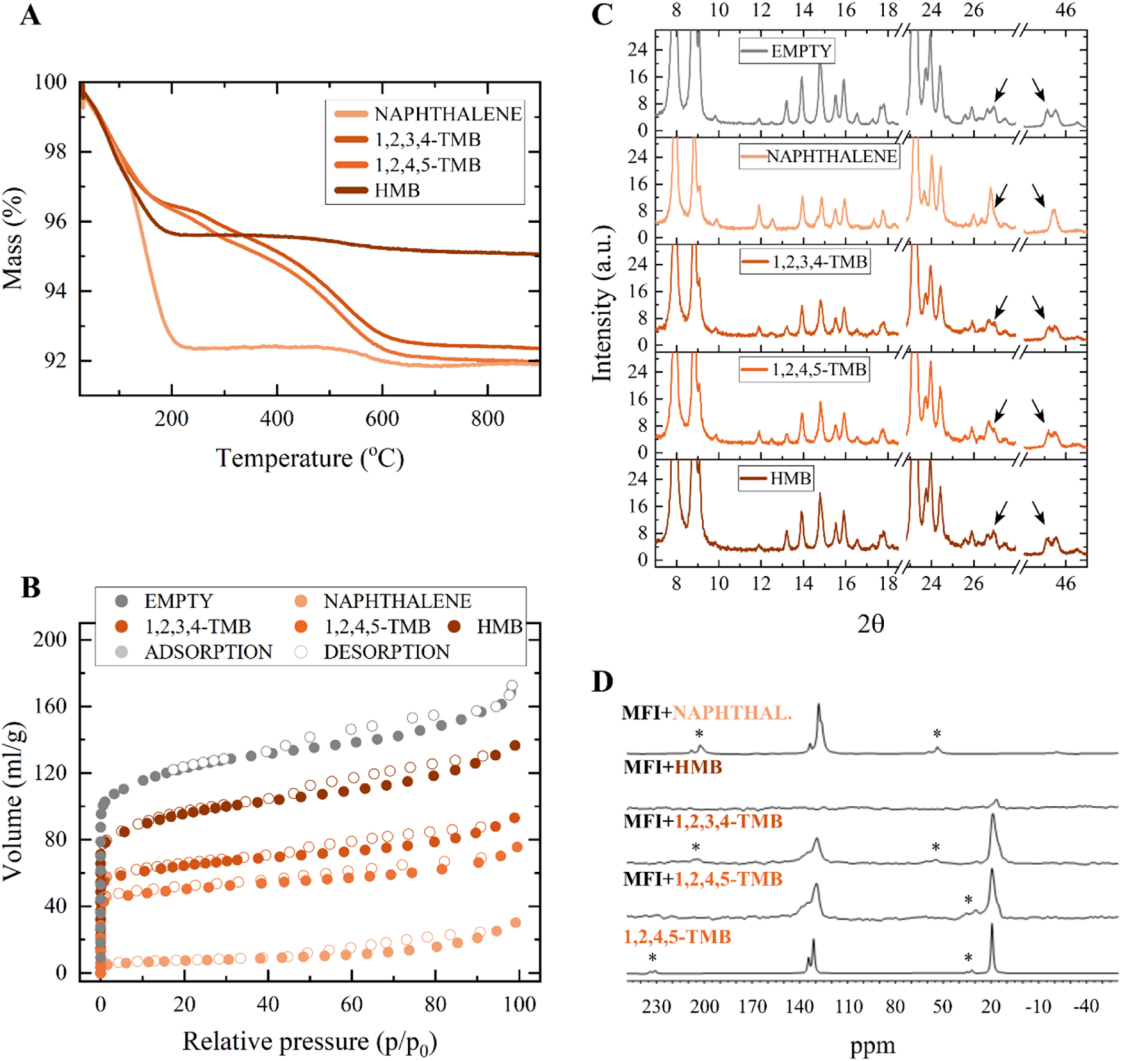
(A) Thermal analysis, (B) N_2_ adsorption isotherms of empty (commercial) and loaded zeolite samples, (C) zoom in of the X-ray diffractograms of empty zeolite H-ZSM-5 and zeolite H-ZSM-5 after adsorption experiment with different organic molecules. (D) ^13^C {^1^H–^13^C CP} NMR spectra of H-ZSM-5 (MFI) with different organic molecules. The bottom spectrum is pure 1,2,4,5-TMB. Asterisks mark the spinning sidebands.

To investigate trends in zeolite flexibility upon guest adsorption, we conducted an adsorption experiment using naphthalene, prehnitene (1,2,3,4-TMB), durene (1,2,4,5-TMB), and hexamethylbenzene as coke precursors with a commercially available H-ZSM-5 zeolite catalyst. Various techniques, such as thermal analysis, nitrogen adsorption, X-ray diffraction, and solid-state ^13^C NMR, were employed to assess the adsorption capabilities of these molecules and their influence on the MFI-type zeolite structure. The results of these analyses are listed in Figure 9.

Thermogravimetric analysis was conducted to quantitatively analyze the composition of each sample (Figure 9A). The zeolite containing naphthalene as a guest species exhibited a significant and rapid mass reduction upon heating, indicating nearly complete desorption of naphthalene around 210 °C. The zeolite sample with adsorbed hexamethylbenzene displayed a mass reduction until approximately 200 °C, primarily due to the desorption of organic species. Distinct behaviors were observed for both samples containing the two tetramethylbenzene isomers. Heating these samples led to a rapid mass reduction until around 180 °C, mainly due to the removal of organic molecules. This was followed by a much slower yet consistent desorption of guest species. This differing behavior could be attributed to the slower diffusion of bulkier molecules (compared to naphthalene) or to chemical reactions occurring within the pores of the zeolite, such as transalkylation or oligomerization of guest molecules, considering that prior to any characterization, the samples were kept at 200 °C for 100 h. For all hydrocarbons, an additional mass drop is observed around 600 °C, attributed to the combustion of carbonaceous species formed during the heating of the samples from the remaining coke precursor. Assuming the samples were water free before TGA, we estimated that the zeolite samples were loaded with between 3 and 4 naphthalene molecules per unit cell, with almost 3 molecules per unit cell of 1,2,3,4-TMB and a little over 3 molecules per unit cell of 1,2,4,5-TMB, and almost 2 hexamethylbenzene molecules per unit cell.

Thermal analysis provides accurate quantification of guest species loading; however, there is little information about the location of these molecules. To distinguish whether they are adsorbed inside the zeolite channels or simply deposited at the surface, the specific surface area of the materials was calculated by applying the Brunauer–Emmett–Teller (BET)^36^ method to nitrogen adsorption isotherms (Figure 9B). Pure H-ZSM-5 presents a high specific surface area (*a*_BET_) of around 450 m^2^ g^–1^. The sample with hexamethylbenzene has a slightly reduced specific surface area (*a*_BET_ = 347 m^2^ g^–1^) compared to the empty zeolite, suggesting the presence of some guest species inside the pores, albeit at a relatively low loading. Higher loading is observed for samples with 1,2,3,4-TMB and with 1,2,4,5-TMB, where the BET surface area was reduced to 240 and 190 m^2^ g^–1^, respectively, indicating an increased number of molecules blocking pores. Finally, zeolite loaded with naphthalene shows a very limited capacity for nitrogen adsorption with a BET surface area of only 26 m^2^ g^–1^. This suggests that the available pore volume is almost fully occupied by guest species. Additionally, the *t*-plot method was used to determine the micropore volumes. The empty sample had a microporous volume of 0.113 cm^3^ g^–1^. Zeolite loaded with hexamethylbenzene presents a reduced volume of 0.077 cm^3^ g^–1^. For zeolite samples loaded with 1,2,3,4-TMB and 1,2,4,5-TMB, the microporous volume decreased to 0.064 and 0.049 cm^3^ g^–1^, respectively. The naphthalene-loaded zeolite’s micropore volume was reduced by 3 orders of magnitude compared to the empty H-ZSM-5 sample, resulting in a value of 0.176 mm^3^g^–1^. These *t*-plot micropore volumes further indicate the extent to which the zeolite’s pores are filled with coke precursors.

Powder X-ray diffraction is a complementary characterization technique that allows to quantify the number of organic molecules adsorbed within the pores and to study detailed structural information and the influence of the adsorbed molecules on the zeolite’s structure.^26,40,41^ The results of the Rietveld analysis are summarized in Table 3. The measured nonframework species mass (NFSM) values provide an estimation of the number of guest molecules adsorbed within the pores of the studied H-ZSM-5 zeolite. Specifically, this catalyst was able to adsorb up to approximately 4 molecules of naphthalene per unit cell, 2 molecules of tetramethylbenzene (TMB) isomers per unit cell, and 1 molecule of hexamethylbenzene (HMB) per unit cell. When comparing these values to those obtained through TGA, we observe that nearly all of the adsorbed naphthalene and less than half of the adsorbed HMB molecules were located inside the pores of the zeolite. Additionally, about 70 wt % of TMB molecules were adsorbed into the MFI zeolite, with a slightly higher loading of durene (1,2,4,5-TMB) molecules. Both N_2_ adsorption studies and NFSM show the same trends when examining the pore loading of the zeolite H-ZSM-5.

The quality of the Rietveld analysis fit, along with full X-ray diffractograms, can be found in the Supporting Information 12 (Figure S11). The dummy carbon atom positions, identified via difference Fourier map analysis, suggest that the aromatic molecules conform to accommodate the local distortions of the straight channel cross sections and that they are mainly at the channel intersection, aligning with DFT calculations (Supporting Information 13). By zooming in on the X-ray diffractograms (Figure 9C), we can identify features indicating global framework distortions, marked by arrows at 27 and 45° 2θ. For the sample with naphthalene, the changes in the unit cell parameters are extreme. In contrast, for the zeolite loaded with either isomer of tetramethylbenzene (TMB), the peak changes are intermediate. Meanwhile, the sample with hexamethylbenzene (HMB) as the coke precursor displays a diffractogram almost identical to that of the fresh H-ZSM-5 sample. These deformations were quantified, and the results are presented in Table 3. This allows for a direct comparison between theoretical and experimentally obtained data, which is discussed later.

The NMR spectra of the zeolite framework with adsorbed species are shown in Figure 9D. The bottom spectrum serves as a reference and shows 1,2,4,5-TMB, with peaks at 131.2 and 134.5 ppm, corresponding to the aromatic carbon atoms, and a peak at 19.0 ppm from the methyl group’s carbon atoms. The applied spectral width was larger than the plotted one to observe potential protonated species (carbocations), which typically show peaks at high frequencies with chemical shifts from 200 ppm and above.^42,43^ However, no additional peaks were observed.

The spectra for MFI + TMB samples display aromatic and methyl group’s carbon atoms, as expected; however, the chemical shift range is broader compared to the reference TMB sample. The highest frequency peaks range from 129 to 138 ppm, and the methyl groups range from 15 to 19 ppm. The increased range of chemical shifts relative to the reference TMB sample indicates interactions and variations in the molecular structure and electronic distribution. The peak shifts could result from interactions between the conjugated π-system of the ring and BAS, without any direct proton transfer. If the conjugated π-system coordinates with a BAS, significant changes in the electronic structure relative to the reference TMB sample occur, causing the observed shift in the peak positions.

The MFI + HMB spectrum shows only very weak peaks, with a peak at 16.2 ppm attributed to methyl groups. According to TGA results, the HMB sample should contain a similar amount of methyl groups to the TMB samples, which is not reflected in the spectra. This discrepancy might be due to using cross-polarization (CP) from H to C for signal generation. CP, a method that relies on through-space dipole–dipole couplings between H and C to build up C’s signal intensity, includes a set of parameters to vary for collecting spectra. Various phenomena and environmental factors, such as the distance between H and C and local dynamics, influence the couplings and the signal buildup curve. Assuming the TGA data are accurate, two possible reasons for the low-intensity peaks in the HMB sample are (1) if the T_1ρ_ relaxation time for H in the HMB sample is substantially longer than in the TMB samples, we would observe lower intensity with similar acquisition parameters, and (2) a shorter total signal buildup and decay curve period compared to the other samples would also result in lower intensity. This curve is a function of *T*_1ρ_ and *T*_HC_, both related to differences in the dynamic nature of the confined molecules among the samples. A more extensive NMR study is required to resolve these differences.

For the MFI sample with naphthalene, there are three peaks, all with similar widths as the reference sample, at 126.5, 128.1, and 133.6 ppm, corresponding to the three nonequivalent carbon atoms in naphthalene. The absence of additional peaks indicates that naphthalene fits into the zeolite pores without significant distortion or interactions with the framework or the BAS.

### Integration of Experimental and Theoretical Insights

Since the experimental sample used in this study was commercial H-ZSM-5 zeolite, the actual distribution of BAS is unknown. In practice, synthesizing a zeolite with a precisely controlled distribution of active sites is inherently challenging due to the variability introduced by synthesis methods and preparation conditions.^44,45^ As a result, the distribution of acid sites in experimental samples is often more random and less optimized compared to the carefully controlled configurations used in computational models. While the computational models provide valuable insights into potential interactions between acid sites and sorbate molecules, as well as the structural response of the zeolite’s framework, they represent idealized scenarios that may not directly correspond to experimental reality. Therefore, computational results should be interpreted as complementary to experimental findings rather than definitive to allow for a more comprehensive understanding of catalyst performance through the integration of both theoretical and experimental data.

As seen in the XRD analysis, the structural behavior of the commercial H-ZSM-5 zeolite upon naphthalene adsorption aligns with theoretical predictions (Figure 8D, light orange data point). Significant structural changes are observed both in the unit cell parameters and in the shape of the straight channels. The change in the global flexibility descriptor is primarily influenced by the compression of the framework in the *a*-direction (Δ*a* = −0.6%) rather than the expansion along the straight channels (Δ*b* = 0.3%). This observation is consistent with computational models and suggests that electrostatic attraction between the zeolite framework and naphthalene molecules is the dominant interaction. Additionally, a slight compression along the *c*-direction in the naphthalene-loaded zeolite was observed (Δ*c* = −0.1%), which aligns with theoretical findings (Figure 7). Upon naphthalene adsorption, the straight channel’s shape changed from circular to elliptical.

Results obtained using the DFT method showed that, regardless of the active site model, loading two molecules of either tetramethylbenzene isomer per unit cell resulted in noticeable structural changes, both in pore shape and unit cell vector lengths (Figures 6 and 8B). However, data obtained through refinement indicate that the zeolite exhibits a relatively low distortion when exposed to TMB (Table 3). Based on the structural response of the framework and the distinct behavior observed during thermal analysis, we speculate that possible oligomerization or transalkylation reactions occurred within the pores of the zeolite, inducing a structural response that is incongruent with theoretical results.

When considering the impact of hexamethylbenzene on the zeolite framework, computational data suggest that even loading of one molecule per unit cell results in substantial structural changes (see Figures 5, 6, and Supporting Information 4). However, NFSM data estimated the HMB loading at approximately half a molecule per unit cell. Analysis of experimental results indicates that at this lower loading, no structural deformation was observed. We attribute this discrepancy to the bulkiness of hexamethylbenzene relative to the zeolite pore opening, restricting HMB to predominantly surface adsorption. This hypothesis is corroborated by the solid-state NMR results (Figure 9D). Nevertheless, the computational analysis demonstrates how the zeolite might respond to hexamethylbenzene formed during the MTH reaction.

## Conclusions

In this study, the flexibility of zeolite H-ZSM-5 upon the addition of aromatic molecules identified as internal coke precursors was investigated by using both theoretical and experimental methods. It was found that coke molecules predominantly occupy intersections between straight and sinusoidal channels, leading to significant structural changes. Notable variations were observed in unit cell vectors, represented by a decrease in the (*a*–*b*) descriptor, and the straight channel pores became more oval, as indicated by an increase in the *D*_max_/*D*_min_ descriptor.

Through theoretical models, it was shown that these structural deformations were mainly driven by electrostatic interactions between adsorbates and between adsorbates and the zeolite framework. NRMSD calculations revealed changes in the local environment of framework atoms upon adsorption, especially oxygen atoms of the straight channels. Among the four studied active site models, the T12–T11 site exhibited the lowest electronic energy, indicating high stability across various coke molecules. Nonacidic structures showed minimal flexibility, except when interacting with larger molecules (>6.8 Å).

Experimental adsorption studies with commercially available H-ZSM-5 and four aromatic compounds confirmed the theoretical predictions. Multiple characterization methods (TGA, N_2_ adsorption, XRD, and solid-state NMR) identified and quantified structural changes driven more by varying loading levels than by the specific molecular nature of the adsorbates.

Theoretical results indicated that the extent of structural deformation was closely linked to the active site location, with higher distortion observed for models with Brønsted acid site between T atoms in the same 10-member ring and lower for those between T atoms in neighboring rings.

These results demonstrate that various BAS distributions need to be considered in the computational studies of zeolite materials. The electrostatic interactions between adsorbed coke molecules and the framework as well as the realistic loading of the molecules play a critical role. Detailed examination of active site locations is essential to fully understanding the flexibility and structural responses of H-ZSM-5. The complexity of structural distortions in MFI-type zeolites has been established through these observations.

## Supporting Information Available

Convergence test; models of H-ZSM-5 with detailed Al and BAS distribution; unit cell parameters for each model; detailed RMSD data; measure of intermolecular distances; Hirshfeld charges; volume changes of selected models; details on model with no BAS; XRD data analysis; and atomic coordinates for dummy carbon atoms (PDF)
CIF files of all optimized structures (ZIP)
