# Supplementary material for: Investigating the Flexibility of H-ZSM-5 Zeolite Upon Adsorption of Coke Precursors: A Theoretical and Experimental Approach
Source: J Phys Chem C Nanomater Interfaces. 2025 Jan 2;129(2):1183–97. doi: 10.1021/acs.jpcc.4c07349 (PMC11744781; doi:10.1021/acs.jpcc.4c07349)
Supplement: Supplementary file 1 — jp4c07349_si_001.pdf [file jp4c07349_si_001.pdf]

Supporting Information

Investigating the Flexibility of H-ZSM-5 Zeolite Upon Adsorption of Coke Precursors: A Theoretical and Experimental Approach

Agnieszka Seremak[1], Izar Capel Berdiell[1], Bjørnar Arstad[2], Torstein Fjermestad[1], Stian Svelle[1*]

[1] *Center for Materials Science and Nanotechnology (SMN), Department of Chemistry, University of Oslo, P.O. Box 1033, Blindern, N-0315 Oslo, Norway*
[2] *SINTEF Industry, Forskningveien 1, Oslo 0314, Norway*

*Corresponding author

E-mail: stian.svelle@kjemi.uio.no

Supporting Information 1. Convergence tests....3
Supporting Information 2. H-ZSM-5 model with BAS between T7-T7....4
Supporting Information 3. Distribution of aluminum atoms and protons within the unit cell, and detailed depiction of the placement of coke guest species. 5
Supporting Information 4. Unit cell parameters for every zeolite H-ZSM-5 model with all studied guest molecules. 7
Supporting Information 5. Local Root Mean Square Deviation values (in Å) for individual atoms of the most distorted structure for each studied model. 17
Supporting Information 6. Average Root Mean Square Deviation values (in Å) of each studied structure with different type and number of adsorbed coke precursors....52
Supporting Information 7. Relationship between local (D_{max}/D_{min}) and global (a-b) flexibility descriptors of models sharing the same BAS location. 57
Supporting Information 8. Relative changes of unit cell parameter vectors....58
Supporting Information 9. Hirshfeld charges 59
Supporting Information 10. Volume changes of studied model upon adsorption of coke precursors 72
Supporting Information 11. Model with no active site (silicalite)....73
Supporting Information 12. XRD data analysis....74
Supporting Information 13. Atomic coordinates for dummy carbon atoms....75

Supporting Information 1. Convergence tests

Convergence test results for finding values of kinetic energy cutoff and relative cut off. Performed according to standard procedure in with CP2K software.

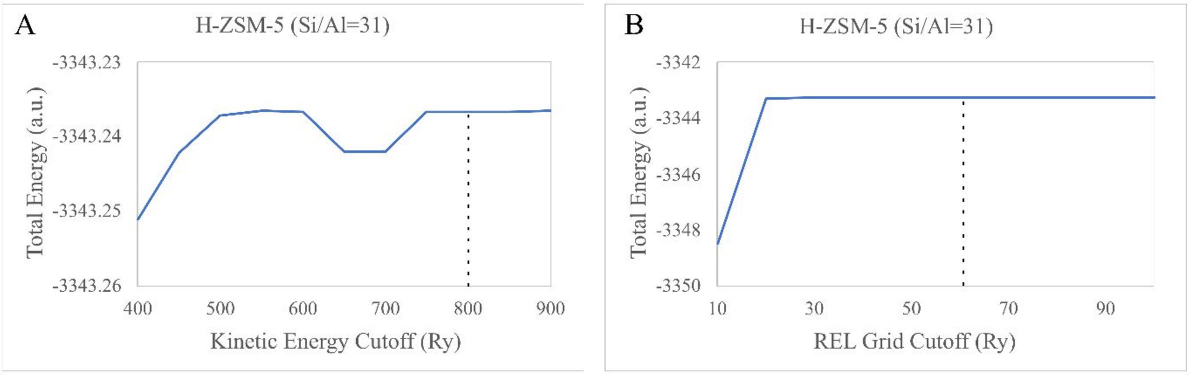

Figure S1. Convergence test for empty zeolite with three aluminum per unit cell.

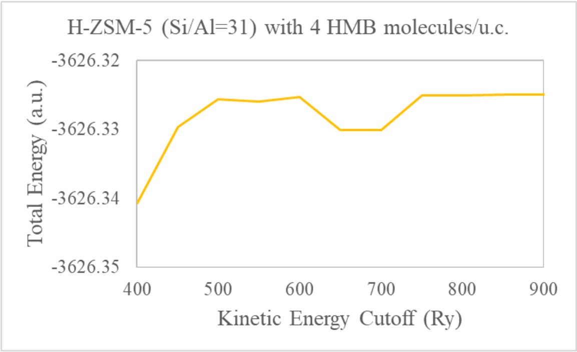

Figure S2. Convergence test for zeolite H-ZSM-5 with three aluminum per unit cell and with four hexamethylbenzenes per unit cell as guest species.

Supporting Information 2.   H-ZSM-5 model with BAS between T7-T7

Table S1. Structural parameters of empty H-ZSM-5 with active site between Al7 and Si7.

Unit cell parameters	a	20.271
	b	19.843
	c	13.419
	V	5398
Global descriptor	**a-b**	**0.428**
Local descriptor	**D_{max}/D_{min}**	**1.17**

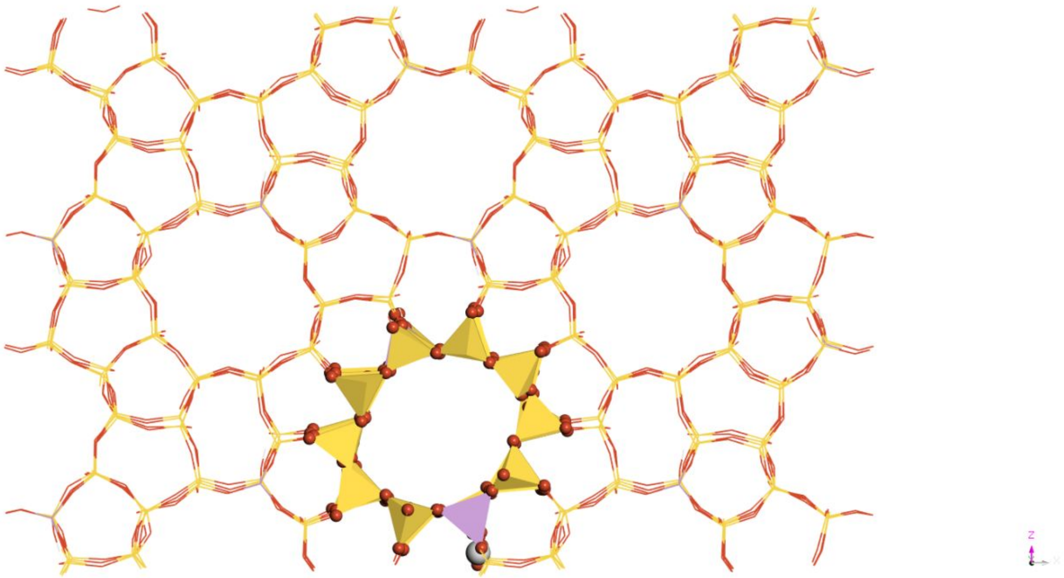

Figure S3. Snapshot of optimized zeolite H-ZSM-5 with active site between Al7 and Si7.

Supporting Information 3. Distribution of aluminum atoms and protons within the unit cell, and detailed depiction of the placement of coke guest species.

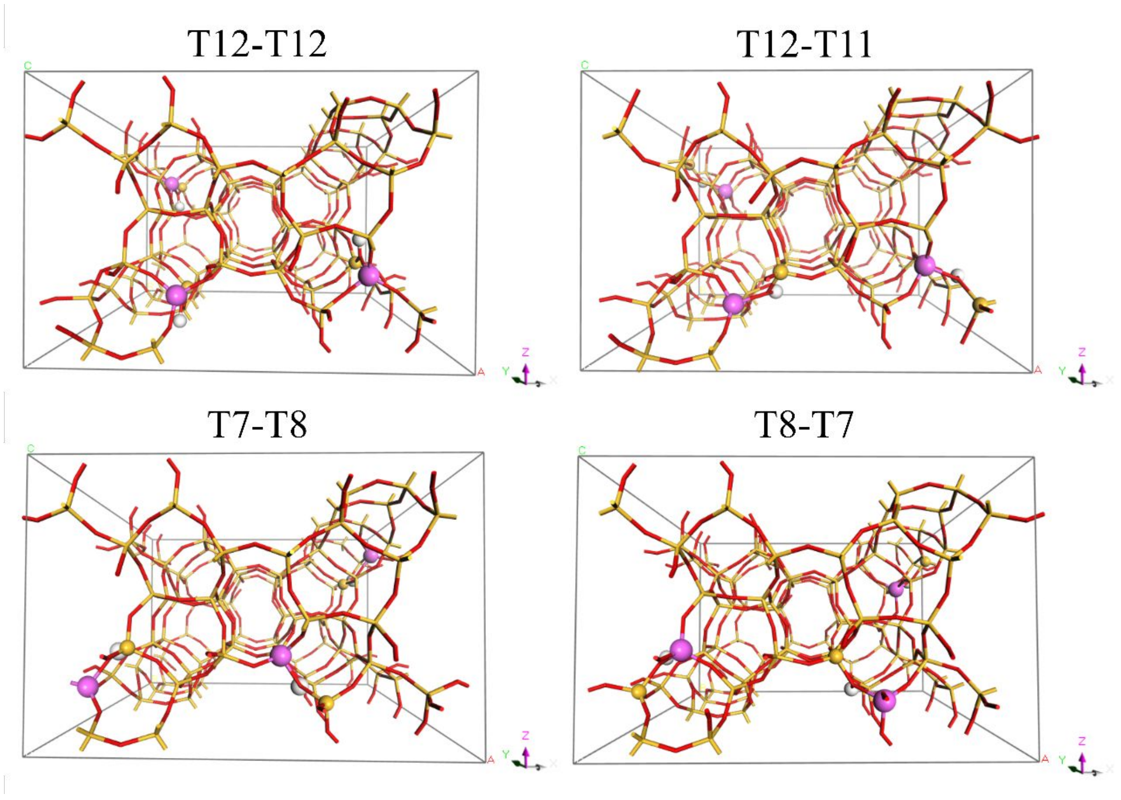

Figure S4. Images showing the distribution of Al atoms (indicated by the first T site numbering and represented by pink spheres) and protons (small white spheres) in each H-ZSM-5 model. Silicon atoms (indicated by the second T site numbering) are represented by yellow spheres.

T12-T11

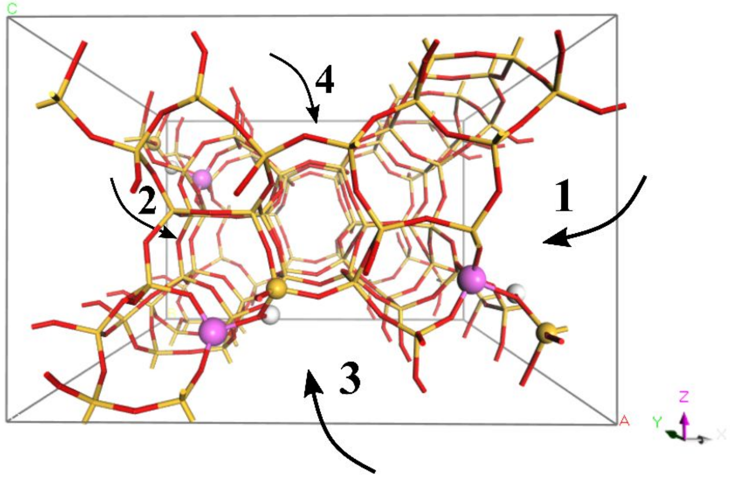

Figure S5. Graphical depiction of the placement of each subsequent coke guest species. The first two molecules are positioned along the same straight channel, while the next two are situated in an alternate straight channel. The first three coke molecules are always in the proximity to the Brønsted Acid Site.

Supporting Information 4. Unit cell parameters for every zeolite H-ZSM-5 model with all studied guest molecules.

I. Unit cell parameters for model with active site between T12-T12

a) with benzene

		Number of molecules				
		0	1	2	3	4
Unit cell parameters	a	20.289	20.299	20.299	20.292	20.292
	b	19.864	19.847	19.854	19.853	19.844
	c	13.455	13.438	13.416	13.396	13.393
	alpha	90	90	90	90	90
	beta	90	90	90	90	90
	gamma	90	90	90	90	90
	(a-b)	0.425	0.452	0.445	0.440	0.448
	V	5422.9	5413.9	5406.6	5396.5	5393.0

b) with toluene

		Number of molecules				
		0	1	2	3	4
Unit cell parameters	a	20.289	20.285	20.293	20.288	20.286
	b	19.864	19.861	19.871	19.871	19.856
	c	13.455	13.445	13.434	13.425	13.418
	alpha	90	90	90	90	90
	beta	90	90	90	90	90
	gamma	90	90	90	90	90
	(a-b)	0.425	0.424	0.422	0.417	0.430
	V	5422.9	5416.7	5417.1	5412.0	5404.7

c) with naphthalene

		Number of molecules				
		0	1	2	3	4
Unit cell parameters	a	20.289	20.262	20.258	20.146	20.084
	b	19.864	19.794	19.829	19.881	19.830
	c	13.455	13.430	13.408	13.397	13.420
	alpha	90	90	90	90	90
	beta	90	90	90	90	90
	gamma	90	90	90	90	90
	(a-b)	0.425	0.468	0.429	0.264	0.254
	V	5422.9	5386.2	5385.6	5365.6	5347.9

d) with 1,2,3,4-TMB

		Number of molecules				
		0	1	2	3	4
Unit cell parameters	a	20.289	20.194	19.982	19.965	19.972
	b	19.864	19.876	19.932	19.964	19.965
	c	13.455	13.431	13.393	13.379	13.400
	alpha	90	90	90	90	90
	beta	90	90	90	90	90
	gamma	90	90	90	90	90
	(a-b)	0.425	0.318	0.050	0.000	0.007
	V	5422.9	5390.6	5334.2	5332.8	5343.2

e) with 1,2,4,5-TMB

		Number of molecules				
		0	1	2	3	4
Unit cell parameters	a	20.289	20.245	20.012	19.976	19.970
	b	19.864	19.839	19.920	19.928	19.930
	c	13.455	13.436	13.403	13.418	13.440
	alpha	90	90	90	90	90
	beta	90	90	90	90	90
	gamma	90	90	90	90	90
	(a-b)	0.425	0.406	0.092	0.048	0.041
	V	5422.9	5396.3	5343.1	5341.5	5349.4

f) with hexamethylbenzene

		Number of molecules				
		0	1	2	3	4
Unit cell parameters	a	20.289	20.070	20.019	20.044	20.038
	b	19.864	19.956	20.032	20.103	20.158
	c	13.455	13.406	13.387	13.377	13.418
	alpha	90	90	90	90	90
	beta	90	90	90	90	90
	gamma	90	90	90	90	90
	(a-b)	0.425	0.114	-0.013	-0.058	-0.120
	V	5422.9	5369.1	5368.5	5390.0	5419.7

II. Unit cell parameters for model with active site between T12-T11

a) with benzene

		Number of molecules				
		0	1	2	3	4
Unit cell parameters	a	20.145	19.937	19.919	19.909	19.896
	b	19.849	20.062	20.052	20.041	20.040
	c	13.446	13.365	13.352	13.344	13.334
	alpha	90	90	90	90	90
	beta	90	90	90	90	90
	gamma	90	90	90	90	90
	(a-b)	0.296	-0.125	-0.133	-0.133	-0.144
	V	5376.4	5345.6	5333.1	5323.9	5316.4

b) with toluene

		Number of molecules				
		0	1	2	3	4
Unit cell parameters	a	20.145	20.094	19.903	19.879	19.886
	b	19.849	19.895	20.069	20.057	20.050
	c	13.446	13.438	13.352	13.344	13.343
	alpha	90	90	90	90	90
	beta	90	90	90	90	90
	gamma	90	90	90	90	90
	(a-b)	0.296	0.199	-0.166	-0.178	-0.164
	V	5376.4	5372.2	5333.3	5320.7	5320.0

c) with naphthalene

		Number of molecules				
		0	1	2	3	4
Unit cell parameters	a	20.145	20.048	19.883	19.862	19.857
	b	19.849	19.907	20.060	20.059	20.054
	c	13.446	13.442	13.363	13.365	13.369
	alpha	90	90	90	90	90
	beta	90	90	90	90	90
	gamma	90	90	90	90	90
	(a-b)	0.296	0.142	-0.177	-0.196	-0.197
	V	5376.4	5364.7	5329.9	5324.8	5323.5

d) with 1,2,3,4-TMB

		Number of molecules				
		0	1	2	3	4
Unit cell parameters	a	20.145	20.083	19.904	19.880	19.888
	b	19.849	19.940	20.094	20.099	20.105
	c	13.446	13.416	13.351	13.353	13.363
	alpha	90	90	90	90	90
	beta	90	90	90	90	90
	gamma	90	90	90	90	90
	(a-b)	0.296	0.144	-0.190	-0.219	-0.217
	V	5376.4	5372.5	5339.5	5335.2	5343.0

e) with 1,2,4,5-TMB

		Number of molecules				
		0	1	2	3	4
Unit cell parameters	a	20.145	19.946	19.920	19.922	19.934
	b	19.849	20.057	20.062	20.058	20.052
	c	13.446	13.399	13.429	13.457	13.468
	alpha	90	90	90	90	90
	beta	90	90	90	90	90
	gamma	90	90	90	90	90
	(a-b)	0.296	-0.111	-0.142	-0.136	-0.118
	V	5376.4	5360.4	5366.5	5377.0	5383.4

f) with hexamethylbenzene

		Number of molecules				
		0	1	2	3	4
Unit cell parameters	a	20.145	19.943	19.874	19.880	19.873
	b	19.849	20.037	20.166	20.204	20.240
	c	13.446	13.411	13.382	13.381	13.399
	alpha	90	90	90	90	90
	beta	90	90	90	90	90
	gamma	90	90	90	90	90
	(a-b)	0.296	-0.094	-0.292	-0.324	-0.367
	V	5376.4	5358.9	5363.3	5374.5	5389.5

III. Unit cell parameters for model with active site between T7-T8

a) with benzene

		Number of molecules				
		0	1	2	3	4
Unit cell parameters	a	20.143	20.100	20.069	20.126	20.112
	b	19.876	19.855	19.885	19.873	19.859
	c	13.436	13.371	13.344	13.315	13.302
	alpha	90	90	90	90	90
	beta	90	90	90	90	90
	gamma	90	90	90	90	90
	(a-b)	0.268	0.245	0.183	0.253	0.254
	V	5379.5	5336.5	5325.1	5325.4	5312.8

b) with toluene

		Number of molecules				
		0	1	2	3	4
Unit cell parameters	a	20.143	20.077	20.034	20.096	20.090
	b	19.876	19.866	19.928	19.901	19.891
	c	13.436	13.370	13.341	13.316	13.308
	alpha	90	90	90	90	90
	beta	90	90	90	90	90
	gamma	90	90	90	90	90
	(a-b)	0.268	0.211	0.106	0.195	0.199
	V	5379.5	5332.5	5326.3	5325.4	5318.0

c) with naphthalene

		Number of molecules				
		0	1	2	3	4
Unit cell parameters	a	20.143	20.053	20.102	20.049	20.050
	b	19.876	19.895	19.939	20.007	20.023
	c	13.436	13.372	13.376	13.353	13.354
	alpha	90	90	90	90	90
	beta	90	90	90	90	90
	gamma	90	90	90	90	90
	(a-b)	0.268	0.158	0.163	0.041	0.028
	V	5379.5	5334.7	5361.4	5356.2	5361.2

d) with 1,2,3,4-TMB

		Number of molecules				
		0	1	2	3	4
Unit cell parameters	a	20.143	20.062	20.006	20.013	20.037
	b	19.876	19.990	20.056	20.131	20.117
	c	13.436	13.410	13.393	13.385	13.400
	alpha	90	90	90	90	90
	beta	90	90	90	90	90
	gamma	90	90	90	90	90
	(a-b)	0.268	0.072	-0.050	-0.118	-0.080
	V	5379.5	5378.0	5373.5	5392.9	5401.2

e) with 1,2,4,5-TMB

		Number of molecules				
		0	1	2	3	4
Unit cell parameters	a	20.143	20.092	20.110	20.088	20.033
	b	19.876	19.912	19.965	20.076	20.115
	c	13.436	13.377	13.398	13.401	13.379
	alpha	90	90	90	90	90
	beta	90	90	90	90	90
	gamma	90	90	90	90	90
	(a-b)	0.268	0.180	0.145	0.012	-0.082
	V	5379.5	5351.6	5379.0	5404.6	5391.6

f) with hexamethylbenzene

		Number of molecules				
		0	1	2	3	4
Unit cell parameters	a	20.143	19.998	19.977	19.957	19.971
	b	19.876	20.047	20.124	20.147	20.174
	c	13.436	13.364	13.355	13.376	13.392
	alpha	90	90	90	90	90
	beta	90	90	90	90	90
	gamma	90	90	90	90	90
	(a-b)	0.268	-0.049	-0.148	-0.190	-0.203
	V	5379.5	5357.5	5369.0	5378.0	5395.6

IV. Unit cell parameters for model with active site between T8-T7

a) with benzene

		Number of molecules				
		0	1	2	3	4
Unit cell parameters	a	20.158	20.141	20.031	19.990	20.015
	b	19.908	19.954	19.995	19.980	19.954
	c	13.411	13.381	13.356	13.336	13.285
	alpha	90	90	90	90	90
	beta	90	90	90	90	90
	gamma	90	90	90	90	90
	(a-b)	0.250	0.187	0.036	0.010	0.061
	V	5382.0	5377.6	5349.3	5326.7	5306.0

b) with toluene

		Number of molecules				
		0	1	2	3	4
Unit cell parameters	a	20.158	20.026	20.011	20.003	19.992
	b	19.908	19.950	19.974	19.964	19.956
	c	13.411	13.325	13.298	13.286	13.283
	alpha	90	90	90	90	90
	beta	90	90	90	90	90
	gamma	90	90	90	90	90
	(a-b)	0.250	0.076	0.037	0.039	0.036
	V	5382.0	5323.6	5315.2	5305.7	5299.4

c) with naphthalene

		Number of molecules				
		0	1	2	3	4
Unit cell parameters	a	20.158	19.998	19.953	19.956	19.982
	b	19.908	19.974	19.981	20.025	20.016
	c	13.411	13.315	13.299	13.334	13.341
	alpha	90	90	90	90	90
	beta	90	90	90	90	90
	gamma	90	90	90	90	90
	(a-b)	0.250	0.024	-0.027	-0.069	-0.034
	V	5382.0	5318.7	5302.1	5328.3	5335.5

d) with 1,2,3,4-TMB

		Number of molecules				
		0	1	2	3	4
Unit cell parameters	a	20.158	20.154	20.117	20.101	20.095
	b	19.908	19.980	20.019	20.069	20.072
	c	13.411	13.409	13.419	13.410	13.423
	alpha	90	90	90	90	90
	beta	90	90	90	90	90
	gamma	90	90	90	90	90
	(a-b)	0.250	0.174	0.098	0.032	0.023
	V	5382.0	5399.6	5403.9	5409.7	5414.3

e) with 1,2,4,5-TMB

		Number of molecules				
		0	1	2	3	4
Unit cell parameters	a	20.158	20.088	20.037	20.051	20.063
	b	19.908	19.989	20.018	20.003	20.073
	c	13.411	13.365	13.381	13.387	13.390
	alpha	90	90	90	90	90
	beta	90	90	90	90	90
	gamma	90	90	90	90	90
	(a-b)	0.250	0.099	0.019	0.048	-0.010
	V	5382.0	5366.6	5367.1	5369.0	5392.4

f) with hexamethylbenzene

		Number of molecules				
		0	1	2	3	4
Unit cell parameters	a	20.158	20.051	20.009	20.004	19.996
	b	19.908	20.034	20.090	20.107	20.117
	c	13.411	13.379	13.355	13.377	13.404
	alpha	90	90	90	90	90
	beta	90	90	90	90	90
	gamma	90	90	90	90	90
	(a-b)	0.250	0.018	-0.081	-0.104	-0.121
	V	5382.0	5374.5	5368.4	5380.7	5391.9

V. Unit cell parameters for model without active sites (silicalite)

a) with benzene

		Number of molecules				
		0	1	2	3	4
Unit cell parameters	a	20.244	20.237	20.233	20.230	20.242
	b	19.797	19.795	19.779	19.761	19.752
	c	13.378	13.377	13.368	13.356	13.363
	alpha	90	90	90	90	90
	beta	90	90	90	90	90
	gamma	90	90	90	90	90
	(a-b)	0.447	0.442	0.455	0.469	0.490
	V	5361.6	5358.5	5349.9	5339.1	5342.9

b) with toluene

		Number of molecules				
		0	1	2	3	4
Unit cell parameters	a	20.244	20.257	20.236	20.235	20.211
	b	19.797	19.796	19.750	19.764	19.741
	c	13.378	13.389	13.362	13.364	13.355
	alpha	90	90	90	90	90
	beta	90	90	90	90	90
	gamma	90	90	90	90	90
	(a-b)	0.447	0.461	0.486	0.471	0.470
	V	5361.6	5369.3	5340.2	5344.4	5328.3

c) with naphthalene

		Number of molecules				
		0	1	2	3	4
Unit cell parameters	a	20.244	20.205	20.172	20.142	20.127
	b	19.797	19.855	19.867	19.881	19.893
	c	13.378	13.382	13.380	13.384	13.388
	alpha	90	90	90	90	90
	beta	90	90	90	90	90
	gamma	90	90	90	90	90
	(a-b)	0.447	0.350	0.305	0.262	0.234
	V	5361.6	5368.4	5362.1	5359.7	5360.7

d) with 1,2,3,4-TMB

		Number of molecules				
		0	1	2	3	4
Unit cell parameters	a	20.244	20.202	20.054	19.946	19.946
	b	19.797	19.916	19.992	19.986	20.002
	c	13.378	13.418	13.360	13.363	13.360
	alpha	90	90	90	90	90
	beta	90	90	90	90	90
	gamma	90	90	90	90	90
	(a-b)	0.447	0.285	0.062	-0.040	-0.056
	V	5361.6	5398.6	5356.0	5327.3	5330.1

e) with 1,2,4,5-TMB

		Number of molecules				
		0	1	2	3	4
Unit cell parameters	a	20.244	20.216	20.177	20.156	20.156
	b	19.797	19.846	19.956	19.988	20.016
	c	13.378	13.385	13.383	13.404	13.424
	alpha	90	90	90	90	90
	beta	90	90	90	90	90
	gamma	90	90	90	90	90
	(a-b)	0.447	0.369	0.221	0.168	0.140
	V	5361.6	5370.4	5388.8	5400.1	5415.9

f) with hexamethylbenzene

		Number of molecules				
		0	1	2	3	4
Unit cell parameters	a	20.244	20.040	20.028	19.952	20.020
	b	19.797	20.009	20.053	20.056	20.044
	c	13.378	13.396	13.381	13.409	13.405
	alpha	90	90	90	90	90
	beta	90	90	90	90	90
	gamma	90	90	90	90	90
	(a-b)	0.447	0.031	-0.025	-0.103	-0.024
	V	5361.6	5371.7	5374.4	5365.7	5379.4

Supporting Information 5. Local Root Mean Square Deviation values (in Å) for individual atoms of the most distorted structure for each studied model.

Table S2. Crystallographic and RMSD data for model T12-T12 with four naphthalene molecules.

a	20.084
b	19.83
c	13.42

atom	*atom label*	*x*	*y*	*z*	*RMSD$_i$ (Å)*
O	O1	0.47792	0.06462	0.72014	*2.48*
O	O2	0.34761	0.07166	0.72005	*1.99*
O	O3	0.41659	0.13745	0.58458	*2.02*
O	O4	0.41104	0.00318	0.57659	*2.48*
O	O5	0.30356	0.0405	0.89854	*1.69*
O	O6	0.28846	0.95398	0.74727	*2.19*
O	O7	0.21889	0.06731	0.75263	*1.91*
O	O8	0.29066	0.12412	0.04958	*1.67*
O	O9	0.19336	0.03507	0.00756	*1.78*
O	O10	0.30798	0.99325	0.07988	*1.55*
O	O11	0.09868	0.12421	0.05938	*2.04*
O	O12	0.08666	0.05295	0.89645	*1.50*
O	O13	0.09402	0.0646	0.6984	*1.86*
O	O14	0.0458	0.95336	0.77829	*0.89*
O	O15	0.17575	0.14523	0.60252	*2.07*
O	O16	0.49097	0.83425	0.69633	*2.25*
O	O17	0.36362	0.84369	0.74229	*1.91*
O	O18	0.29272	0.87222	0.90121	*1.69*
O	O19	0.23138	0.83472	0.73361	*1.74*
O	O20	0.20035	0.83224	0.02834	*1.91*
O	O21	0.10949	0.868	0.89614	*2.20*
O	O22	0.10035	0.84396	0.70364	*1.22*
Si	Si1	0.41288	0.0686	0.64962	*1.67*
Si	Si2	0.28971	0.03305	0.78123	*2.44*
Si	Si3	0.27325	0.05027	0.01045	*1.39*
Si	Si4	0.11454	0.05154	0.00937	*1.74*
Si	Si5	0.05212	0.03451	0.79129	*0.37*
Si	Si6	0.16904	0.0728	0.65733	*1.98*
Si	Si7	0.41598	0.82457	0.65391	*2.10*
Si	Si8	0.29446	0.87581	0.78135	*1.92*
Si	Si9	0.27909	0.82898	0.00185	*1.65*
Si	Si10	0.12261	0.82607	0.99848	*2.90*
Si	Si11	0.06099	0.87496	0.79989	*2.47*
Si	Si12	0.17219	0.83404	0.65251	*1.30*

**O**	**O23**	0.00935	0.92857	0.21381	***3.11***
O	O24	0.13947	0.93356	0.22422	*1.51*
O	O25	0.0791	0.85852	0.08783	*1.96*
O	O26	0.07608	0.99259	0.07139	*2.32*
O	O27	0.19058	0.95731	0.39929	*2.08*
O	O28	0.20604	0.0479	0.25259	*2.65*
O	O29	0.26947	0.93086	0.24627	*1.15*
O	O30	0.18478	0.88305	0.55929	*1.17*
O	O31	0.29611	0.94965	0.51966	*2.45*
O	O32	0.1869	0.01443	0.57696	*2.16*
O	O33	0.4014	0.87119	0.55673	*2.30*
O	O34	0.39745	0.94849	0.39722	*1.67*
O	O35	0.39664	0.93874	0.20058	*0.78*
O	O36	0.45356	0.0448	0.28702	*2.70*
O	O37	0.31974	0.86063	0.09431	*2.30*
O	O38	0.00246	0.16421	0.18653	*1.64*
O	O39	0.1261	0.15352	0.24894	*2.01*
O	O40	0.19679	0.12653	0.40888	*1.82*
O	O41	0.2577	0.17217	0.24544	*2.63*
O	O42	0.29439	0.16751	0.52948	*1.99*
O	O43	0.38178	0.12945	0.39411	*2.36*
O	O44	0.39534	0.15002	0.19966	*1.67*
Si	Si13	0.07689	0.92938	0.14874	*1.40*
Si	Si14	0.20165	0.96852	0.28068	*2.68*
Si	Si15	0.21596	0.95231	0.51313	*1.57*
Si	Si16	0.37647	0.94331	0.51245	*2.39*
Si	Si17	0.43832	0.96502	0.2958	*2.04*
Si	Si18	0.32291	0.9315	0.15466	*2.30*
Si	Si19	0.07945	0.17153	0.15383	*0.94*
Si	Si20	0.19842	0.12676	0.28736	*1.91*
Si	Si21	0.21537	0.17202	0.50492	*2.15*
Si	Si22	0.37199	0.17093	0.49658	*1.86*
Si	Si23	0.43179	0.12344	0.29775	*2.21*
Al	Al1	0.31734	0.1671	0.15332	*2.40*
O	O45	0.51162	0.56813	0.29345	*1.91*
O	O46	0.6417	0.56806	0.28086	*1.65*
O	O47	0.58113	0.63706	0.42306	*2.41*
O	O48	0.58182	0.50247	0.42957	*2.27*
O	O49	0.69215	0.54622	0.1049	*1.22*
O	O50	0.71018	0.45543	0.25089	*2.15*
O	O51	0.77199	0.57243	0.25763	*1.38*
O	O52	0.6977	0.62478	0.94651	*1.26*
O	O53	0.79836	0.54103	0.98825	*2.14*

O	O54	0.68286	0.49322	0.92656	*1.53*
O	O55	0.90097	0.62351	0.95392	*2.28*
O	O56	0.90006	0.5443	0.11122	*2.37*
O	O57	0.89821	0.56263	0.30719	*1.06*
O	O58	0.95103	0.45183	0.23297	*1.19*
O	O59	0.82158	0.64422	0.4092	*2.00*
O	O60	0.50359	0.33253	0.3157	*2.65*
O	O61	0.63061	0.34941	0.26977	*1.72*
O	O62	0.70038	0.36736	0.10592	*1.83*
O	O63	0.76259	0.33404	0.27318	*2.44*
O	O64	0.79644	0.33072	0.98271	*1.95*
O	O65	0.88786	0.36803	0.11256	*2.37*
O	O66	0.89333	0.3429	0.30548	*2.50*
Si	Si24	0.57965	0.5684	0.35748	*2.45*
Si	Si25	0.70404	0.53523	0.22267	*1.32*
Si	Si26	0.7184	0.5519	0.99103	*1.56*
Si	Si27	0.87827	0.55044	0.99592	*1.95*
Si	Si28	0.93975	0.53198	0.21449	*2.50*
Si	Si29	0.8241	0.57295	0.35063	*2.24*
**Si**	**Si30**	0.57936	0.32485	0.35678	***3.02***
Si	Si31	0.70048	0.3761	0.22515	*2.20*
Si	Si32	0.71728	0.3259	0.00487	*2.93*
Si	Si33	0.87444	0.32508	0.01126	*2.77*
Si	Si34	0.93465	0.37354	0.21093	*2.86*
Si	Si35	0.82118	0.33226	0.35527	*1.80*
O	O67	0.98462	0.42878	0.79218	*1.34*
O	O68	0.85437	0.42726	0.78088	*2.35*
O	O69	0.91706	0.35738	0.9206	*2.46*
O	O70	0.9146	0.49155	0.93171	*2.46*
**O**	**O71**	0.80326	0.45682	0.6065	***3.14***
O	O72	0.7953	0.54551	0.7557	*2.17*
O	O73	0.72434	0.433	0.75995	*2.57*
O	O74	0.80746	0.3816	0.44792	*2.34*
O	O75	0.69667	0.44946	0.48779	*1.34*
O	O76	0.80536	0.51334	0.42859	*1.79*
O	O77	0.59114	0.37072	0.45584	*2.39*
O	O78	0.59515	0.45187	0.61103	*2.42*
O	O79	0.59764	0.43217	0.80637	*2.54*
O	O80	0.54753	0.54532	0.73392	*2.65*
O	O81	0.67878	0.35956	0.91189	*2.38*
O	O82	0.99272	0.66418	0.82129	*2.08*
O	O83	0.86631	0.65682	0.76816	*2.33*
O	O84	0.79993	0.62867	0.60321	*2.59*

O	O85	0.73376	0.66395	0.7649	*1.28*
O	O86	0.70256	0.66609	0.48125	*1.95*
O	O87	0.61382	0.62713	0.61397	*2.42*
O	O88	0.60559	0.65419	0.80653	*1.90*
Si	Si36	0.91705	0.42703	0.85698	*2.32*
Si	Si37	0.79419	0.46628	0.72548	*2.06*
Si	Si38	0.77694	0.4513	0.49308	*2.57*
Si	Si39	0.61626	0.44373	0.49614	*1.79*
Si	Si40	0.55664	0.46517	0.71523	*2.66*
Si	Si41	0.67166	0.42995	0.85221	*2.51*
Si	Si42	0.91723	0.67252	0.85991	*2.18*
Si	Si43	0.7986	0.62453	0.72363	*2.09*
Si	Si44	0.78154	0.67273	0.50509	*1.66*
Si	Si45	0.6247	0.67008	0.51229	*2.01*
Si	Si46	0.56569	0.62337	0.71185	*2.29*
Si	Si47	0.67813	0.6725	0.85276	*2.50*
O	O89	0.50835	0.92489	0.2942	*2.03*
O	O90	0.63863	0.93205	0.28024	*1.75*
O	O91	0.58147	0.85538	0.41683	*2.50*
O	O92	0.57474	0.98937	0.43455	*2.73*
O	O93	0.68907	0.95123	0.10308	*0.26*
O	O94	0.70577	0.04491	0.24649	*2.20*
O	O95	0.76868	0.92795	0.25585	*1.94*
O	O96	0.69402	0.87637	0.94038	*2.00*
O	O97	0.79609	0.95533	0.98797	*1.09*
O	O98	0.68202	0.00877	0.92719	*2.72*
O	O99	0.90137	0.87628	0.95036	*2.09*
O	O100	0.89961	0.95466	0.10795	*2.19*
O	O101	0.89584	0.93448	0.30363	*2.13*
O	O102	0.95087	0.04596	0.23241	*1.24*
O	O103	0.81825	0.85687	0.40791	*2.33*
O	O104	0.49876	0.16684	0.32157	*2.68*
O	O105	0.62037	0.14705	0.25653	*2.04*
O	O106	0.68989	0.12664	0.09491	*2.45*
O	O107	0.75172	0.17091	0.25881	*2.12*
O	O108	0.79427	0.16564	0.98438	*2.00*
O	O109	0.88871	0.12432	0.10585	*1.98*
O	O110	0.89201	0.15806	0.29713	*1.36*
Si	Si48	0.57688	0.92647	0.35676	*2.14*
Si	Si49	0.70055	0.96509	0.22071	*2.01*
Si	Si50	0.71575	0.94774	0.98943	*0.85*
Si	Si51	0.87621	0.94854	0.99367	*1.03*
Si	Si52	0.93799	0.96674	0.21265	*2.07*

Si	Si53	0.82138	0.92817	0.34801	*2.52*
Si	Si54	0.57649	0.16946	0.35254	*2.33*
Si	Si55	0.69353	0.12388	0.21617	*2.48*
Si	Si56	0.71453	0.17067	0.00072	*2.29*
Si	Si57	0.87303	0.16997	0.0093	*2.72*
Si	Si58	0.93314	0.12434	0.20854	*2.23*
Al	Al2	0.81396	0.16723	0.34763	*2.67*
O	O111	0.97843	0.06706	0.78395	*2.99*
O	O112	0.8479	0.07245	0.77799	*1.79*
O	O113	0.9137	0.14166	0.91347	*2.08*
O	O114	0.9101	0.00767	0.92726	*1.57*
O	O115	0.79924	0.03763	0.60379	*2.57*
O	O116	0.79355	0.95226	0.75754	*2.18*
O	O117	0.71808	0.0621	0.75353	*1.55*
O	O118	0.7911	0.12116	0.45065	*1.57*
O	O119	0.69143	0.03514	0.4916	*2.56*
O	O120	0.80592	0.98959	0.42322	*1.63*
O	O121	0.59323	0.12193	0.44794	*2.53*
O	O122	0.58704	0.04839	0.60992	*2.21*
O	O123	0.59282	0.06617	0.80705	*1.99*
O	O124	0.54644	0.95191	0.73498	*2.13*
O	O125	0.67813	0.14186	0.90181	*2.56*
O	O126	0.99191	0.8346	0.81551	*1.86*
O	O127	0.86562	0.84066	0.76559	*1.49*
O	O128	0.80165	0.87169	0.60178	*2.14*
O	O129	0.73257	0.83378	0.76095	*2.77*
O	O130	0.70092	0.83231	0.48665	*2.44*
**O**	**O131**	0.60543	0.86935	0.60976	***3.11***
O	O132	0.60344	0.84053	0.80172	*1.37*
Si	Si59	0.91193	0.07146	0.85142	*1.00*
Si	Si60	0.78946	0.0307	0.72206	*1.46*
Si	Si61	0.77152	0.04805	0.49058	*2.24*
Si	Si62	0.61232	0.04916	0.49559	*1.38*
Si	Si63	0.552	0.03239	0.71573	*1.99*
Si	Si64	0.6683	0.06915	0.84856	*2.56*
Si	Si65	0.91632	0.82626	0.85757	*0.69*
Si	Si66	0.79797	0.87411	0.72216	*2.64*
Si	Si67	0.78041	0.82778	0.50499	*1.83*
Si	Si68	0.62233	0.82576	0.51093	*2.19*
Si	Si69	0.56113	0.87353	0.7111	*2.80*
Si	Si70	0.67657	0.82624	0.84895	*2.34*
O	O133	0.48448	0.42939	0.71633	*1.49*
O	O134	0.35489	0.43761	0.72601	*1.66*

O	O135	0.41358	0.35829	0.59247	*2.08*
O	O136	0.42109	0.49142	0.57064	*2.54*
O	O137	0.2997	0.4532	0.90028	*2.20*
O	O138	0.281	0.54702	0.75905	*1.63*
O	O139	0.22465	0.42689	0.74468	*0.68*
O	O140	0.30687	0.38277	0.06424	*1.16*
O	O141	0.19574	0.44925	0.02321	*1.89*
O	O142	0.306	0.5154	0.07311	*2.67*
O	O143	0.08728	0.37443	0.05476	*1.31*
O	O144	0.09607	0.4515	0.89593	*2.36*
O	O145	0.09703	0.4356	0.69943	*1.84*
O	O146	0.04424	0.5461	0.77797	*2.01*
O	O147	0.17347	0.35816	0.59256	*2.06*
O	O148	0.49771	0.66486	0.69078	*2.74*
O	O149	0.37337	0.64199	0.741	*2.18*
O	O150	0.30642	0.62976	0.90632	*2.23*
O	O151	0.24352	0.67497	0.74358	*2.30*
O	O152	0.20425	0.66593	0.02151	*1.47*
O	O153	0.10737	0.62537	0.90258	*2.13*
O	O154	0.10265	0.65818	0.71111	*2.08*
Si	Si71	0.41718	0.42997	0.65068	*2.08*
Si	Si72	0.28999	0.46747	0.78224	*1.83*
Si	Si73	0.27599	0.45116	0.01513	*0.28*
Si	Si74	0.11546	0.44567	0.01189	*1.82*
Si	Si75	0.05631	0.46639	0.79296	*2.42*
Si	Si76	0.17108	0.42887	0.65361	*2.36*
Si	Si77	0.42109	0.66868	0.65197	*2.48*
Si	Si78	0.29961	0.62544	0.78571	*2.40*
Si	Si79	0.28361	0.6739	0.00155	*2.24*
Si	Si80	0.12514	0.67046	0.99917	*2.71*
Si	Si81	0.06242	0.62464	0.8005	*2.32*
Al	Al3	0.17941	0.6698	0.65785	*1.74*
**O**	**O155**	0.01145	0.56878	0.21403	***3.08***
O	O156	0.14104	0.563	0.23318	*1.80*
O	O157	0.08622	0.64065	0.09565	*2.16*
O	O158	0.08185	0.5067	0.07489	*2.39*
O	O159	0.20082	0.53933	0.4018	*1.97*
O	O160	0.2149	0.45287	0.25047	*2.47*
O	O161	0.27235	0.57236	0.24503	*2.44*
O	O162	0.20007	0.62336	0.5543	*2.05*
O	O163	0.30331	0.54072	0.527	*2.05*
O	O164	0.18689	0.49104	0.5801	*1.78*
O	O165	0.40908	0.62337	0.55237	*2.38*

O	O166	0.39873	0.54671	0.39354	*2.87*
O	O167	0.39918	0.561	0.19683	*2.13*
O	O168	0.45105	0.45164	0.27728	*2.06*
O	O169	0.32281	0.64628	0.09909	*1.44*
O	O170	0.00347	0.33225	0.19806	*2.04*
O	O171	0.1302	0.34959	0.23858	*2.40*
O	O172	0.19916	0.36952	0.40092	*1.38*
O	O173	0.26193	0.32989	0.23547	*2.20*
O	O174	0.29427	0.3324	0.52611	*2.33*
O	O175	0.3878	0.36912	0.39889	*2.79*
O	O176	0.39297	0.34222	0.20693	*1.43*
Si	Si82	0.08098	0.56918	0.15345	*2.37*
Si	Si83	0.20738	0.53175	0.28343	*2.47*
Si	Si84	0.22295	0.55073	0.51715	*2.22*
Si	Si85	0.38221	0.55122	0.51115	*2.23*
Si	Si86	0.43947	0.53199	0.29196	*2.21*
Si	Si87	0.32522	0.5735	0.15334	*2.36*
Si	Si88	0.07827	0.32643	0.15206	*1.16*
**Si**	**Si89**	0.20099	0.375	0.28131	***3.00***
Si	Si90	0.21554	0.32685	0.50095	*1.36*
Si	Si91	0.37272	0.32635	0.49978	*1.75*
Si	Si92	0.43478	0.3736	0.30044	*2.30*
Si	Si93	0.32153	0.33205	0.15479	*1.43*
O	O177	0.40502	0.74644	0.62261	*1.73*
O	O178	0.30271	0.75216	0.98311	*2.48*
O	O179	0.1015	0.74798	0.98214	*2.34*
O	O180	0.1729	0.75622	0.60049	*1.38*
O	O181	0.09241	0.24935	0.11942	*2.30*
O	O182	0.19353	0.24943	0.48337	*2.31*
O	O183	0.39534	0.2489	0.48316	*2.69*
O	O184	0.32321	0.25505	0.10027	*2.19*
O	O185	0.5939	0.24694	0.38455	*2.69*
O	O186	0.69305	0.24878	0.01733	*2.27*
O	O187	0.89619	0.2475	0.02868	*2.45*
O	O188	0.81932	0.25421	0.40589	*2.06*
O	O189	0.90622	0.74966	0.89696	*2.24*
O	O190	0.80273	0.75028	0.52414	*2.30*
O	O191	0.60139	0.74754	0.52885	*2.86*
O	O192	0.67872	0.74973	0.89138	*2.36*
H	H1	0.34133	0.25531	0.03096	*2.22*
H	H2	0.83758	0.25228	0.47517	*2.10*
H	H3	0.15443	0.75503	0.53145	*1.67*

Table S3. Crystallographic and RMSD data form T12-T11 model with four hexamethylbenzene molecules.

a	19.873
b	20.24
c	13.399

atom	*atom label*	*x*	*y*	*z*	*RMSD$_i$ (Å)*
O	O1	0.4852	0.04867	0.70077	*2.28*
O	O2	0.35234	0.06	0.706	*1.86*
O	O3	0.42468	0.12689	0.57266	*2.41*
O	O4	0.41153	0.9969	0.5556	*2.82*
O	O5	0.28516	0.06407	0.87665	*2.15*
O	O6	0.31302	0.94932	0.79077	*1.84*
O	O7	0.22258	0.03157	0.7132	*1.56*
O	O8	0.30357	0.1301	0.04418	*2.32*
O	O9	0.19837	0.04838	0.02508	*1.71*
O	O10	0.31981	0.99905	0.03951	*2.03*
O	O11	0.09996	0.1371	0.04383	*1.78*
O	O12	0.10637	0.0675	0.88215	*1.52*
O	O13	0.09408	0.05469	0.68696	*1.71*
O	O14	0.07626	0.9514	0.8084	*1.96*
O	O15	0.18221	0.13465	0.60007	*1.17*
O	O16	0.47804	0.86247	0.69283	*2.34*
O	O17	0.34848	0.83354	0.71878	*1.40*
O	O18	0.29344	0.84093	0.89652	*2.12*
O	O19	0.21843	0.86461	0.73703	*1.15*
O	O20	0.19117	0.85165	0.02136	*1.81*
O	O21	0.09029	0.83416	0.89299	*1.35*
O	O22	0.09109	0.84439	0.69652	*2.01*
Si	Si1	0.41778	0.05766	0.63423	*1.85*
Si	Si2	0.29324	0.0257	0.77187	*1.57*
Si	Si3	0.27753	0.06255	0.99752	*2.47*
Si	Si4	0.12052	0.06491	0.00103	*1.23*
Si	Si5	0.0651	0.03005	0.79444	*1.90*
Si	Si6	0.16788	0.05898	0.63423	*2.42*
Si	Si7	0.4116	0.829	0.64251	*2.32*
Si	Si8	0.29257	0.87188	0.7851	*2.27*
Si	Si9	0.26998	0.83003	0.01116	*0.29*
Si	Si10	0.11317	0.82938	0.00866	*2.20*
Si	Si11	0.0588	0.87359	0.79849	*1.85*
Si	Si12	0.16756	0.83608	0.65095	*0.97*
O	O23	0.00315	0.95762	0.20049	*2.09*
O	O24	0.13489	0.94874	0.20761	*1.76*

O	O25	0.06667	0.87754	0.07688	*1.58*
O	O26	0.074	0.00805	0.05268	*1.93*
O	O27	0.1996	0.93998	0.37881	*2.63*
O	O28	0.18303	0.05656	0.29096	*2.34*
O	O29	0.266	0.96652	0.21484	*1.68*
O	O30	0.17441	0.88194	0.55108	*1.73*
O	O31	0.28816	0.95001	0.52802	*2.12*
O	O32	0.1702	0.01251	0.53521	*2.39*
O	O33	0.39297	0.86773	0.53962	*1.96*
O	O34	0.37883	0.94445	0.38221	*2.75*
O	O35	0.39536	0.95348	0.1875	*2.21*
O	O36	0.41712	0.0581	0.30225	*2.73*
O	O37	0.31324	0.87365	0.0896	*1.34*
O	O38	0.01678	0.14574	0.19733	*0.18*
O	O39	0.14577	0.17277	0.22293	*2.34*
O	O40	0.19234	0.16139	0.40522	*1.45*
O	O41	0.27617	0.14925	0.25199	*1.88*
O	O42	0.30136	0.15541	0.51806	*2.36*
O	O43	0.40166	0.17429	0.38985	*2.39*
O	O44	0.40761	0.16683	0.19162	*2.39*
Si	Si13	0.07074	0.94841	0.13416	*2.14*
Si	Si14	0.19607	0.97882	0.27288	*0.24*
Si	Si15	0.20843	0.94549	0.49859	*2.27*
Si	Si16	0.36729	0.93957	0.50159	*1.80*
Si	Si17	0.42376	0.97789	0.29469	*2.48*
Si	Si18	0.32237	0.94802	0.13255	*2.25*
Si	Si19	0.08408	0.1775	0.1451	*1.90*
Si	Si20	0.20194	0.13475	0.2914	*1.61*
Si	Si21	0.22224	0.17638	0.51503	*2.07*
Si	Si22	0.37938	0.17741	0.50682	*2.51*
Si	Si23	0.43732	0.13482	0.30028	*2.35*
Al	Al1	0.31756	0.17823	0.14745	*2.26*
O	O45	0.50885	0.5456	0.29254	*2.08*
O	O46	0.64015	0.55946	0.28493	*2.27*
O	O47	0.5683	0.62976	0.41512	*1.59*
O	O48	0.58441	0.50152	0.44237	*2.42*
O	O49	0.70118	0.57271	0.11102	*2.14*
O	O50	0.68669	0.45357	0.1894	*2.10*
O	O51	0.77044	0.54054	0.27137	*2.13*
O	O52	0.68398	0.63651	0.94062	*1.18*
O	O53	0.79225	0.55977	0.96661	*2.25*
O	O54	0.67219	0.50602	0.94718	*2.44*
O	O55	0.89778	0.63889	0.9522	*2.07*

O	O56	0.88427	0.568	0.11267	*2.24*
O	O57	0.89898	0.5562	0.30646	*2.25*
O	O58	0.91916	0.45277	0.18576	*0.91*
O	O59	0.8122	0.63555	0.39799	*1.46*
O	O60	0.51592	0.36312	0.29563	*2.30*
O	O61	0.64696	0.3432	0.2798	*2.78*
O	O62	0.70545	0.33675	0.10553	*1.41*
O	O63	0.77726	0.37141	0.26306	*2.18*
O	O64	0.80137	0.35077	0.96989	*0.27*
O	O65	0.90151	0.3368	0.10052	*1.10*
O	O66	0.90412	0.34598	0.29777	*2.41*
Si	Si24	0.57571	0.55887	0.35838	*1.77*
Si	Si25	0.69932	0.53105	0.21457	*1.40*
Si	Si26	0.71232	0.56922	0.99146	*0.64*
Si	Si27	0.87148	0.56822	0.99368	*0.83*
Si	Si28	0.92823	0.53165	0.19911	*2.22*
Si	Si29	0.82433	0.5612	0.35622	*2.03*
Si	Si30	0.58209	0.33325	0.35107	*2.59*
Si	Si31	0.70439	0.37638	0.21056	*0.82*
Si	Si32	0.72322	0.32935	0.98816	*0.22*
Si	Si33	0.87995	0.33042	0.98449	*1.64*
Si	Si34	0.93564	0.37469	0.19475	*2.39*
Si	Si35	0.82812	0.33787	0.34496	*1.90*
O	O67	0.99212	0.4604	0.79866	*2.36*
O	O68	0.86167	0.45524	0.78009	*1.71*
O	O69	0.92453	0.37918	0.91443	*0.98*
O	O70	0.91449	0.50867	0.94302	*2.32*
O	O71	0.79276	0.44437	0.61397	*2.87*
O	O72	0.79447	0.55851	0.71188	*1.97*
O	O73	0.72953	0.45506	0.7847	*1.04*
O	O74	0.82151	0.38101	0.44789	*1.67*
O	O75	0.70451	0.44602	0.46435	*2.65*
O	O76	0.81957	0.51179	0.45139	*1.46*
O	O77	0.59457	0.37124	0.4562	*1.97*
O	O78	0.61564	0.44467	0.61387	*2.52*
O	O79	0.59774	0.45001	0.80822	*2.08*
O	O80	0.57928	0.55691	0.69547	*2.03*
O	O81	0.67515	0.37627	0.92126	*1.43*
O	O82	0.97907	0.64554	0.79573	*1.31*
O	O83	0.85055	0.67033	0.77246	*1.96*
O	O84	0.79676	0.66199	0.59306	*2.59*
O	O85	0.71883	0.66337	0.7525	*2.41*
O	O86	0.6911	0.65685	0.47375	*2.12*

O	O87	0.59048	0.67145	0.60171	*2.58*
O	O88	0.59146	0.66671	0.7996	*1.95*
Si	Si36	0.92243	0.45089	0.85939	*2.59*
Si	Si37	0.79454	0.47896	0.72266	*1.35*
Si	Si38	0.78418	0.44512	0.49399	*1.97*
Si	Si39	0.6253	0.44112	0.4942	*2.46*
Si	Si40	0.57022	0.47747	0.70191	*2.34*
Si	Si41	0.66966	0.44699	0.86532	*2.23*
Si	Si42	0.91283	0.67776	0.84932	*0.57*
Si	Si43	0.78979	0.63804	0.70788	*1.75*
Si	Si44	0.77009	0.67703	0.48065	*2.33*
Si	Si45	0.61255	0.67826	0.48598	*2.50*
Si	Si46	0.5593	0.63449	0.69871	*2.88*
Si	Si47	0.66673	0.68101	0.84275	*2.10*
O	O89	0.50218	0.95791	0.30373	*1.25*
O	O90	0.63487	0.9538	0.29439	*1.27*
O	O91	0.56762	0.8811	0.42673	*2.05*
O	O92	0.57351	0.01199	0.44819	*2.01*
O	O93	0.70157	0.93733	0.12282	*2.13*
O	O94	0.68627	0.05597	0.20121	*1.23*
O	O95	0.76648	0.96644	0.28649	*1.44*
O	O96	0.67837	0.87694	0.94987	*1.45*
O	O97	0.78976	0.94751	0.97359	*1.22*
O	O98	0.67143	0.00815	0.96319	*2.47*
O	O99	0.89701	0.86903	0.95758	*0.84*
O	O100	0.88117	0.94203	0.119	*1.28*
O	O101	0.89638	0.95561	0.31449	*1.69*
O	O102	0.91656	0.05788	0.19371	*1.60*
O	O103	0.81586	0.8757	0.4134	*1.64*
O	O104	0.51757	0.14652	0.29984	*2.12*
O	O105	0.64732	0.17029	0.27621	*0.41*
O	O106	0.69416	0.1634	0.09383	*1.60*
O	O107	0.77774	0.14786	0.24677	*2.26*
O	O108	0.80137	0.15736	0.97705	*1.40*
O	O109	0.90122	0.17487	0.10666	*1.96*
O	O110	0.9076	0.16678	0.30545	*1.69*
Si	Si48	0.57059	0.95148	0.36837	*1.83*
Si	Si49	0.69736	0.97901	0.22657	*1.39*
Si	Si50	0.71001	0.94204	0.00276	*2.48*
Si	Si51	0.86921	0.9393	0.99942	*1.23*
Si	Si52	0.92466	0.97803	0.20614	*1.84*
Si	Si53	0.82323	0.95003	0.36912	*2.25*
Si	Si54	0.5848	0.17854	0.35188	*2.39*

Si	Si55	0.70372	0.13428	0.20612	*1.20*
Si	Si56	0.72182	0.1765	0.98232	*1.45*
Si	Si57	0.87952	0.17872	0.98927	*1.16*
Si	Si58	0.93655	0.13478	0.1962	*0.30*
Al	Al2	0.81803	0.17939	0.35035	*1.88*
O	O111	0.98544	0.0473	0.79945	*1.58*
O	O112	0.85286	0.05718	0.79303	*2.00*
O	O113	0.92451	0.12798	0.92341	*2.36*
O	O114	0.91155	0.99885	0.94776	*2.52*
O	O115	0.78574	0.06612	0.62347	*2.22*
O	O116	0.80918	0.94855	0.70483	*2.69*
O	O117	0.72245	0.03123	0.78574	*1.61*
O	O118	0.80388	0.13332	0.45599	*2.72*
O	O119	0.69806	0.05256	0.47559	*1.42*
O	O120	0.8194	0.00206	0.46052	***3.23***
O	O121	0.59911	0.14081	0.45631	*2.55*
O	O122	0.60607	0.07043	0.61897	*2.72*
O	O123	0.59387	0.05125	0.81355	*1.54*
O	O124	0.57518	0.95207	0.68453	*2.08*
O	O125	0.68118	0.13195	0.9014	*1.65*
O	O126	0.9773	0.86405	0.79753	*1.52*
O	O127	0.84748	0.83473	0.78097	*1.68*
O	O128	0.79245	0.83903	0.60264	*2.37*
O	O129	0.71772	0.85934	0.76253	*2.46*
O	O130	0.69191	0.85179	0.47409	*1.89*
O	O131	0.59494	0.83324	0.60749	*1.92*
O	O132	0.58977	0.84837	0.80269	*1.87*
Si	Si59	0.91808	0.05745	0.86541	*2.41*
Si	Si60	0.79272	0.02552	0.72645	*1.65*
Si	Si61	0.77744	0.06578	0.50259	*2.15*
Si	Si62	0.62004	0.06877	0.49985	*1.73*
Si	Si63	0.5646	0.0304	0.70423	*1.41*
Si	Si64	0.66766	0.05637	0.86611	*2.33*
Si	Si65	0.91263	0.83116	0.85305	*2.00*
Si	Si66	0.79099	0.87078	0.71327	*2.50*
Si	Si67	0.77035	0.83057	0.48694	*1.32*
Si	Si68	0.61384	0.83006	0.48992	*1.37*
Si	Si69	0.55876	0.87406	0.69702	*2.48*
Si	Si70	0.66472	0.83537	0.84815	*2.18*
O	O133	0.49199	0.4567	0.69442	*2.12*
O	O134	0.35994	0.44866	0.70413	*2.41*
O	O135	0.42761	0.37727	0.57281	*2.40*
O	O136	0.42021	0.50736	0.5479	*2.10*

O	O137	0.29414	0.43794	0.8744	*2.26*
O	O138	0.31065	0.55543	0.78987	*2.43*
O	O139	0.22849	0.46589	0.71031	*2.44*
O	O140	0.31883	0.38045	0.04722	*2.59*
O	O141	0.20542	0.44891	0.02323	*1.33*
O	O142	0.3233	0.51128	0.03062	*2.41*
O	O143	0.09926	0.3685	0.03654	*2.07*
O	O144	0.11466	0.44288	0.87726	*2.01*
O	O145	0.09847	0.45528	0.68208	*2.33*
O	O146	0.08007	0.55857	0.80177	*1.51*
O	O147	0.17939	0.37501	0.5836	*2.12*
O	O148	0.47827	0.64304	0.6997	*2.12*
O	O149	0.34849	0.67086	0.71884	*1.85*
O	O150	0.30411	0.66065	0.90341	*1.95*
O	O151	0.21895	0.64914	0.75077	*2.60*
O	O152	0.19379	0.65533	0.01397	*2.14*
O	O153	0.09372	0.67643	0.88642	*2.26*
O	O154	0.08852	0.66628	0.68804	*1.64*
Si	Si71	0.42377	0.4482	0.62979	*2.25*
Si	Si72	0.29832	0.47796	0.76933	*2.25*
Si	Si73	0.28534	0.44417	0.9942	*2.46*
Si	Si74	0.12629	0.43953	0.99689	*2.42*
Si	Si75	0.07109	0.47877	0.79044	*1.29*
Si	Si76	0.17186	0.44942	0.62748	*2.06*
Si	Si77	0.41267	0.67583	0.64561	*1.63*
Si	Si78	0.29345	0.63406	0.78975	*2.40*
Si	Si79	0.27325	0.67584	0.01249	*2.12*
Si	Si80	0.11611	0.67822	0.00365	*1.87*
Si	Si81	0.05957	0.63523	0.79738	*1.92*
Al	Al3	0.17885	0.67859	0.64543	*2.18*
O	O155	0.0073	0.55059	0.19306	*2.07*
O	O156	0.14045	0.55859	0.20305	*2.83*
O	O157	0.07041	0.62738	0.06805	*2.46*
O	O158	0.08237	0.49766	0.0497	*1.90*
O	O159	0.20938	0.56578	0.37229	*2.26*
O	O160	0.18128	0.44961	0.29167	*2.46*
O	O161	0.2705	0.53046	0.20815	*2.35*
O	O162	0.19208	0.63185	0.54052	*2.61*
O	O163	0.29705	0.55048	0.52039	*2.57*
O	O164	0.17547	0.50098	0.5354	*2.50*
O	O165	0.39791	0.63691	0.54209	*1.20*
O	O166	0.38956	0.56886	0.37845	*2.66*
O	O167	0.39949	0.55147	0.18334	*1.91*

O	O168	0.41718	0.45071	0.31027	*2.50*
O	O169	0.31235	0.63349	0.09763	*2.64*
O	O170	0.01668	0.36319	0.19336	*1.81*
O	O171	0.14658	0.33505	0.21493	*1.97*
O	O172	0.20124	0.33975	0.39325	*2.48*
O	O173	0.27606	0.36573	0.23406	*2.65*
O	O174	0.30294	0.35113	0.5196	*1.85*
O	O175	0.40263	0.33192	0.38993	*2.83*
O	O176	0.40299	0.34556	0.19362	*2.31*
Si	Si82	0.07605	0.55804	0.12915	*2.81*
Si	Si83	0.20065	0.52581	0.26885	*2.22*
Si	Si84	0.21796	0.56441	0.49318	*2.17*
Si	Si85	0.37541	0.56593	0.49745	*1.90*
Si	Si86	0.42898	0.52896	0.29128	*2.04*
Si	Si87	0.32622	0.55745	0.12995	*2.42*
Si	Si88	0.08268	0.33051	0.14062	*2.13*
Si	Si89	0.20222	0.37239	0.2829	*2.42*
Si	Si90	0.22414	0.33023	0.50842	*2.03*
Si	Si91	0.38096	0.32856	0.50636	*1.63*
Si	Si92	0.43463	0.37304	0.29715	*2.78*
Si	Si93	0.3267	0.33607	0.14844	*2.06*
O	O177	0.42699	0.75241	0.61599	*2.20*
O	O178	0.28068	0.75325	0.04074	*1.10*
O	O179	0.10397	0.75292	0.04436	*1.45*
O	O180	0.18345	0.76153	0.62303	*1.95*
O	O181	0.06856	0.25343	0.11435	*1.01*
O	O182	0.21316	0.25394	0.54119	*0.56*
O	O183	0.39093	0.25276	0.54486	*2.59*
O	O184	0.31139	0.26089	0.12347	*2.20*
O	O185	0.57137	0.25564	0.37675	*2.56*
O	O186	0.71092	0.25357	0.95337	*1.37*
O	O187	0.89193	0.25392	0.95066	*0.21*
O	O188	0.81123	0.26274	0.36785	*1.97*
O	O189	0.92738	0.75401	0.87825	*1.67*
O	O190	0.7813	0.75427	0.4547	*1.95*
O	O191	0.60174	0.75466	0.45154	*2.76*
O	O192	0.67465	0.7578	0.87423	*2.37*
H	H1	0.44107	0.17834	0.13727	*2.33*
H	H2	0.94143	0.17897	0.35876	*1.90*
H	H3	0.05499	0.67812	0.63408	*2.11*

Table S4. Crystallographic and RMSD data for model T7-T8 with three prehnitene (1,2,3,4-TMB) molecules.

a	20.013
b	20.131
c	13.385

atom	*atom label*	*x*	*y*	*z*	*RMSD*$_i$ *(Å)*
O	O1	0.51392	0.04487	0.70485	*1.38*
O	O2	0.40421	0.0437	0.80798	*1.89*
O	O3	0.41809	0.13309	0.66339	*2.34*
O	O4	0.39975	0.00689	0.61804	*2.88*
O	O5	0.33867	0.04146	0.97551	*1.51*
O	O6	0.31917	0.94419	0.84249	*2.28*
O	O7	0.27328	0.0632	0.80369	*1.88*
O	O8	0.3108	0.1146	0.13247	*0.79*
O	O9	0.21334	0.05328	0.02657	*1.37*
O	O10	0.29563	0.98581	0.14333	*2.19*
O	O11	0.12409	0.12393	0.12451	*1.29*
O	O12	0.08634	0.0505	0.96866	*1.77*
O	O13	0.14025	0.06099	0.78875	*1.82*
O	O14	0.11004	0.94281	0.85696	*0.85*
O	O15	0.21063	0.13137	0.66612	*1.66*
O	O16	0.51182	0.85486	0.7242	*2.35*
O	O17	0.39906	0.83954	0.82358	*2.05*
O	O18	0.31995	0.84926	0.98134	*2.03*
O	O19	0.26782	0.82718	0.80222	*1.91*
O	O20	0.19457	0.84156	0.04332	*0.15*
O	O21	0.07624	0.83391	0.95833	*1.45*
O	O22	0.13639	0.82352	0.78235	*1.64*
Si	Si1	0.43426	0.05716	0.69721	*1.68*
Si	Si2	0.33333	0.0229	0.85809	*1.89*
Si	Si3	0.28874	0.04921	0.07039	*0.22*
Si	Si4	0.13601	0.05661	0.0651	*0.36*
Si	Si5	0.08716	0.02039	0.85646	*0.95*
Si	Si6	0.21	0.06237	0.72825	*1.58*
Si	Si7	0.43484	0.83016	0.71522	*2.20*
Si	Si8	0.32644	0.86512	0.86306	*2.02*
Si	Si9	0.27178	0.82307	0.07098	*0.20*
Si	Si10	0.11643	0.82176	0.06199	*2.12*
Si	Si11	0.08378	0.86652	0.84798	*2.70*
Si	Si12	0.20697	0.81651	0.72384	*1.70*
O	O23	0.00344	0.96423	0.21225	*2.71*

O	O24	0.11266	0.93527	0.31133	*2.10*
O	O25	0.08305	0.86606	0.14989	*2.43*
O	O26	0.12404	0.99077	0.13411	*1.89*
O	O27	0.17638	0.94674	0.48138	*1.79*
O	O28	0.1768	0.04645	0.35017	*2.08*
O	O29	0.24675	0.93829	0.31389	*1.43*
O	O30	0.21019	0.86888	0.63222	*2.09*
O	O31	0.30206	0.93978	0.53526	*1.69*
**O**	**O32**	0.21383	0.99942	0.65325	***3.16***
O	O33	0.39528	0.87389	0.63222	*1.60*
O	O34	0.42456	0.93217	0.46085	*2.54*
O	O35	0.3765	0.93001	0.27645	*2.54*
O	O36	0.40135	0.04528	0.36313	*2.58*
O	O37	0.29295	0.85705	0.17555	*1.53*
O	O38	0.99307	0.13797	0.20793	*2.18*
O	O39	0.10699	0.15879	0.32538	*2.22*
O	O40	0.1911	0.14768	0.47366	*1.83*
O	O41	0.23407	0.15651	0.28344	*2.00*
O	O42	0.31298	0.15841	0.54557	*2.28*
O	O43	0.43341	0.15274	0.46859	*2.03*
O	O44	0.36435	0.16347	0.30092	*1.77*
Si	Si13	0.08016	0.93948	0.19976	*1.82*
Si	Si14	0.17875	0.96614	0.36386	*2.44*
Si	Si15	0.22589	0.93843	0.57679	*2.27*
Si	Si16	0.38089	0.93785	0.56246	*1.81*
Si	Si17	0.426	0.96876	0.35212	*2.04*
Si	Si18	0.30274	0.92889	0.22745	*0.13*
Al	Al1	0.07169	0.17146	0.19616	*1.07*
Si	Si19	0.18089	0.12564	0.35953	*2.22*
Si	Si20	0.23476	0.17211	0.56914	*1.50*
Si	Si21	0.39164	0.17426	0.56676	*1.72*
Si	Si22	0.42464	0.12305	0.35561	*2.81*
Si	Si23	0.30349	0.16774	0.22196	*1.05*
O	O45	0.49353	0.53598	0.28914	*2.11*
O	O46	0.59921	0.54407	0.18088	*1.86*
O	O47	0.58649	0.62691	0.33391	*2.32*
O	O48	0.61201	0.50003	0.36626	*1.53*
O	O49	0.66633	0.56222	0.01742	*1.78*
O	O50	0.68526	0.45088	0.12012	*2.32*
O	O51	0.7296	0.56407	0.1933	*2.42*
O	O52	0.70191	0.62478	0.85243	*2.00*
O	O53	0.79129	0.55879	0.96459	*2.00*
O	O54	0.70027	0.494	0.85738	*1.55*

O	O55	0.88111	0.62803	0.86506	*1.07*
O	O56	0.91312	0.57016	0.03808	*0.90*
O	O57	0.86004	0.54422	0.21641	*2.23*
O	O58	0.91407	0.44797	0.10931	*2.00*
O	O59	0.79701	0.63414	0.32278	*2.54*
O	O60	0.49916	0.3687	0.2811	*2.78*
O	O61	0.61344	0.35024	0.1928	*2.29*
O	O62	0.68656	0.33467	0.03172	*2.23*
O	O63	0.74585	0.35021	0.20703	*2.55*
O	O64	0.80943	0.33624	0.95677	*1.29*
O	O65	0.92718	0.3264	0.04402	*2.65*
O	O66	0.87729	0.34854	0.22692	*0.40*
Si	Si24	0.57239	0.55176	0.29434	*2.23*
Si	Si25	0.67052	0.52977	0.12817	*2.20*
Si	Si26	0.71588	0.56032	0.9215	*1.15*
Si	Si27	0.86997	0.56484	0.93463	*1.91*
Si	Si28	0.91898	0.52602	0.13913	*2.19*
Si	Si29	0.79099	0.56128	0.27145	*1.72*
Si	Si30	0.57217	0.33314	0.29494	*2.05*
Si	Si31	0.68235	0.37155	0.13931	*1.51*
Si	Si32	0.73131	0.31988	0.93346	*1.76*
Si	Si33	0.88816	0.31927	0.9381	*2.05*
Si	Si34	0.93072	0.37246	0.14368	*2.40*
Si	Si35	0.80693	0.33433	0.28226	*0.78*
O	O67	0.00608	0.46007	0.79909	*2.07*
O	O68	0.89478	0.44746	0.69724	*2.49*
O	O69	0.91867	0.36806	0.85314	*1.67*
O	O70	0.88941	0.49475	0.88082	*1.47*
O	O71	0.8265	0.46007	0.53058	*2.47*
O	O72	0.8189	0.55491	0.66697	*2.15*
O	O73	0.76215	0.43889	0.70242	*2.14*
O	O74	0.80117	0.37881	0.3827	*2.01*
O	O75	0.70119	0.43989	0.48106	*1.87*
O	O76	0.7778	0.50703	0.35844	*1.64*
O	O77	0.60973	0.36844	0.38977	*2.40*
O	O78	0.57527	0.44839	0.53927	*2.31*
O	O79	0.62973	0.43178	0.71861	*1.75*
O	O80	0.5997	0.55241	0.6591	*2.28*
O	O81	0.70538	0.36286	0.83882	*2.75*
**O**	**O82**	0.01393	0.6308	0.78527	***3.28***
O	O83	0.90101	0.65988	0.66468	*2.34*
O	O84	0.81708	0.64341	0.51675	*2.62*
O	O85	0.77253	0.67573	0.69957	*2.45*

O	O86	0.69395	0.65003	0.44888	*2.11*
O	O87	0.57312	0.64775	0.52761	*2.69*
O	O88	0.64147	0.67458	0.69182	*1.71*
Si	Si36	0.9276	0.44292	0.80907	*2.68*
Si	Si37	0.82534	0.47467	0.64966	*2.79*
Si	Si38	0.77663	0.44614	0.437	*1.96*
Si	Si39	0.624	0.43879	0.44316	*1.42*
Si	Si40	0.57678	0.47492	0.65402	*2.47*
Si	Si41	0.69981	0.43285	0.77958	*2.10*
Al	Al2	0.93811	0.67047	0.79357	*1.73*
Si	Si42	0.82401	0.63188	0.63534	*2.58*
Si	Si43	0.77106	0.6696	0.4237	*2.03*
Si	Si44	0.61537	0.66791	0.42855	*2.30*
Si	Si45	0.58131	0.6305	0.64547	*2.56*
Si	Si46	0.70468	0.68084	0.76698	*1.98*
O	O89	0.50094	0.96537	0.30767	*2.61*
O	O90	0.61264	0.94695	0.21167	*0.89*
O	O91	0.58305	0.87143	0.37	*1.76*
O	O92	0.61803	0.9971	0.39303	*0.69*
O	O93	0.68257	0.95525	0.0475	*2.37*
O	O94	0.69065	0.05268	0.17888	*2.33*
O	O95	0.74442	0.93681	0.22334	*0.99*
O	O96	0.70012	0.86926	0.90382	*1.77*
O	O97	0.80548	0.92841	0.9933	*1.91*
O	O98	0.72864	0.99548	0.87029	*2.70*
O	O99	0.89906	0.86344	0.89579	*1.38*
O	O100	0.93064	0.93361	0.05554	*2.26*
O	O101	0.8769	0.93165	0.23677	*2.25*
O	O102	0.90501	0.04641	0.15185	*2.07*
O	O103	0.804	0.87479	0.37338	*2.61*
O	O104	0.49323	0.13128	0.29666	*1.88*
O	O105	0.60627	0.15575	0.17804	*2.35*
O	O106	0.69195	0.14412	0.03221	*1.99*
O	O107	0.73289	0.17347	0.21844	*2.41*
O	O108	0.81168	0.14861	0.95285	*1.89*
O	O109	0.93028	0.15151	0.03628	*0.28*
O	O110	0.86323	0.16542	0.20464	*0.97*
Si	Si47	0.57852	0.94545	0.32228	*0.75*
Si	Si48	0.68294	0.97239	0.1655	*1.06*
Si	Si49	0.7295	0.93691	0.95293	*1.99*
Si	Si50	0.88304	0.93014	0.95745	*1.99*
Si	Si51	0.9292	0.96994	0.16415	*1.86*
Si	Si52	0.80787	0.93811	0.29907	*2.20*

Al	Al3	0.56916	0.1712	0.30552	*0.67*
Si	Si53	0.68404	0.1299	0.14978	*1.88*
Si	Si54	0.73361	0.16501	0.93229	*1.73*
Si	Si55	0.89023	0.16635	0.93394	*1.40*
Si	Si56	0.92438	0.12539	0.15149	*2.14*
Si	Si57	0.80277	0.18159	0.28054	*2.18*
O	O111	0.01325	0.02883	0.80855	*1.29*
O	O112	0.9052	0.03306	0.70036	*2.40*
O	O113	0.92061	0.12133	0.84436	*0.72*
O	O114	0.89507	0.99621	0.88985	*2.42*
O	O115	0.84086	0.06339	0.53529	*1.59*
O	O116	0.81958	0.94695	0.61897	*2.20*
O	O117	0.77419	0.05572	0.70758	*1.89*
O	O118	0.80501	0.13559	0.37928	*1.54*
O	O119	0.71611	0.06063	0.47871	*1.49*
O	O120	0.80935	0.00638	0.36218	*1.63*
O	O121	0.62417	0.13068	0.38301	*1.73*
O	O122	0.59477	0.06889	0.5537	*1.41*
O	O123	0.64326	0.03282	0.73183	*2.50*
O	O124	0.57953	0.94564	0.61974	*2.35*
O	O125	0.70494	0.12254	0.83953	*2.14*
**O**	**O126**	0.01198	0.8665	0.79292	***3.27***
O	O127	0.90025	0.85523	0.69697	*2.31*
O	O128	0.81159	0.82395	0.55712	*2.77*
O	O129	0.77296	0.85458	0.74192	*1.87*
O	O130	0.69346	0.83968	0.46971	*2.57*
O	O131	0.57701	0.82279	0.55751	*1.99*
O	O132	0.64232	0.85322	0.72583	*2.45*
Si	Si58	0.93404	0.04462	0.81257	*2.37*
Si	Si59	0.83474	0.02481	0.6415	*0.98*
Si	Si60	0.792	0.06661	0.43804	*1.49*
Si	Si61	0.63726	0.06633	0.44939	*2.14*
Si	Si62	0.58344	0.0234	0.65185	*2.11*
Si	Si63	0.71291	0.05078	0.78604	*1.78*
Si	Si64	0.93813	0.83213	0.79967	*2.04*
Si	Si65	0.82645	0.87009	0.65417	*2.57*
Si	Si66	0.77192	0.82271	0.45138	*1.00*
Si	Si67	0.61534	0.82066	0.45001	*2.26*
Si	Si68	0.57803	0.86898	0.65741	*2.53*
Si	Si69	0.70435	0.83237	0.79629	*2.09*
O	O133	0.50209	0.46715	0.69959	*1.46*
O	O134	0.38768	0.43877	0.78745	*1.88*
O	O135	0.41933	0.38109	0.6163	*1.52*

O	O136	0.38552	0.50987	0.62497	*2.30*
O	O137	0.32	0.43008	0.95132	*2.01*
O	O138	0.33511	0.54826	0.87068	*2.51*
O	O139	0.25551	0.46189	0.78274	*2.56*
O	O140	0.28451	0.36144	0.10922	*1.91*
O	O141	0.20577	0.45777	0.03845	*1.84*
O	O142	0.31791	0.4874	0.13191	*2.15*
O	O143	0.12077	0.36686	0.10147	*2.09*
O	O144	0.10244	0.42119	0.9238	*2.45*
O	O145	0.12795	0.4518	0.73309	*2.25*
O	O146	0.09967	0.5453	0.86482	*1.46*
O	O147	0.21563	0.36988	0.65508	*0.53*
O	O148	0.51255	0.64813	0.70312	*2.44*
O	O149	0.39913	0.65265	0.79836	*0.66*
O	O150	0.306	0.66926	0.93399	*2.02*
O	O151	0.27292	0.63232	0.74917	*2.31*
O	O152	0.19507	0.6488	0.03698	*2.42*
O	O153	0.07701	0.66081	0.95231	*2.32*
O	O154	0.14432	0.65582	0.78247	*2.14*
Si	Si70	0.42419	0.44906	0.68026	*1.77*
Si	Si71	0.32401	0.47004	0.84647	*1.97*
Si	Si72	0.28211	0.43406	0.05815	*2.04*
Si	Si73	0.12817	0.43436	0.03739	*0.24*
Si	Si74	0.0834	0.46986	0.83065	*2.44*
Si	Si75	0.2039	0.44546	0.69283	*1.97*
Si	Si76	0.43588	0.67389	0.69512	*2.06*
Si	Si77	0.32768	0.62544	0.83819	*2.31*
Si	Si78	0.27315	0.66984	0.04472	*1.35*
Si	Si79	0.11736	0.66886	0.05641	*0.89*
Si	Si80	0.08248	0.6233	0.84466	*2.42*
Si	Si81	0.20457	0.66409	0.70356	*2.53*
O	O155	0.98861	0.54104	0.19613	*2.18*
O	O156	0.10698	0.52726	0.27669	*2.11*
O	O157	0.08653	0.62154	0.14258	*1.32*
O	O158	0.08455	0.49473	0.08418	*2.13*
O	O159	0.18134	0.55844	0.4316	*2.70*
O	O160	0.20422	0.44542	0.33556	*1.89*
O	O161	0.23378	0.55848	0.247	*2.29*
O	O162	0.18969	0.62466	0.59978	*2.41*
O	O163	0.30005	0.57624	0.5114	*2.26*
O	O164	0.21463	0.49456	0.59828	*2.30*
O	O165	0.3991	0.64059	0.59934	*1.30*
O	O166	0.42492	0.55948	0.45139	*0.74*

O	O167	0.36496	0.56299	0.27573	*2.56*
O	O168	0.40371	0.44753	0.35257	*1.10*
O	O169	0.3124	0.61887	0.11729	*1.63*
O	O170	0.00388	0.36772	0.19403	*1.81*
O	O171	0.11301	0.35191	0.29851	*2.09*
O	O172	0.1835	0.34671	0.46458	*2.43*
O	O173	0.24378	0.32451	0.29278	*2.64*
O	O174	0.30959	0.33827	0.52283	*2.35*
O	O175	0.42616	0.32943	0.43442	*2.37*
O	O176	0.37304	0.33842	0.25249	*1.80*
Si	Si82	0.06784	0.54608	0.17389	*2.68*
Si	Si83	0.18186	0.52249	0.32298	*2.48*
Si	Si84	0.22165	0.56358	0.53659	*2.94*
Si	Si85	0.37713	0.57146	0.54732	*2.32*
Si	Si86	0.42165	0.52595	0.34122	*2.48*
Si	Si87	0.30662	0.55643	0.19284	*0.37*
Si	Si88	0.07757	0.3328	0.19179	*2.15*
Si	Si89	0.18533	0.36717	0.34699	*2.82*
Si	Si90	0.23241	0.32644	0.55658	*2.37*
Si	Si91	0.38892	0.32558	0.54176	*2.63*
Si	Si92	0.42548	0.37106	0.33027	*2.54*
Si	Si93	0.30093	0.31753	0.20735	*2.49*
O	O177	0.43267	0.75341	0.6802	*2.59*
O	O178	0.28034	0.74411	0.08877	*2.26*
O	O179	0.1104	0.74472	0.09617	*1.46*
O	O180	0.21369	0.7423	0.67802	*2.50*
O	O181	0.07405	0.25495	0.17531	*1.74*
O	O182	0.2203	0.24986	0.58893	*0.80*
O	O183	0.40174	0.25288	0.5912	*1.78*
O	O184	0.30254	0.24076	0.17186	*1.99*
O	O185	0.56629	0.2557	0.31463	*1.65*
O	O186	0.72214	0.24221	0.90462	*0.48*
O	O187	0.89778	0.24339	0.89914	*1.95*
O	O188	0.80572	0.2577	0.31792	*2.36*
O	O189	0.94168	0.75398	0.81134	*1.83*
O	O190	0.77985	0.74843	0.4061	*2.33*
O	O191	0.60794	0.74641	0.40413	*2.52*
O	O192	0.70372	0.7533	0.81843	*2.61*
H	H1	0.07306	0.16881	0.37947	*2.33*
H	H2	0.57491	0.1682	0.12183	*2.30*
H	H3	0.93181	0.67436	0.60933	*2.15*

Table S5. Crystallographic and RMSD data for model T8-T7 with three naphthalene molecules.

a	19.956
b	20.025
c	13.334

atom	*atom label*	*x*	*y*	*z*	*RMSD$_i$ (Å)*
O	O1	0.49969	0.03724	0.704	*1.88*
O	O2	0.38663	0.05043	0.80236	*1.42*
O	O3	0.41426	0.12934	0.64661	*2.14*
**O**	**O4**	0.38504	0.00035	0.61923	***3.05***
O	O5	0.31411	0.03458	0.96616	*1.85*
O	O6	0.30597	0.94514	0.81989	*2.16*
O	O7	0.25437	0.06504	0.79452	*1.94*
O	O8	0.30241	0.12307	0.10732	*2.04*
O	O9	0.19153	0.07208	0.02041	*1.87*
O	O10	0.262	0.99892	0.14265	*2.57*
O	O11	0.0929	0.13313	0.11544	*1.91*
O	O12	0.06696	0.05928	0.95577	*1.99*
O	O13	0.12189	0.06475	0.77414	*0.84*
O	O14	0.09344	0.94755	0.85361	*0.80*
O	O15	0.19444	0.14014	0.65958	*1.98*
O	O16	0.5057	0.86239	0.71162	*2.19*
O	O17	0.39501	0.84551	0.81861	*2.10*
O	O18	0.31323	0.85874	0.9722	*2.05*
O	O19	0.2647	0.82253	0.79479	*1.52*
O	O20	0.19105	0.85228	0.04947	*2.13*
O	O21	0.06697	0.84612	0.9767	*0.94*
O	O22	0.13192	0.82457	0.80912	*1.28*
Si	Si1	0.42111	0.05459	0.69184	*1.87*
Si	Si2	0.31526	0.0241	0.84586	*1.88*
Si	Si3	0.26758	0.0574	0.05981	*1.82*
Si	Si4	0.11397	0.0656	0.05497	*1.86*
Si	Si5	0.06961	0.02511	0.84483	*0.09*
Si	Si6	0.19295	0.06871	0.71505	*1.92*
Si	Si7	0.43101	0.83034	0.711	*2.07*
Si	Si8	0.32003	0.86814	0.85234	*2.32*
Si	Si9	0.27023	0.83511	0.06917	*2.00*
Si	Si10	0.11371	0.83117	0.07443	*2.20*
Si	Si11	0.07344	0.86901	0.86037	*2.32*
Si	Si12	0.19496	0.81985	0.73257	*1.28*
O	O23	0.98607	0.96293	0.20213	*1.75*
O	O24	0.09191	0.96084	0.31563	*2.34*

O	O25	0.08347	0.87335	0.16747	*2.03*
O	O26	0.10331	0.00085	0.1258	*2.76*
O	O27	0.16162	0.93658	0.47814	*0.44*
O	O28	0.18553	0.04746	0.37305	*2.77*
O	O29	0.22079	0.9292	0.30034	*2.05*
O	O30	0.18835	0.87763	0.64934	*1.96*
O	O31	0.28658	0.93098	0.54028	*2.15*
O	O32	0.19858	0.00865	0.63454	*2.05*
O	O33	0.38636	0.86499	0.62308	*2.11*
O	O34	0.40599	0.92947	0.45475	*2.23*
O	O35	0.34844	0.9678	0.28314	*2.53*
O	O36	0.42378	0.05135	0.3854	*2.40*
O	O37	0.29886	0.87351	0.1667	*1.53*
**O**	**O38**	0.97915	0.14876	0.2195	***3.45***
O	O39	0.0892	0.14988	0.31609	*2.06*
O	O40	0.18361	0.17538	0.46548	*1.79*
O	O41	0.2199	0.16248	0.25357	*1.59*
O	O42	0.30287	0.16057	0.55032	*2.16*
O	O43	0.41844	0.17315	0.45719	*1.83*
O	O44	0.34791	0.14105	0.29251	*2.22*
Si	Si13	0.06597	0.94921	0.20215	*1.23*
Si	Si14	0.16574	0.97046	0.367	*2.01*
Si	Si15	0.20935	0.9387	0.57611	*2.16*
Si	Si16	0.36638	0.93168	0.56094	*1.92*
Si	Si17	0.41371	0.97353	0.35319	*1.30*
Si	Si18	0.28252	0.94284	0.22449	*1.65*
Si	Si19	0.05556	0.17246	0.20401	*1.84*
Al	Al1	0.17749	0.13169	0.35603	*2.72*
Si	Si20	0.22479	0.18331	0.56684	*1.72*
Si	Si21	0.38106	0.17949	0.56543	*2.33*
Si	Si22	0.41556	0.12926	0.35525	*2.34*
Si	Si23	0.29403	0.1674	0.20899	*2.26*
O	O45	0.50622	0.5291	0.28569	*2.13*
O	O46	0.61848	0.56489	0.19439	*0.92*
O	O47	0.58454	0.61932	0.36927	*2.04*
O	O48	0.62558	0.4917	0.3553	*1.73*
O	O49	0.68546	0.56594	0.02623	*2.27*
O	O50	0.66514	0.451	0.11757	*2.27*
O	O51	0.75037	0.53871	0.19514	*1.57*
O	O52	0.7279	0.64457	0.88269	*2.10*
O	O53	0.80613	0.54669	0.95023	*1.41*
O	O54	0.69891	0.51833	0.84132	*1.45*
O	O55	0.89949	0.62363	0.86908	*0.33*

O	O56	0.9168	0.57427	0.05327	*1.66*
O	O57	0.87675	0.55705	0.24279	*1.34*
O	O58	0.90853	0.45229	0.13012	*1.87*
O	O59	0.78644	0.64019	0.31425	*1.46*
O	O60	0.49694	0.35763	0.29651	*2.01*
O	O61	0.60997	0.34242	0.19682	*2.62*
O	O62	0.69773	0.33088	0.05007	*1.85*
O	O63	0.73757	0.37074	0.23084	*1.81*
O	O64	0.8139	0.3466	0.95622	*0.66*
O	O65	0.9353	0.33992	0.0321	*2.42*
O	O66	0.86572	0.33684	0.20151	*2.20*
Si	Si24	0.58346	0.55202	0.30262	*2.19*
Si	Si25	0.68031	0.52993	0.13443	*1.86*
Si	Si26	0.72951	0.56919	0.92421	*2.05*
Si	Si27	0.8857	0.55979	0.94242	*0.33*
Si	Si28	0.92553	0.52979	0.15522	*0.53*
Si	Si29	0.80025	0.56319	0.28369	*0.15*
Si	Si30	0.57267	0.32816	0.30254	*1.75*
Si	Si31	0.67785	0.3737	0.1483	*2.46*
Si	Si32	0.73425	0.33073	0.94127	*0.13*
Si	Si33	0.8909	0.3258	0.9313	*1.36*
Si	Si34	0.92605	0.37355	0.14208	*2.49*
Si	Si35	0.80479	0.34095	0.2823	*2.52*
O	O67	0.0207	0.44951	0.78624	*1.67*
O	O68	0.90359	0.46674	0.70479	*2.24*
O	O69	0.92062	0.36851	0.83817	*0.18*
O	O70	0.92214	0.4937	0.89931	*2.05*
O	O71	0.83287	0.45144	0.54479	*2.81*
O	O72	0.8104	0.55773	0.66137	*2.62*
O	O73	0.7748	0.43805	0.72521	*1.49*
O	O74	0.82467	0.39081	0.37324	*0.75*
O	O75	0.71265	0.42955	0.4726	*1.60*
O	O76	0.79089	0.51824	0.38379	*1.76*
O	O77	0.61255	0.36181	0.39551	*2.03*
O	O78	0.58705	0.4511	0.53476	*1.27*
O	O79	0.64103	0.43672	0.71432	*2.01*
O	O80	0.60443	0.55693	0.65196	*2.28*
O	O81	0.70151	0.38746	0.87093	*1.50*
O	O82	0.01807	0.65234	0.78307	*2.51*
O	O83	0.91196	0.65741	0.67454	*2.04*
O	O84	0.82444	0.65946	0.50626	*1.26*
O	O85	0.78023	0.69195	0.71075	*2.51*
O	O86	0.69662	0.65934	0.45906	*2.42*

O	O87	0.58131	0.66876	0.55414	*1.46*
O	O88	0.64929	0.67012	0.72676	*2.62*
Si	Si36	0.9405	0.44406	0.80659	*2.12*
Si	Si37	0.82902	0.48035	0.65858	*2.27*
Si	Si38	0.78977	0.44752	0.44293	*1.65*
Si	Si39	0.63477	0.43396	0.43984	*1.32*
Si	Si40	0.5866	0.47826	0.64951	*2.75*
Si	Si41	0.70544	0.44579	0.78758	*1.19*
Si	Si42	0.93966	0.67112	0.79298	*1.91*
Al	Al2	0.82363	0.64007	0.63149	*2.52*
Si	Si43	0.7722	0.67888	0.42019	*1.54*
Si	Si44	0.61714	0.67345	0.4452	*2.02*
Si	Si45	0.58953	0.63614	0.66494	*1.41*
Si	Si46	0.71729	0.69222	0.78507	*1.44*
O	O89	0.47828	0.94726	0.29107	*2.28*
O	O90	0.5948	0.96548	0.20667	*2.39*
O	O91	0.57996	0.86863	0.34236	*2.78*
O	O92	0.57804	0.99451	0.40034	*2.65*
O	O93	0.66752	0.95008	0.04858	*2.01*
O	O94	0.68745	0.05763	0.16318	*1.95*
O	O95	0.72367	0.9391	0.23073	*2.06*
O	O96	0.67571	0.89055	0.87612	*2.20*
**O**	**O97**	0.78758	0.92932	0.9759	***3.21***
O	O98	0.70918	0.0182	0.88825	*2.71*
O	O99	0.88677	0.86069	0.89738	*1.55*
O	O100	0.91339	0.94722	0.03923	*0.95*
O	O101	0.85727	0.93633	0.21815	*1.74*
O	O102	0.89612	0.05484	0.15163	*2.25*
O	O103	0.79724	0.88742	0.37545	*1.93*
O	O104	0.47938	0.15264	0.28668	*1.78*
O	O105	0.58609	0.15744	0.17745	*2.04*
O	O106	0.67501	0.16069	0.00896	*1.46*
O	O107	0.71874	0.19125	0.21302	*2.45*
O	O108	0.80244	0.16061	0.95886	*1.50*
O	O109	0.91772	0.16732	0.05131	*1.64*
O	O110	0.85002	0.16837	0.22535	*2.34*
Si	Si47	0.55867	0.94356	0.30967	*1.60*
Si	Si48	0.66951	0.98001	0.1618	*1.29*
Si	Si49	0.71027	0.94701	0.94633	*0.95*
Si	Si50	0.86544	0.93262	0.94339	*1.52*
Si	Si51	0.91311	0.97565	0.15317	*1.26*
Si	Si52	0.7933	0.94586	0.2923	*1.99*
Si	Si53	0.5578	0.17149	0.29575	*2.33*

Al	Al3	0.67415	0.14006	0.1338	*1.99*
Si	Si54	0.72629	0.17946	0.92138	*1.78*
Si	Si55	0.88162	0.17382	0.94248	*1.82*
Si	Si56	0.90864	0.13435	0.16122	*2.21*
Si	Si57	0.7825	0.1917	0.28521	*2.58*
O	O111	0.99592	0.02876	0.7942	*1.67*
O	O112	0.88519	0.05977	0.69462	*1.58*
O	O113	0.91395	0.12006	0.86613	*1.80*
O	O114	0.87593	0.99139	0.86076	*1.38*
O	O115	0.81409	0.06734	0.53016	*2.08*
O	O116	0.83295	0.94989	0.61462	*2.48*
O	O117	0.75253	0.03834	0.70125	*2.34*
O	O118	0.77414	0.14517	0.38441	*2.26*
O	O119	0.69334	0.04919	0.45246	*2.46*
O	O120	0.80136	0.01831	0.34623	*1.22*
O	O121	0.59871	0.12486	0.3724	*2.33*
O	O122	0.58256	0.07392	0.55605	*2.09*
O	O123	0.62487	0.05476	0.74379	*1.03*
O	O124	0.58887	0.95152	0.63138	*2.56*
O	O125	0.7121	0.13979	0.81652	*1.94*
**O**	**O126**	0.00341	0.85615	0.80162	***3.20***
O	O127	0.89103	0.84434	0.69826	*1.77*
O	O128	0.80229	0.82847	0.55297	*1.39*
O	O129	0.76329	0.87104	0.73392	*1.83*
O	O130	0.68546	0.84582	0.46172	*2.45*
O	O131	0.56376	0.8383	0.53605	*1.70*
O	O132	0.63584	0.83745	0.70268	*2.50*
Si	Si58	0.91787	0.05052	0.80516	*2.08*
Si	Si59	0.82056	0.02874	0.63636	*0.47*
Si	Si60	0.77038	0.07014	0.42766	*2.23*
Si	Si61	0.61334	0.0607	0.44478	*2.61*
Si	Si62	0.57408	0.02914	0.65782	*2.49*
Si	Si63	0.70032	0.06227	0.78709	*2.12*
Si	Si64	0.92702	0.82787	0.80423	*2.23*
Si	Si65	0.82186	0.87306	0.64969	*2.60*
Si	Si66	0.76477	0.82982	0.44477	*2.37*
Si	Si67	0.60848	0.82512	0.4354	*2.62*
Si	Si68	0.57355	0.87243	0.64574	*1.35*
Si	Si69	0.69567	0.84094	0.7849	*2.43*
O	O133	0.51283	0.46593	0.69582	*0.92*
O	O134	0.40497	0.46882	0.8045	*2.31*
O	O135	0.41531	0.37761	0.66212	*2.04*
O	O136	0.39666	0.50418	0.6135	*2.65*

O	O137	0.33815	0.44146	0.96771	*2.32*
O	O138	0.31258	0.55454	0.86941	*2.24*
O	O139	0.27468	0.4382	0.79383	*1.74*
O	O140	0.31836	0.37037	0.13097	*1.25*
O	O141	0.21548	0.42723	0.03644	*0.24*
O	O142	0.30367	0.50107	0.13526	*2.01*
O	O143	0.1114	0.36148	0.10739	*1.61*
O	O144	0.10049	0.42912	0.94035	*2.30*
O	O145	0.14797	0.47555	0.76524	*2.70*
O	O146	0.07628	0.5528	0.88278	*1.92*
O	O147	0.20088	0.38264	0.65198	*1.79*
O	O148	0.51982	0.64907	0.72485	*2.45*
O	O149	0.40259	0.64492	0.80608	*1.06*
O	O150	0.31383	0.6754	0.94589	*2.61*
O	O151	0.27761	0.65826	0.75519	*2.52*
O	O152	0.19788	0.65811	0.03943	*2.08*
O	O153	0.08215	0.67561	0.95047	*2.18*
O	O154	0.14857	0.64159	0.78202	*2.25*
Si	Si70	0.43299	0.45402	0.69253	*1.81*
Si	Si71	0.33264	0.47596	0.8583	*1.65*
Si	Si72	0.29336	0.43485	0.06855	*1.16*
Si	Si73	0.13521	0.42892	0.0503	*1.52*
Si	Si74	0.08671	0.47631	0.8439	*0.79*
Si	Si75	0.21614	0.45089	0.71171	*1.30*
Si	Si76	0.44263	0.67095	0.70698	*2.39*
Si	Si77	0.32665	0.63275	0.84463	*1.70*
Si	Si78	0.27589	0.68019	0.05319	*1.45*
Si	Si79	0.11994	0.67796	0.05885	*1.54*
Si	Si80	0.08277	0.63044	0.84977	*2.18*
Si	Si81	0.20476	0.66962	0.70472	*2.43*
O	O155	0.00109	0.53549	0.19823	*1.59*
O	O156	0.11511	0.54745	0.29329	*1.79*
O	O157	0.08737	0.62535	0.1365	*0.28*
O	O158	0.11443	0.49607	0.1108	*1.43*
**O**	**O159**	0.18885	0.54257	0.4563	***3.23***
O	O160	0.20066	0.44692	0.32031	*1.98*
O	O161	0.24612	0.56883	0.28058	*2.23*
O	O162	0.20098	0.63069	0.59842	*2.18*
O	O163	0.31164	0.57978	0.51048	*2.47*
O	O164	0.24062	0.50762	0.63384	*2.32*
O	O165	0.41326	0.63639	0.60578	*1.65*
O	O166	0.43576	0.56242	0.44573	*2.53*
O	O167	0.37971	0.5659	0.26602	*2.09*

O	O168	0.40771	0.44945	0.34795	*2.52*
O	O169	0.31124	0.63298	0.13572	*1.76*
**O**	**O170**	0.99533	0.3644	0.20516	***3.28***
O	O171	0.10842	0.35039	0.30593	*2.75*
O	O172	0.19016	0.3517	0.46056	*2.05*
O	O173	0.23666	0.32534	0.27678	*2.65*
O	O174	0.3102	0.35391	0.54255	*1.22*
O	O175	0.43374	0.34747	0.47139	*2.60*
O	O176	0.36824	0.327	0.30327	*1.67*
Si	Si82	0.07981	0.5512	0.18353	*2.28*
Si	Si83	0.18808	0.52635	0.33729	*2.14*
Si	Si84	0.23583	0.56518	0.54927	*2.90*
Si	Si85	0.38945	0.57081	0.54497	*2.23*
Si	Si86	0.43251	0.52658	0.33637	*2.39*
Si	Si87	0.30993	0.56661	0.20468	*1.74*
Si	Si88	0.06982	0.33192	0.2023	*2.01*
Si	Si89	0.18409	0.36866	0.34264	*1.18*
Si	Si90	0.2313	0.33651	0.56306	*2.63*
Si	Si91	0.38726	0.33324	0.56971	*2.13*
Si	Si92	0.42692	0.37072	0.35512	*2.22*
Si	Si93	0.30864	0.31755	0.22171	*2.52*
O	O177	0.43469	0.75073	0.69252	*2.22*
O	O178	0.28011	0.75642	0.09147	*1.97*
O	O179	0.11099	0.75246	0.10423	*1.91*
O	O180	0.1913	0.74736	0.67883	*1.98*
O	O181	0.06422	0.2514	0.18941	*1.82*
O	O182	0.22299	0.26098	0.60253	*2.05*
O	O183	0.39157	0.25565	0.60407	*1.78*
O	O184	0.31409	0.24314	0.17593	*1.65*
O	O185	0.56977	0.24832	0.32381	*2.50*
O	O186	0.72388	0.25823	0.89034	*1.47*
O	O187	0.89663	0.24795	0.89895	*1.63*
O	O188	0.7943	0.26703	0.32783	*2.37*
O	O189	0.92828	0.74765	0.82273	*2.23*
O	O190	0.77459	0.75803	0.39117	*1.78*
O	O191	0.60341	0.74764	0.40065	*1.87*
O	O192	0.70738	0.76717	0.83006	*2.34*
H	H1	0.55043	0.16114	0.12427	*2.31*
H	H2	0.05509	0.15023	0.37135	*2.08*
H	H3	0.94768	0.66119	0.62181	*2.05*

Table S6. Crystallographic and RMSD data for silicalite model with four hexamethylbenzene molecules.

a	20.02
b	20.044
c	13.405

atom	*atom label*	*x*	*y*	*z*	*RMSD$_i$ (Å)*
O	O1	0.51441	0.0433	0.70155	*2.28*
O	O2	0.40749	0.04703	0.81123	*1.36*
O	O3	0.41776	0.13221	0.6613	*1.84*
O	O4	0.39858	0.00494	0.62374	*2.73*
O	O5	0.3286	0.07677	0.96223	*2.31*
O	O6	0.33594	0.95352	0.90193	*2.29*
O	O7	0.27825	0.04287	0.78224	*2.27*
O	O8	0.30371	0.14096	0.12909	*2.28*
O	O9	0.20846	0.07177	0.03296	*2.03*
O	O10	0.30476	0.00783	0.12945	*2.49*
O	O11	0.11573	0.14452	0.11895	*1.94*
O	O12	0.08433	0.06676	0.96733	*1.47*
O	O13	0.14751	0.0467	0.79584	*1.72*
O	O14	0.08746	0.94689	0.88628	*1.86*
O	O15	0.20969	0.12299	0.66408	*2.57*
O	O16	0.50736	0.86268	0.71854	*1.41*
O	O17	0.3959	0.84929	0.81837	*2.04*
O	O18	0.31731	0.83367	0.97563	*2.64*
O	O19	0.26541	0.86776	0.79918	*2.13*
O	O20	0.19309	0.84286	0.04074	*2.24*
O	O21	0.07813	0.82416	0.94793	*1.30*
O	O22	0.13671	0.84934	0.77362	*1.05*
Si	Si1	0.4349	0.05722	0.6984	*1.67*
Si	Si2	0.33657	0.03032	0.86393	*1.91*
Si	Si3	0.28648	0.07414	0.06557	*2.06*
Si	Si4	0.13095	0.07376	0.06554	*1.99*
Si	Si5	0.08367	0.02605	0.86273	*1.60*
Si	Si6	0.20988	0.05102	0.71933	*2.30*
Si	Si7	0.4313	0.835	0.71167	*1.67*
Si	Si8	0.32874	0.87535	0.87364	*2.60*
Si	Si9	0.26995	0.82616	0.07337	*2.02*
Si	Si10	0.11394	0.82727	0.05684	*2.13*
Si	Si11	0.07762	0.87059	0.84878	*1.00*
Si	Si12	0.20982	0.83192	0.72991	*1.72*
O	O23	0.99804	0.97243	0.20705	*1.02*
O	O24	0.10878	0.93814	0.30036	*2.50*

O	O25	0.07986	0.88446	0.12461	*2.36*
O	O26	0.11665	0.01227	0.14116	*2.46*
O	O27	0.17972	0.93208	0.46254	*2.55*
O	O28	0.15765	0.04921	0.38103	*2.10*
O	O29	0.23876	0.96379	0.28895	*2.04*
O	O30	0.21601	0.86083	0.61715	*1.91*
O	O31	0.30175	0.94431	0.52735	*2.03*
**O**	O32	0.20236	0.99197	0.6374	***3.22***
O	O33	0.39229	0.87234	0.62121	*1.82*
O	O34	0.42425	0.93934	0.45665	*1.93*
O	O35	0.36922	0.94869	0.27631	*2.84*
O	O36	0.40781	0.05735	0.3696	*2.29*
O	O37	0.29196	0.87715	0.16119	*1.61*
**O**	O38	0.99684	0.14539	0.20599	***3.25***
O	O39	0.10887	0.16332	0.31437	*1.98*
O	O40	0.18678	0.16171	0.47542	*2.86*
O	O41	0.23934	0.13436	0.29845	*2.54*
O	O42	0.30988	0.15999	0.54785	*1.72*
O	O43	0.43128	0.16721	0.47146	*1.44*
O	O44	0.36688	0.17275	0.29828	*2.10*
Si	Si13	0.07536	0.95126	0.19182	*2.38*
Si	Si14	0.17241	0.97121	0.35721	*2.12*
Si	Si15	0.22506	0.93251	0.56251	*2.09*
Si	Si16	0.3798	0.93999	0.55803	*1.33*
Si	Si17	0.42463	0.97925	0.3507	*2.58*
Si	Si18	0.30032	0.94978	0.21349	*2.42*
Si	Si19	0.07165	0.17723	0.20827	*1.16*
Si	Si20	0.17301	0.12807	0.36752	*2.36*
Si	Si21	0.23132	0.17331	0.57518	*1.54*
Si	Si22	0.38769	0.17794	0.57265	*1.57*
Si	Si23	0.42601	0.13586	0.36015	*2.71*
Si	Si24	0.29644	0.17513	0.23852	*2.19*
O	O45	0.48219	0.5432	0.30772	*1.97*
O	O46	0.58888	0.54745	0.19726	*0.42*
O	O47	0.57881	0.63213	0.3478	*2.28*
O	O48	0.59852	0.50476	0.3841	*0.68*
O	O49	0.66767	0.57665	0.04603	*1.35*
O	O50	0.66034	0.45354	0.10711	*2.51*
O	O51	0.71826	0.54326	0.2259	*2.54*
O	O52	0.6932	0.64076	0.8795	*1.87*
O	O53	0.78787	0.57066	0.97548	*2.14*
O	O54	0.69101	0.50765	0.87872	*2.10*
O	O55	0.88012	0.64435	0.88994	*2.07*

O	O56	0.91195	0.56674	0.0416	*2.07*
O	O57	0.8491	0.54617	0.21325	*2.07*
O	O58	0.90923	0.4467	0.12226	*1.99*
O	O59	0.7869	0.62287	0.34457	*1.80*
O	O60	0.48888	0.36271	0.2905	*2.36*
O	O61	0.60004	0.34934	0.19003	*1.48*
O	O62	0.67925	0.3338	0.03329	*2.66*
O	O63	0.7304	0.36758	0.21036	*2.73*
O	O64	0.80349	0.34292	0.96806	*1.96*
O	O65	0.91844	0.32402	0.06082	*2.56*
O	O66	0.85919	0.34934	0.23464	*2.80*
Si	Si25	0.56169	0.55724	0.3103	*1.44*
Si	Si26	0.65976	0.53046	0.1446	*2.23*
Si	Si27	0.70988	0.57373	0.94279	*2.20*
Si	Si28	0.86544	0.57342	0.94321	*1.93*
Si	Si29	0.9129	0.52584	0.14603	*1.51*
Si	Si30	0.78648	0.55092	0.28925	*2.15*
Si	Si31	0.56492	0.33494	0.29693	*1.55*
Si	Si32	0.66743	0.37535	0.13528	*2.36*
Si	Si33	0.72662	0.32614	0.93557	*1.27*
Si	Si34	0.88263	0.32721	0.95191	*2.23*
Si	Si35	0.91864	0.37042	0.16003	*2.69*
Si	Si36	0.78631	0.33177	0.27908	*2.58*
O	O67	0.99876	0.47194	0.8011	*2.93*
O	O68	0.8879	0.43798	0.70815	*2.39*
O	O69	0.91678	0.38427	0.88398	*2.05*
O	O70	0.88043	0.51217	0.8675	*1.94*
O	O71	0.8168	0.43239	0.54613	*2.53*
O	O72	0.8391	0.54937	0.62831	*2.15*
O	O73	0.75808	0.46382	0.72019	*2.49*
O	O74	0.78052	0.36067	0.39191	*2.31*
O	O75	0.69455	0.44381	0.48194	*2.38*
O	O76	0.79341	0.49182	0.37118	*2.79*
O	O77	0.60409	0.37216	0.38737	*2.11*
O	O78	0.57174	0.43979	0.55139	*1.93*
O	O79	0.62776	0.44791	0.73104	*1.77*
O	O80	0.58878	0.55734	0.64006	*2.57*
O	O81	0.70465	0.37697	0.8475	*1.98*
**O**	O82	0.99916	0.64529	0.80329	***3.36***
O	O83	0.88714	0.66385	0.69482	*2.14*
O	O84	0.81002	0.66161	0.53305	*2.80*
O	O85	0.75656	0.63443	0.70925	*2.58*
O	O86	0.68675	0.65983	0.46107	*2.47*

O	O87	0.56531	0.66696	0.53762	*1.35*
O	O88	0.62899	0.67296	0.71141	*1.90*
Si	Si37	0.92141	0.45102	0.81667	*2.20*
Si	Si38	0.82427	0.47129	0.65162	*2.07*
Si	Si39	0.77123	0.43238	0.4464	*2.87*
Si	Si40	0.61662	0.43987	0.45042	*0.25*
Si	Si41	0.57199	0.47911	0.65786	*2.19*
Si	Si42	0.6962	0.44949	0.7949	*1.67*
Si	Si43	0.92441	0.67731	0.80103	*1.50*
Si	Si44	0.82337	0.62821	0.64122	*1.71*
Si	Si45	0.76528	0.67319	0.43348	*2.14*
Si	Si46	0.6089	0.67781	0.43649	*2.22*
Si	Si47	0.57021	0.63581	0.64909	*2.71*
Si	Si48	0.69984	0.67511	0.77012	*2.55*
O	O89	0.49814	0.97221	0.30147	*2.14*
O	O90	0.60891	0.93766	0.20818	*1.02*
O	O91	0.57986	0.88422	0.38408	*2.04*
O	O92	0.61678	0.01198	0.36718	*2.53*
O	O93	0.67964	0.93235	0.04563	*2.33*
O	O94	0.65714	0.04915	0.12813	*1.60*
O	O95	0.73885	0.9638	0.21909	*1.41*
O	O96	0.71626	0.86101	0.89126	*1.19*
O	O97	0.80174	0.94472	0.98094	*1.73*
O	O98	0.70225	0.99213	0.87076	*2.93*
O	O99	0.89233	0.87256	0.88732	*1.68*
O	O100	0.92417	0.93974	0.0518	*1.37*
O	O101	0.8692	0.94841	0.23228	*0.89*
O	O102	0.90741	0.05746	0.13977	*2.03*
O	O103	0.79159	0.87679	0.3468	*1.81*
O	O104	0.49689	0.14535	0.30272	*1.50*
O	O105	0.60894	0.16367	0.19416	*2.29*
O	O106	0.68646	0.16125	0.03269	*2.32*
O	O107	0.7394	0.13433	0.2094	*2.45*
O	O108	0.80978	0.15984	0.96094	*2.08*
O	O109	0.93117	0.16705	0.03735	*2.22*
O	O110	0.86688	0.17306	0.21065	*1.70*
Si	Si49	0.57545	0.95094	0.31668	*1.79*
Si	Si50	0.6723	0.97113	0.15124	*2.05*
Si	Si51	0.72507	0.93277	0.94576	*1.83*
Si	Si52	0.87981	0.94028	0.95036	*1.13*
Si	Si53	0.92448	0.97936	0.158	*1.48*
Si	Si54	0.80019	0.9495	0.29481	*1.82*
Si	Si55	0.57171	0.17724	0.30038	*2.07*

Si	Si56	0.67283	0.12801	0.1409	*2.57*
Si	Si57	0.73127	0.17322	0.93333	*0.92*
Si	Si58	0.88758	0.17795	0.93625	*1.13*
Si	Si59	0.92588	0.13594	0.14882	*1.66*
Si	Si60	0.79625	0.17502	0.26994	*2.33*
O	O111	0.01444	0.04376	0.80691	*1.03*
O	O112	0.90754	0.04748	0.69713	*1.17*
O	O113	0.91767	0.13246	0.84733	*1.51*
O	O114	0.89862	0.00512	0.88446	*2.63*
O	O115	0.82859	0.07656	0.54606	*2.57*
O	O116	0.83624	0.95344	0.60712	*2.46*
O	O117	0.77827	0.0429	0.7261	*1.16*
O	O118	0.80325	0.14048	0.37915	*2.62*
O	O119	0.7083	0.07139	0.47597	*2.68*
**O**	O120	0.80447	0.00734	0.3792	***3.25***
O	O121	0.61591	0.14424	0.38935	*1.85*
O	O122	0.58398	0.06649	0.54085	*2.32*
O	O123	0.64755	0.04684	0.71216	*1.17*
O	O124	0.5878	0.94672	0.62217	*2.19*
O	O125	0.70977	0.12318	0.84399	*1.55*
O	O126	0.00737	0.8625	0.78998	*1.28*
O	O127	0.89576	0.84891	0.69036	*1.75*
O	O128	0.8175	0.83391	0.53254	*2.71*
O	O129	0.7652	0.86749	0.70884	*2.68*
O	O130	0.69309	0.84263	0.46798	*2.16*
O	O131	0.57816	0.82395	0.56085	*2.71*
O	O132	0.63656	0.84923	0.73537	*1.96*
Si	Si61	0.93492	0.05756	0.81	*1.23*
Si	Si62	0.83666	0.03034	0.64458	*1.79*
Si	Si63	0.78624	0.07374	0.44292	*2.56*
Si	Si64	0.63087	0.07347	0.44288	*2.17*
Si	Si65	0.58367	0.02588	0.64556	*2.06*
Si	Si66	0.70985	0.05116	0.78882	*1.04*
Si	Si67	0.93127	0.83493	0.79706	*1.93*
Si	Si68	0.82878	0.87522	0.63489	*2.79*
Si	Si69	0.76992	0.826	0.43509	*2.32*
Si	Si70	0.61394	0.82706	0.45189	*2.28*
Si	Si71	0.57767	0.8705	0.65988	*2.15*
Si	Si72	0.70979	0.83186	0.77867	*2.13*
O	O133	0.49877	0.47182	0.70785	*2.17*
O	O134	0.38796	0.43811	0.80116	*1.46*
O	O135	0.4165	0.3843	0.62538	*1.65*
O	O136	0.38052	0.51233	0.64176	*2.17*

O	O137	0.31646	0.43249	0.96297	*2.08*
O	O138	0.33916	0.54945	0.88092	*2.59*
O	O139	0.25818	0.46407	0.78876	*2.54*
O	O140	0.28066	0.36107	0.11758	*1.75*
O	O141	0.19424	0.44357	0.02722	*2.06*
O	O142	0.29299	0.49235	0.1375	*1.93*
O	O143	0.10337	0.37248	0.12177	*1.66*
O	O144	0.07141	0.44023	0.95785	*2.51*
O	O145	0.12785	0.44766	0.77843	*2.56*
O	O146	0.08899	0.55745	0.86843	*2.54*
O	O147	0.20485	0.37697	0.66177	*2.39*
O	O148	0.49901	0.64517	0.70568	*1.71*
O	O149	0.38709	0.66396	0.81434	*2.04*
O	O150	0.31006	0.66158	0.9763	*2.33*
O	O151	0.2566	0.63453	0.80002	*2.49*
O	O152	0.18678	0.65989	0.04813	*2.32*
O	O153	0.06537	0.66686	0.97151	*2.58*
O	O154	0.12908	0.67328	0.79781	*2.47*
Si	Si73	0.42134	0.45108	0.69256	*0.96*
Si	Si74	0.32428	0.47139	0.85756	*2.22*
Si	Si75	0.27097	0.43261	0.06276	*1.97*
Si	Si76	0.1163	0.44011	0.05875	*1.57*
Si	Si77	0.07198	0.47922	0.85113	*2.48*
Si	Si78	0.19621	0.44948	0.71436	*2.16*
Si	Si79	0.42431	0.67733	0.70807	*2.35*
Si	Si80	0.3234	0.62828	0.86806	*2.76*
Si	Si81	0.26529	0.67337	0.07574	*0.29*
Si	Si82	0.10894	0.67795	0.07259	*2.01*
Si	Si83	0.07034	0.63592	0.8599	*2.02*
Si	Si84	0.19993	0.67521	0.73907	*2.30*
O	O155	0.98225	0.54349	0.20159	*0.64*
O	O156	0.08892	0.54768	0.31195	*1.85*
O	O157	0.07881	0.63248	0.16151	*2.58*
O	O158	0.09859	0.50514	0.12501	*1.39*
O	O159	0.16756	0.5767	0.46328	*2.11*
O	O160	0.16039	0.45364	0.4019	*2.92*
O	O161	0.2183	0.54352	0.28345	*2.19*
O	O162	0.19321	0.64073	0.6298	*2.44*
O	O163	0.28773	0.57047	0.53385	*2.32*
O	O164	0.19067	0.50761	0.63052	*2.68*
O	O165	0.37988	0.64449	0.61917	*1.98*
O	O166	0.41173	0.56693	0.46746	*0.76*
O	O167	0.34913	0.54608	0.29562	*1.40*

O	O168	0.40879	0.44674	0.38744	*2.14*
O	O169	0.28689	0.62331	0.16499	*2.53*
**O**	O170	0.98856	0.36218	0.21964	***3.48***
O	O171	0.10041	0.3493	0.31887	*2.35*
O	O172	0.17913	0.33398	0.47606	*2.53*
O	O173	0.2309	0.36764	0.2994	*2.74*
O	O174	0.30348	0.34273	0.54091	*2.06*
O	O175	0.41857	0.32398	0.44862	*2.42*
O	O176	0.35965	0.34923	0.27446	*2.15*
Si	Si85	0.06173	0.55755	0.19894	*1.70*
Si	Si86	0.15977	0.5306	0.36461	*2.55*
Si	Si87	0.20975	0.57368	0.56653	*2.18*
Si	Si88	0.36531	0.5735	0.56599	*2.36*
Si	Si89	0.41275	0.52582	0.36322	*2.53*
Si	Si90	0.28641	0.55121	0.21987	*1.65*
Si	Si91	0.06477	0.33493	0.21241	*2.20*
Si	Si92	0.16761	0.37543	0.3739	*2.81*
Si	Si93	0.22662	0.32612	0.57361	*2.05*
Si	Si94	0.3826	0.32713	0.55744	*2.06*
Si	Si95	0.41873	0.37053	0.34961	*2.06*
Si	Si96	0.28665	0.33194	0.23025	*1.85*
O	O177	0.43001	0.75631	0.68737	*2.39*
O	O178	0.27514	0.74989	0.11429	*1.43*
O	O179	0.10279	0.75511	0.10937	*0.26*
O	O180	0.2231	0.75226	0.72734	*2.01*
O	O181	0.06635	0.25628	0.18801	*1.72*
O	O182	0.22131	0.24973	0.61407	*1.60*
O	O183	0.39373	0.25498	0.61001	*1.43*
O	O184	0.27346	0.25227	0.22719	*2.48*
O	O185	0.56629	0.25624	0.32106	*2.57*
O	O186	0.72136	0.24978	0.89498	*1.43*
O	O187	0.89362	0.25507	0.89931	*0.26*
O	O188	0.77314	0.25209	0.28172	*2.46*
O	O189	0.92997	0.7563	0.82181	*2.13*
O	O190	0.77518	0.74962	0.39461	*1.60*
O	O191	0.60274	0.7549	0.39939	*1.18*
O	O192	0.72294	0.7522	0.78161	*2.36*

Supporting Information 6. Average Root Mean Square Deviation values (in Å) of each studied structure with different type and number of adsorbed coke precursors.

The bolded data in the tables indicate the minimum and maximum average RMSD used in normalization of RMSD (*NRMSD*, Equation on Page 6 of the main text).

Table S7. Average RMSD for structures with BAS between T12-T12.

Type of molecule	Number of molecules	Av. RMSD
**BENZENE**	1	0.60
	2	0.86
	3	1.11
	4	0.97
**TOLUENE**	1	0.83
	2	0.89
	3	1.11
	4	1.18
**NAPHTHALENE**	1	1.59
	2	1.75
	3	1.82
	**4**	**2.06**
**1,2,3,4-TMB**	1	1.74
	2	1.97
	3	1.96
	4	1.89
**1,2,4,5-TMB**	1	1.76
	2	1.87
	3	2.01
	4	1.95
**HMB**	1	1.88
	2	1.93
	3	1.91
	4	1.89

Table S8. Average RMSD for structures with BAS between T12-T11.

Type of molecule	Number of molecules	Av. RMSD
**BENZENE**	1	1.81
	2	1.81
	3	1.83
	4	1.79
**TOLUENE**	1	0.96
	2	1.88
	3	1.80
	4	1.77
**NAPHTHALENE**	1	1.32
	2	1.87
	3	1.86
	4	1.87
**1,2,3,4-TMB**	1	1.36
	2	1.84
	3	1.83
	4	1.82
**1,2,4,5-TMB**	1	1.83
	2	1.81
	3	1.81
	4	1.81
**HMB**	1	1.65
	2	1.87
	3	1.95
	4	1.95

Table S9. Average RMSD for structures with BAS between T7-T8.

Type of molecule	Number of molecules	Av. RMSD
**BENZENE**	1	1.21
	2	1.50
	3	1.78
	4	1.67
**TOLUENE**	1	1.14
	2	1.40
	3	1.67
	4	1.67
**NAPHTHALENE**	1	1.35
	2	1.53
	3	1.68
	4	1.76
**1,2,3,4-TMB**	1	1.18
	2	1.76
	3	1.95
	4	1.80
**1,2,4,5-TMB**	1	1.19
	2	1.72
	3	1.64
	4	1.81
**HMB**	1	1.38
	2	1.87
	3	1.83
	4	1.84

Table S10. Average RMSD for structures with BAS between T8-T7.

Type of molecule	Number of molecules	Av. RMSD
**BENZENE**	1	1.40
	2	1.80
	3	1.92
	4	1.82
**TOLUENE**	1	1.62
	2	1.80
	3	1.72
	4	1.73
**NAPHTHALENE**	1	1.72
	2	1.78
	3	1.93
	4	1.75
**1,2,3,4-TMB**	1	1.58
	2	1.86
	3	1.92
	4	1.88
**1,2,4,5-TMB**	1	1.54
	2	1.90
	3	1.79
	4	1.83
**HMB**	1	1.64
	2	1.82
	3	1.92
	4	1.91

Table S11. Average RMSD for structures without BAS (silicalite).

Type of molecule	Number of molecules	Av. RMSD
**BENZENE**	**1**	**0.03**
	2	0.49
	3	0.81
	4	0.99
**TOLUENE**	1	0.90
	2	0.80
	3	0.46
	4	0.48
**NAPHTHALENE**	1	0.71
	2	0.87
	3	0.78
	4	0.90
**1,2,3,4-TMB**	1	1.09
	2	1.86
	3	1.91
	4	1.88
**1,2,4,5-TMB**	1	1.30
	2	1.25
	3	1.14
	4	1.41
**HMB**	1	1.81
	2	2.01
	3	1.92
	4	2.04

Supporting Information 7. Relationship between local (D_{max}/D_{min}) and global (a-b) flexibility descriptors of models sharing the same BAS location.

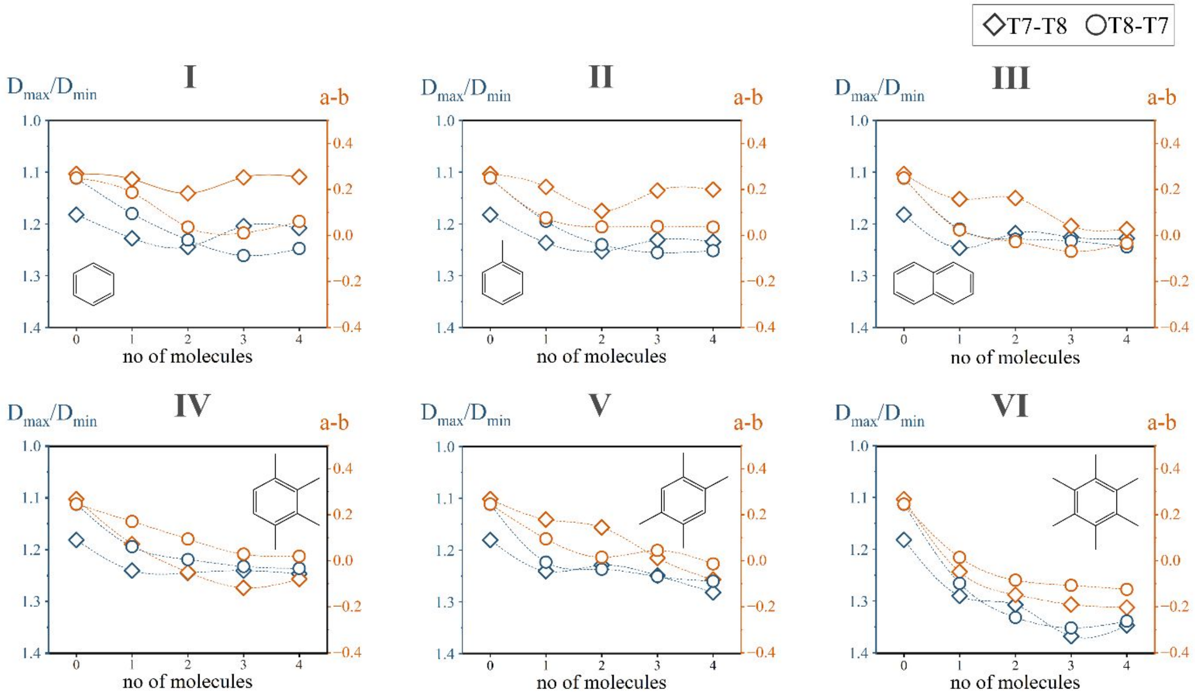

Figure S6. Relationship between local (Dmax/Dmin) and global (a-b) flexibility descriptors of models sharing the same BAS location but with different Al position, dependent on the number and type of guest molecule (roman numeral).

Supporting Information 8. Relative changes of unit cell parameter vectors

Information on the relative change of each unit cell parameter, for zeolite loaded with 4 molecules/u.c., calculated using Equation 1.

$$\Delta u=((u_{loaded}-u_{empty})/u_{empty})\cdot 100\% \qquad \text{Eq. 1}$$

where u – unit cell vector

The positive value means that this parameter became larger after loading with 4 molecules/u.c., whereas negative value indicates the decrease of this unit cell parameter.

Additionally, the shortest carbon-carbon distance between two neighboring molecules of the same pore is reported, measured as depicted in Figure S7.

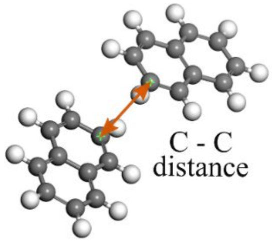

Figure S7. Representation on how the carbon-carbon distance was measured.

Supporting Information 9. Hirshfeld charges

I. T12-T11 model with four benzene molecules

#Atom	Element	*Net charge*
1	O	*-0.399*
2	O	*-0.403*
3	O	*-0.4*
4	O	*-0.416*
5	O	*-0.411*
6	O	*-0.409*
7	O	*-0.412*
8	O	*-0.445*
9	O	*-0.413*
10	O	*-0.412*
11	O	*-0.398*
12	O	*-0.4*
13	O	*-0.396*
14	O	*-0.41*
15	O	*-0.394*
16	O	*-0.405*
17	O	*-0.404*
18	O	*-0.407*
19	O	*-0.418*
20	O	*-0.413*
21	O	*-0.41*
22	O	*-0.412*
23	Si	*0.795*
24	Si	*0.785*
25	Si	*0.749*
26	Si	*0.796*
27	Si	*0.801*
28	Si	*0.798*
29	Si	*0.794*
30	Si	*0.779*
31	Si	*0.787*
32	Si	*0.802*
33	Si	*0.789*
34	Si	*0.765*
35	O	*-0.395*
36	O	*-0.4*
37	O	*-0.399*
38	O	*-0.416*
39	O	*-0.396*
40	O	*-0.413*
41	O	*-0.41*
42	O	*-0.403*
43	O	*-0.411*
44	O	*-0.409*
45	O	*-0.397*
46	O	*-0.397*
47	O	*-0.395*
48	O	*-0.403*
49	O	*-0.391*
50	O	*-0.415*
51	O	*-0.416*
52	O	*-0.419*
53	O	*-0.456*
54	O	*-0.412*
55	O	*-0.4*
56	O	*-0.551*
57	Si	*0.799*
58	Si	*0.795*
59	Si	*0.786*
60	Si	*0.798*
61	Si	*0.8*
62	Si	*0.791*
63	Si	*0.789*
64	Si	*0.752*
65	Si	*0.791*
66	Si	*0.807*
67	Si	*0.784*
68	Al	*0.627*
69	O	*-0.397*
70	O	*-0.4*
71	O	*-0.397*
72	O	*-0.414*
73	O	*-0.397*
74	O	*-0.407*
75	O	*-0.411*
76	O	*-0.394*
77	O	*-0.411*
78	O	*-0.406*
79	O	*-0.397*
80	O	*-0.399*
81	O	*-0.396*
82	O	*-0.409*
83	O	*-0.392*
84	O	*-0.408*
85	O	*-0.405*
86	O	*-0.405*
87	O	*-0.419*
88	O	*-0.414*
89	O	*-0.41*
90	O	*-0.413*
91	Si	*0.791*
92	Si	*0.795*
93	Si	*0.792*
94	Si	*0.797*
95	Si	*0.801*
96	Si	*0.795*
97	Si	*0.788*
98	Si	*0.786*
99	Si	*0.792*
100	Si	*0.802*
101	Si	*0.788*
102	Si	*0.766*
103	O	*-0.396*
104	O	*-0.399*
105	O	*-0.397*

106	O	*-0.414*
107	O	*-0.395*
108	O	*-0.408*
109	O	*-0.409*
110	O	*-0.403*
111	O	*-0.41*
112	O	*-0.409*
113	O	*-0.398*
114	O	*-0.399*
115	O	*-0.393*
116	O	*-0.41*
117	O	*-0.39*
118	O	*-0.416*
119	O	*-0.41*
120	O	*-0.414*
121	O	*-0.413*
122	O	*-0.415*
123	O	*-0.408*
124	O	*-0.402*
125	Si	*0.799*
126	Si	*0.795*
127	Si	*0.785*
128	Si	*0.797*
129	Si	*0.803*
130	Si	*0.801*
131	Si	*0.789*
132	Si	*0.765*
133	Si	*0.788*
134	Si	*0.8*
135	Si	*0.797*
136	Si	*0.798*
137	O	*-0.401*
138	O	*-0.401*
139	O	*-0.397*
140	O	*-0.415*
141	O	*-0.396*
142	O	*-0.412*
143	O	*-0.41*
144	O	*-0.394*
145	O	*-0.411*
146	O	*-0.408*
147	O	*-0.398*
148	O	*-0.398*
149	O	*-0.394*
150	O	*-0.404*
151	O	*-0.392*
152	O	*-0.416*
153	O	*-0.412*
154	O	*-0.418*
155	O	*-0.457*
156	O	*-0.411*
157	O	*-0.4*
158	O	*-0.551*
159	Si	*0.788*
160	Si	*0.788*
161	Si	*0.792*
162	Si	*0.798*
163	Si	*0.805*
164	Si	*0.799*
165	Si	*0.787*
166	Si	*0.751*
167	Si	*0.791*
168	Si	*0.807*
169	Si	*0.786*
170	Al	*0.628*
171	O	*-0.4*
172	O	*-0.403*
173	O	*-0.399*
174	O	*-0.415*
175	O	*-0.406*
176	O	*-0.407*
177	O	*-0.412*
178	O	*-0.447*
179	O	*-0.413*
180	O	*-0.412*
181	O	*-0.398*
182	O	*-0.4*
183	O	*-0.396*
184	O	*-0.41*
185	O	*-0.392*
186	O	*-0.407*
187	O	*-0.409*
188	O	*-0.405*
189	O	*-0.415*
190	O	*-0.414*
191	O	*-0.409*
192	O	*-0.4*
193	Si	*0.795*
194	Si	*0.785*
195	Si	*0.749*
196	Si	*0.795*
197	Si	*0.8*
198	Si	*0.797*
199	Si	*0.79*
200	Si	*0.784*
201	Si	*0.791*
202	Si	*0.798*
203	Si	*0.798*
204	Si	*0.799*
205	O	*-0.394*
206	O	*-0.401*
207	O	*-0.398*
208	O	*-0.414*
209	O	*-0.396*
210	O	*-0.409*
211	O	*-0.41*
212	O	*-0.402*
213	O	*-0.41*
214	O	*-0.407*
215	O	*-0.398*
216	O	*-0.397*
217	O	*-0.395*

218	O	*-0.403*
219	O	*-0.392*
220	O	*-0.403*
221	O	*-0.41*
222	O	*-0.409*
223	O	*-0.458*
224	O	*-0.412*
225	O	*-0.4*
226	O	*-0.55*
227	Si	*0.799*
228	Si	*0.796*
229	Si	*0.787*
230	Si	*0.798*
231	Si	*0.805*
232	Si	*0.798*
233	Si	*0.794*
234	Si	*0.769*
235	Si	*0.791*
236	Si	*0.807*
237	Si	*0.786*
238	Al	*0.627*
239	O	*-0.397*
240	O	*-0.402*
241	O	*-0.401*
242	O	*-0.416*
243	O	*-0.409*
244	O	*-0.409*
245	O	*-0.412*
246	O	*-0.447*
247	O	*-0.413*
248	O	*-0.413*
249	O	*-0.398*
250	O	*-0.4*
251	O	*-0.392*
252	O	*-0.41*
253	O	*-0.392*
254	O	*-0.407*
255	O	*-0.406*
256	O	*-0.405*
257	O	*-0.417*
258	O	*-0.412*
259	O	*-0.41*
260	O	*-0.412*
261	Si	*0.796*
262	Si	*0.786*
263	Si	*0.747*
264	Si	*0.795*
265	Si	*0.803*
266	Si	*0.802*
267	Si	*0.79*
268	Si	*0.786*
269	Si	*0.791*
270	Si	*0.802*
271	Si	*0.788*
272	Si	*0.765*
273	O	*-0.399*
274	O	*-0.392*
275	O	*-0.395*
276	O	*-0.435*
277	O	*-0.4*
278	O	*-0.394*
279	O	*-0.395*
280	O	*-0.436*
281	O	*-0.397*
282	O	*-0.394*
283	O	*-0.395*
284	O	*-0.435*
285	O	*-0.398*
286	O	*-0.394*
287	O	*-0.396*
288	O	*-0.392*
289	H	*0.594*
290	H	*0.594*
291	H	*0.594*
292	C	*-0.434*
293	H	*0.453*
294	C	*-0.368*
295	H	*0.454*
296	C	*-0.396*
297	H	*0.453*
298	H	*0.455*
299	C	*-0.386*
300	H	*0.456*
301	C	*-0.419*
302	H	*0.454*
303	C	*-0.386*
304	C	*-0.414*
305	H	*0.454*
306	C	*-0.386*
307	H	*0.455*
308	H	*0.454*
309	H	*0.453*
310	C	*-0.37*
311	H	*0.455*
312	C	*-0.434*
313	H	*0.454*
314	C	*-0.389*
315	C	*-0.397*
316	C	*-0.375*
317	H	*0.451*
318	C	*-0.396*
319	H	*0.449*
320	C	*-0.398*
321	H	*0.45*
322	H	*0.45*
323	C	*-0.424*
324	H	*0.45*
325	C	*-0.404*
326	H	*0.451*
327	C	*-0.411*
328	H	*0.454*
329	C	*-0.373*

330	H	*0.454*	336	C	*-0.384*
331	C	*-0.435*	337	H	*0.455*
332	H	*0.454*	338	C	*-0.388*
333	C	*-0.397*	339	H	*0.455*
334	C	*-0.413*			
335	H	*0.454*			

II. T12-T11 with four hexamethylbenzene molecules

#Atom	Element	*Net charge*			
1	O	*-0.407*	35	O	*-0.406*
2	O	*-0.438*	36	O	*-0.409*
3	O	*-0.407*	37	O	*-0.409*
4	O	*-0.414*	38	O	*-0.416*
5	O	*-0.44*	39	O	*-0.422*
6	O	*-0.409*	40	O	*-0.41*
7	O	*-0.408*	41	O	*-0.405*
8	O	*-0.448*	42	O	*-0.414*
9	O	*-0.415*	43	O	*-0.412*
10	O	*-0.435*	44	O	*-0.419*
11	O	*-0.397*	45	O	*-0.399*
12	O	*-0.401*	46	O	*-0.4*
13	O	*-0.43*	47	O	*-0.43*
14	O	*-0.408*	48	O	*-0.402*
15	O	*-0.396*	49	O	*-0.395*
16	O	*-0.429*	50	O	*-0.437*
17	O	*-0.422*	51	O	*-0.427*
18	O	*-0.427*	52	O	*-0.445*
19	O	*-0.416*	53	O	*-0.455*
20	O	*-0.413*	54	O	*-0.411*
21	O	*-0.41*	55	O	*-0.401*
22	O	*-0.421*	56	O	*-0.558*
23	Si	*0.764*	57	Si	*0.759*
24	Si	*0.726*	58	Si	*0.771*
25	Si	*0.724*	59	Si	*0.754*
26	Si	*0.797*	60	Si	*0.801*
27	Si	*0.784*	61	Si	*0.774*
28	Si	*0.744*	62	Si	*0.734*
29	Si	*0.737*	63	Si	*0.734*
30	Si	*0.733*	64	Si	*0.721*
31	Si	*0.776*	65	Si	*0.771*
32	Si	*0.774*	66	Si	*0.768*
33	Si	*0.765*	67	Si	*0.76*
34	Si	*0.763*	68	Al	*0.621*
			69	O	*-0.404*

70	O	*-0.409*	113	O	*-0.4*
71	O	*-0.413*	114	O	*-0.402*
72	O	*-0.414*	115	O	*-0.416*
73	O	*-0.414*	116	O	*-0.409*
74	O	*-0.408*	117	O	*-0.397*
75	O	*-0.407*	118	O	*-0.435*
76	O	*-0.398*	119	O	*-0.425*
77	O	*-0.413*	120	O	*-0.437*
78	O	*-0.405*	121	O	*-0.412*
79	O	*-0.398*	122	O	*-0.413*
80	O	*-0.401*	123	O	*-0.408*
81	O	*-0.429*	124	O	*-0.417*
82	O	*-0.408*	125	Si	*0.755*
83	O	*-0.397*	126	Si	*0.766*
84	O	*-0.424*	127	Si	*0.754*
85	O	*-0.412*	128	Si	*0.799*
86	O	*-0.418*	129	Si	*0.789*
87	O	*-0.417*	130	Si	*0.766*
88	O	*-0.413*	131	Si	*0.732*
89	O	*-0.41*	132	Si	*0.732*
90	O	*-0.421*	133	Si	*0.771*
91	Si	*0.748*	134	Si	*0.775*
92	Si	*0.772*	135	Si	*0.774*
93	Si	*0.775*	136	Si	*0.786*
94	Si	*0.799*	137	O	*-0.406*
95	Si	*0.784*	138	O	*-0.448*
96	Si	*0.744*	139	O	*-0.401*
97	Si	*0.747*	140	O	*-0.415*
98	Si	*0.759*	141	O	*-0.422*
99	Si	*0.78*	142	O	*-0.409*
100	Si	*0.773*	143	O	*-0.404*
101	Si	*0.767*	144	O	*-0.404*
102	Si	*0.764*	145	O	*-0.413*
103	O	*-0.402*	146	O	*-0.428*
104	O	*-0.408*	147	O	*-0.399*
105	O	*-0.409*	148	O	*-0.401*
106	O	*-0.414*	149	O	*-0.42*
107	O	*-0.414*	150	O	*-0.402*
108	O	*-0.405*	151	O	*-0.395*
109	O	*-0.403*	152	O	*-0.435*
110	O	*-0.419*	153	O	*-0.426*
111	O	*-0.412*	154	O	*-0.446*
112	O	*-0.415*	155	O	*-0.455*

156	O	*-0.411*	199	Si	*0.752*
157	O	*-0.401*	200	Si	*0.758*
158	O	*-0.558*	201	Si	*0.78*
159	Si	*0.756*	202	Si	*0.774*
160	Si	*0.729*	203	Si	*0.753*
161	Si	*0.75*	204	Si	*0.791*
162	Si	*0.801*	205	O	*-0.404*
163	Si	*0.78*	206	O	*-0.407*
164	Si	*0.761*	207	O	*-0.407*
165	Si	*0.737*	208	O	*-0.415*
166	Si	*0.717*	209	O	*-0.424*
167	Si	*0.775*	210	O	*-0.407*
168	Si	*0.771*	211	O	*-0.405*
169	Si	*0.763*	212	O	*-0.413*
170	Al	*0.621*	213	O	*-0.412*
171	O	*-0.407*	214	O	*-0.41*
172	O	*-0.438*	215	O	*-0.399*
173	O	*-0.405*	216	O	*-0.4*
174	O	*-0.414*	217	O	*-0.422*
175	O	*-0.436*	218	O	*-0.402*
176	O	*-0.406*	219	O	*-0.395*
177	O	*-0.408*	220	O	*-0.421*
178	O	*-0.448*	221	O	*-0.418*
179	O	*-0.415*	222	O	*-0.425*
180	O	*-0.428*	223	O	*-0.457*
181	O	*-0.398*	224	O	*-0.412*
182	O	*-0.401*	225	O	*-0.402*
183	O	*-0.431*	226	O	*-0.558*
184	O	*-0.41*	227	Si	*0.763*
185	O	*-0.396*	228	Si	*0.774*
186	O	*-0.422*	229	Si	*0.757*
187	O	*-0.415*	230	Si	*0.801*
188	O	*-0.418*	231	Si	*0.78*
189	O	*-0.412*	232	Si	*0.763*
190	O	*-0.413*	233	Si	*0.759*
191	O	*-0.412*	234	Si	*0.743*
192	O	*-0.418*	235	Si	*0.774*
193	Si	*0.766*	236	Si	*0.77*
194	Si	*0.728*	237	Si	*0.764*
195	Si	*0.729*	238	Al	*0.623*
196	Si	*0.798*	239	O	*-0.405*
197	Si	*0.773*	240	O	*-0.439*
198	Si	*0.733*	241	O	*-0.406*

242	O	*-0.415*	285	O	*-0.402*
243	O	*-0.439*	286	O	*-0.395*
244	O	*-0.408*	287	O	*-0.42*
245	O	*-0.409*	288	O	*-0.391*
246	O	*-0.447*	289	H	*0.595*
247	O	*-0.415*	290	H	*0.595*
248	O	*-0.429*	291	H	*0.596*
249	O	*-0.399*	292	C	*-0.034*
250	O	*-0.401*	293	C	*-0.013*
251	O	*-0.418*	294	C	*-0.009*
252	O	*-0.409*	295	C	*-1.155*
253	O	*-0.393*	296	H	*0.446*
254	O	*-0.42*	297	H	*0.441*
255	O	*-0.415*	298	H	*0.451*
256	O	*-0.417*	299	C	*-1.059*
257	O	*-0.416*	300	H	*0.447*
258	O	*-0.412*	301	H	*0.45*
259	O	*-0.411*	302	H	*0.447*
260	O	*-0.421*	303	C	*-0.099*
261	Si	*0.764*	304	C	*-0.014*
262	Si	*0.726*	305	C	*-0.012*
263	Si	*0.729*	306	C	*-1.101*
264	Si	*0.796*	307	H	*0.446*
265	Si	*0.785*	308	H	*0.451*
266	Si	*0.785*	309	H	*0.444*
267	Si	*0.752*	310	C	*-1.122*
268	Si	*0.757*	311	H	*0.451*
269	Si	*0.783*	312	H	*0.444*
270	Si	*0.776*	313	H	*0.448*
271	Si	*0.764*	314	C	*-1.004*
272	Si	*0.763*	315	H	*0.453*
273	O	*-0.401*	316	H	*0.442*
274	O	*-0.394*	317	H	*0.445*
275	O	*-0.413*	318	C	*-1.08*
276	O	*-0.436*	319	H	*0.441*
277	O	*-0.403*	320	H	*0.452*
278	O	*-0.395*	321	H	*0.447*
279	O	*-0.411*	322	C	*-0.016*
280	O	*-0.436*	323	C	*-0.018*
281	O	*-0.402*	324	C	*-0.02*
282	O	*-0.395*	325	C	*-1.065*
283	O	*-0.414*	326	H	*0.445*
284	O	*-0.435*	327	H	*0.448*

328	H	*0.441*
329	C	*-1.095*
330	H	*0.44*
331	H	*0.444*
332	H	*0.446*
333	C	*-0.025*
334	C	*-0.02*
335	C	*-0.028*
336	C	*-1.006*
337	H	*0.44*
338	H	*0.448*
339	H	*0.444*
340	C	*-1.167*
341	H	*0.445*
342	H	*0.445*
343	H	*0.441*
344	C	*-1.064*
345	H	*0.444*
346	H	*0.444*
347	H	*0.447*
348	C	*-1.128*
349	H	*0.444*
350	H	*0.446*
351	H	*0.446*
352	C	*-0.098*
353	C	*-0.034*
354	C	*-0.013*
355	C	*-1.006*
356	H	*0.445*
357	H	*0.441*
358	H	*0.45*
359	C	*-1.123*
360	H	*0.445*
361	H	*0.451*
362	H	*0.448*
363	C	*-0.012*
364	C	*-0.009*
365	C	*-0.013*
366	C	*-1.161*
367	H	*0.446*
368	H	*0.451*
369	H	*0.44*
370	C	*-1.093*
371	H	*0.45*
372	H	*0.446*
373	H	*0.443*
374	C	*-1.088*
375	H	*0.452*
376	H	*0.441*
377	H	*0.446*
378	C	*-1.063*
379	H	*0.447*
380	H	*0.448*
381	H	*0.449*
382	C	*-0.012*
383	C	*-0.01*
384	C	*-0.014*
385	C	*-1.091*
386	H	*0.449*
387	H	*0.446*
388	H	*0.443*
389	C	*-1.081*
390	H	*0.441*
391	H	*0.448*
392	H	*0.452*
393	C	*-0.033*
394	C	*-0.01*
395	C	*-0.097*
396	C	*-1.127*
397	H	*0.449*
398	H	*0.448*
399	H	*0.445*
400	C	*-1.06*
401	H	*0.447*
402	H	*0.45*
403	H	*0.446*
404	C	*-1.164*
405	H	*0.44*
406	H	*0.446*
407	H	*0.451*
408	C	*-1.009*
409	H	*0.452*
410	H	*0.444*
411	H	*0.441*

III. Silicalite with four hexamethylbenzene molecules

#Atom	Element	*Net charge*			
1	O	*-0.42*	40	O	*-0.426*
2	O	*-0.425*	41	O	*-0.405*
3	O	*-0.396*	42	O	*-0.4*
4	O	*-0.414*	43	O	*-0.407*
5	O	*-0.409*	44	O	*-0.409*
6	O	*-0.407*	45	O	*-0.403*
7	O	*-0.406*	46	O	*-0.405*
8	O	*-0.422*	47	O	*-0.414*
9	O	*-0.405*	48	O	*-0.41*
10	O	*-0.406*	49	O	*-0.406*
11	O	*-0.402*	50	O	*-0.426*
12	O	*-0.404*	51	O	*-0.411*
13	O	*-0.437*	52	O	*-0.4*
14	O	*-0.41*	53	O	*-0.414*
15	O	*-0.41*	54	O	*-0.419*
16	O	*-0.415*	55	O	*-0.406*
17	O	*-0.408*	56	O	*-0.423*
18	O	*-0.396*	57	Si	*0.76*
19	O	*-0.414*	58	Si	*0.763*
20	O	*-0.418*	59	Si	*0.787*
21	O	*-0.409*	60	Si	*0.793*
22	O	*-0.413*	61	Si	*0.767*
23	Si	*0.779*	62	Si	*0.771*
24	Si	*0.765*	63	Si	*0.766*
25	Si	*0.773*	64	Si	*0.746*
26	Si	*0.793*	65	Si	*0.785*
27	Si	*0.724*	66	Si	*0.778*
28	Si	*0.729*	67	Si	*0.753*
29	Si	*0.771*	68	Si	*0.735*
30	Si	*0.782*	69	O	*-0.419*
31	Si	*0.779*	70	O	*-0.425*
32	Si	*0.776*	71	O	*-0.396*
33	Si	*0.775*	72	O	*-0.415*
34	Si	*0.762*	73	O	*-0.409*
35	O	*-0.414*	74	O	*-0.407*
36	O	*-0.411*	75	O	*-0.405*
37	O	*-0.402*	76	O	*-0.423*
38	O	*-0.415*	77	O	*-0.405*
39	O	*-0.41*	78	O	*-0.406*
			79	O	*-0.402*

80	O	*-0.404*	123	O	*-0.406*
81	O	*-0.438*	124	O	*-0.422*
82	O	*-0.41*	125	Si	*0.761*
83	O	*-0.41*	126	Si	*0.763*
84	O	*-0.417*	127	Si	*0.787*
85	O	*-0.409*	128	Si	*0.793*
86	O	*-0.396*	129	Si	*0.767*
87	O	*-0.414*	130	Si	*0.773*
88	O	*-0.419*	131	Si	*0.766*
89	O	*-0.409*	132	Si	*0.745*
90	O	*-0.413*	133	Si	*0.785*
91	Si	*0.779*	134	Si	*0.777*
92	Si	*0.765*	135	Si	*0.755*
93	Si	*0.773*	136	Si	*0.732*
94	Si	*0.793*	137	O	*-0.414*
95	Si	*0.724*	138	O	*-0.41*
96	Si	*0.729*	139	O	*-0.402*
97	Si	*0.773*	140	O	*-0.415*
98	Si	*0.782*	141	O	*-0.411*
99	Si	*0.78*	142	O	*-0.426*
100	Si	*0.777*	143	O	*-0.405*
101	Si	*0.774*	144	O	*-0.4*
102	Si	*0.762*	145	O	*-0.408*
103	O	*-0.414*	146	O	*-0.409*
104	O	*-0.412*	147	O	*-0.403*
105	O	*-0.402*	148	O	*-0.405*
106	O	*-0.415*	149	O	*-0.414*
107	O	*-0.409*	150	O	*-0.41*
108	O	*-0.426*	151	O	*-0.407*
109	O	*-0.405*	152	O	*-0.428*
110	O	*-0.401*	153	O	*-0.411*
111	O	*-0.407*	154	O	*-0.4*
112	O	*-0.409*	155	O	*-0.413*
113	O	*-0.403*	156	O	*-0.419*
114	O	*-0.405*	157	O	*-0.406*
115	O	*-0.413*	158	O	*-0.422*
116	O	*-0.41*	159	Si	*0.76*
117	O	*-0.406*	160	Si	*0.764*
118	O	*-0.426*	161	Si	*0.786*
119	O	*-0.411*	162	Si	*0.793*
120	O	*-0.4*	163	Si	*0.768*
121	O	*-0.414*	164	Si	*0.77*
122	O	*-0.419*	165	Si	*0.766*

166	Si	*0.746*	209	O	*-0.414*
167	Si	*0.784*	210	O	*-0.427*
168	Si	*0.778*	211	O	*-0.406*
169	Si	*0.754*	212	O	*-0.401*
170	Si	*0.731*	213	O	*-0.407*
171	O	*-0.419*	214	O	*-0.409*
172	O	*-0.425*	215	O	*-0.403*
173	O	*-0.396*	216	O	*-0.405*
174	O	*-0.414*	217	O	*-0.414*
175	O	*-0.41*	218	O	*-0.41*
176	O	*-0.407*	219	O	*-0.407*
177	O	*-0.406*	220	O	*-0.426*
178	O	*-0.425*	221	O	*-0.411*
179	O	*-0.405*	222	O	*-0.4*
180	O	*-0.406*	223	O	*-0.413*
181	O	*-0.402*	224	O	*-0.419*
182	O	*-0.404*	225	O	*-0.406*
183	O	*-0.437*	226	O	*-0.422*
184	O	*-0.41*	227	Si	*0.758*
185	O	*-0.41*	228	Si	*0.768*
186	O	*-0.415*	229	Si	*0.783*
187	O	*-0.407*	230	Si	*0.793*
188	O	*-0.396*	231	Si	*0.769*
189	O	*-0.414*	232	Si	*0.77*
190	O	*-0.418*	233	Si	*0.765*
191	O	*-0.409*	234	Si	*0.746*
192	O	*-0.413*	235	Si	*0.785*
193	Si	*0.779*	236	Si	*0.777*
194	Si	*0.764*	237	Si	*0.755*
195	Si	*0.773*	238	Si	*0.732*
196	Si	*0.793*	239	O	*-0.419*
197	Si	*0.724*	240	O	*-0.425*
198	Si	*0.728*	241	O	*-0.396*
199	Si	*0.771*	242	O	*-0.415*
200	Si	*0.782*	243	O	*-0.41*
201	Si	*0.779*	244	O	*-0.407*
202	Si	*0.776*	245	O	*-0.405*
203	Si	*0.775*	246	O	*-0.424*
204	Si	*0.762*	247	O	*-0.405*
205	O	***-0.415***	248	O	*-0.406*
206	O	*-0.407*	249	O	*-0.402*
207	O	*-0.403*	250	O	*-0.404*
208	O	*-0.415*	251	O	*-0.438*

252	O	*-0.41*
253	O	*-0.41*
254	O	*-0.416*
255	O	*-0.407*
256	O	*-0.396*
257	O	*-0.414*
258	O	*-0.418*
259	O	*-0.409*
260	O	*-0.413*
261	Si	*0.779*
262	Si	*0.764*
263	Si	*0.773*
264	Si	*0.793*
265	Si	*0.724*
266	Si	*0.729*
267	Si	*0.77*
268	Si	*0.782*
269	Si	*0.779*
270	Si	*0.776*
271	Si	*0.775*
272	Si	*0.761*
273	O	*-0.393*
274	O	*-0.407*
275	O	*-0.402*
276	O	*-0.401*
277	O	*-0.393*
278	O	*-0.408*
279	O	*-0.402*
280	O	*-0.401*
281	O	*-0.393*
282	O	*-0.408*
283	O	*-0.402*
284	O	*-0.401*
285	O	*-0.393*
286	O	*-0.407*
287	O	*-0.402*
288	O	*-0.401*
289	C	*-0.027*
290	C	*-0.023*
291	C	*-0.025*
292	C	*-1.189*
293	H	*0.44*
294	H	*0.443*
295	H	*0.451*
296	C	*-1.063*
297	H	*0.444*
298	H	*0.447*
299	H	*0.446*
300	C	*-0.022*
301	C	*-0.02*
302	C	*-0.016*
303	C	*-0.027*
304	C	*-0.023*
305	C	*-0.025*
306	C	*-1.189*
307	H	*0.44*
308	H	*0.45*
309	H	*0.443*
310	C	*-1.064*
311	H	*0.444*
312	H	*0.446*
313	H	*0.447*
314	C	*-0.021*
315	C	*-0.02*
316	C	*-0.016*
317	C	*-0.024*
318	C	*-0.023*
319	C	*-0.027*
320	C	*-1.187*
321	H	*0.449*
322	H	*0.444*
323	H	*0.439*
324	C	*-1.063*
325	H	*0.447*
326	H	*0.444*
327	H	*0.446*
328	C	*-0.02*
329	C	*-0.021*
330	C	*-0.016*
331	C	*-0.025*
332	C	*-0.023*
333	C	*-0.027*
334	C	*-1.19*
335	H	*0.443*
336	H	*0.45*
337	H	*0.44*

338	C	*-1.064*	381	C	*-1.093*
339	H	*0.446*	382	H	*0.444*
340	H	*0.444*	383	H	*0.44*
341	H	*0.447*	384	H	*0.446*
342	C	*-0.02*	385	C	*-1.045*
343	C	*-0.022*	386	H	*0.448*
344	C	*-0.015*	387	H	*0.442*
345	C	*-1.042*	388	H	*0.443*
346	H	*0.448*	389	C	*-1.074*
347	H	*0.443*	390	H	*0.445*
348	H	*0.442*	391	H	*0.444*
349	C	*-1.076*	392	H	*0.442*
350	H	*0.444*	393	C	*-1.093*
351	H	*0.445*	394	H	*0.446*
352	H	*0.442*	395	H	*0.44*
353	C	*-1.095*	396	H	*0.444*
354	H	*0.446*	397	C	*-1.045*
355	H	***0.438***	398	H	*0.448*
356	H	*0.444*	399	H	*0.443*
357	C	*-1.122*	400	H	*0.442*
358	H	*0.446*	401	C	*-1.074*
359	H	*0.444*	402	H	*0.444*
360	H	*0.444*	403	H	*0.445*
361	C	*-1.092*	404	H	*0.442*
362	H	*0.444*	405	C	*-1.121*
363	H	*0.44*	406	H	*0.446*
364	H	*0.446*	407	H	*0.445*
365	C	*-1.122*	408	H	*0.444*
366	H	*0.446*			
367	H	*0.444*			
368	H	*0.445*			
369	C	*-1.044*			
370	H	*0.443*			
371	H	*0.448*			
372	H	*0.442*			
373	C	*-1.074*			
374	H	*0.445*			
375	H	*0.444*			
376	H	*0.442*			
377	C	*-1.121*			
378	H	*0.446*			
379	H	*0.444*			
380	H	*0.445*			

Supporting Information 10. Volume changes of studied model upon adsorption of coke precursors

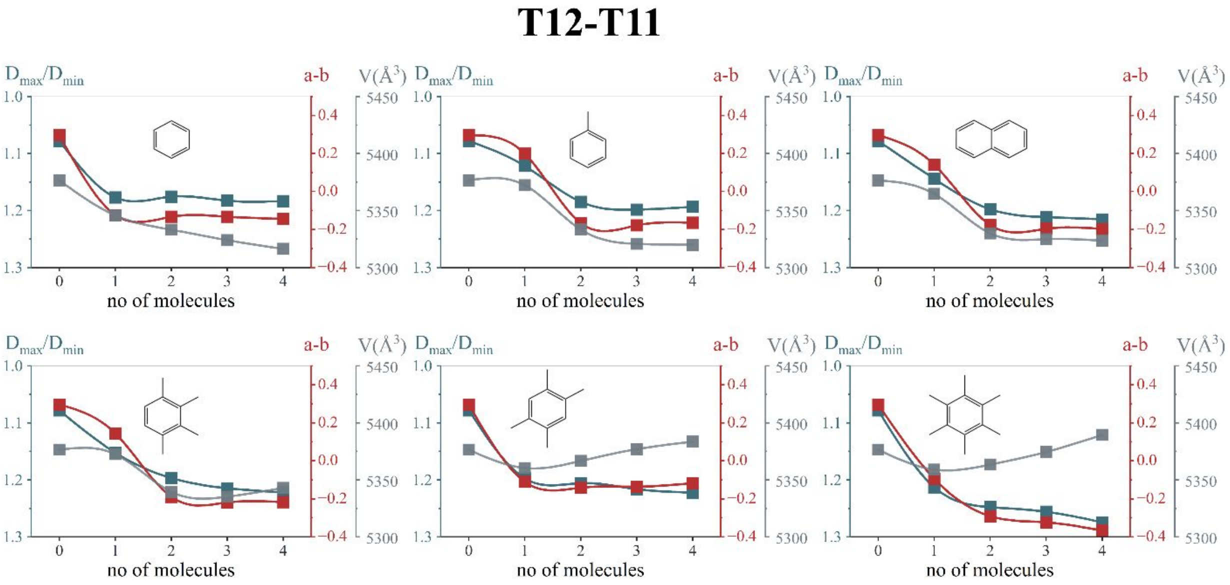

Figure S8. Relationship between number of molecules, (a-b), (Dmax/Dmin) and volume of the unit cell (V) for the most energetically favorable model of H-ZSM-5.

Supporting Information 11. Model with no active site (silicalite)

Results of structural changes for silicalite model with various aromatic molecules as coke precursors.

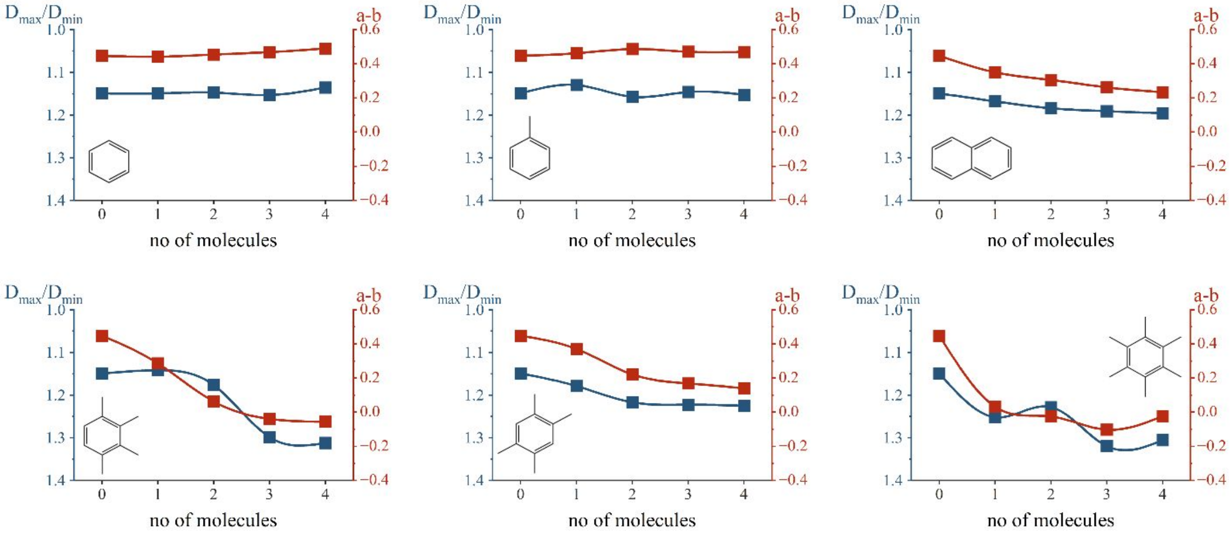

Figure S9. Relationship between local (D_{max}/D_{min}) and global (a-b) flexibility descriptor dependent on the number and type of guest molecule for H-ZSM-5 without active site (silicalite).

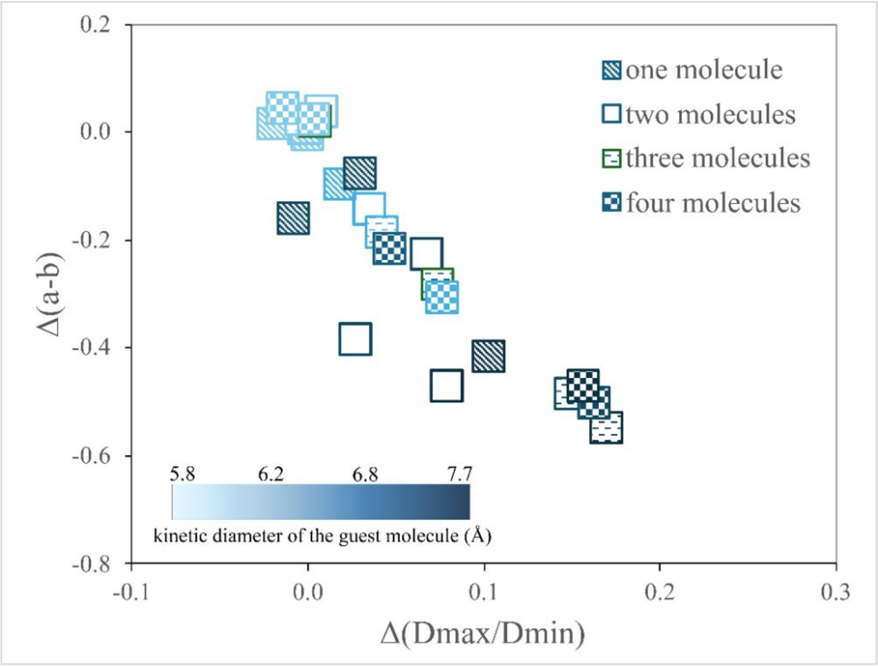

Figure S10. Scatter plot representing difference in local and global flexibility descriptors between silicalite loaded with various molecules and empty material.

Supporting Information 12. XRD data analysis

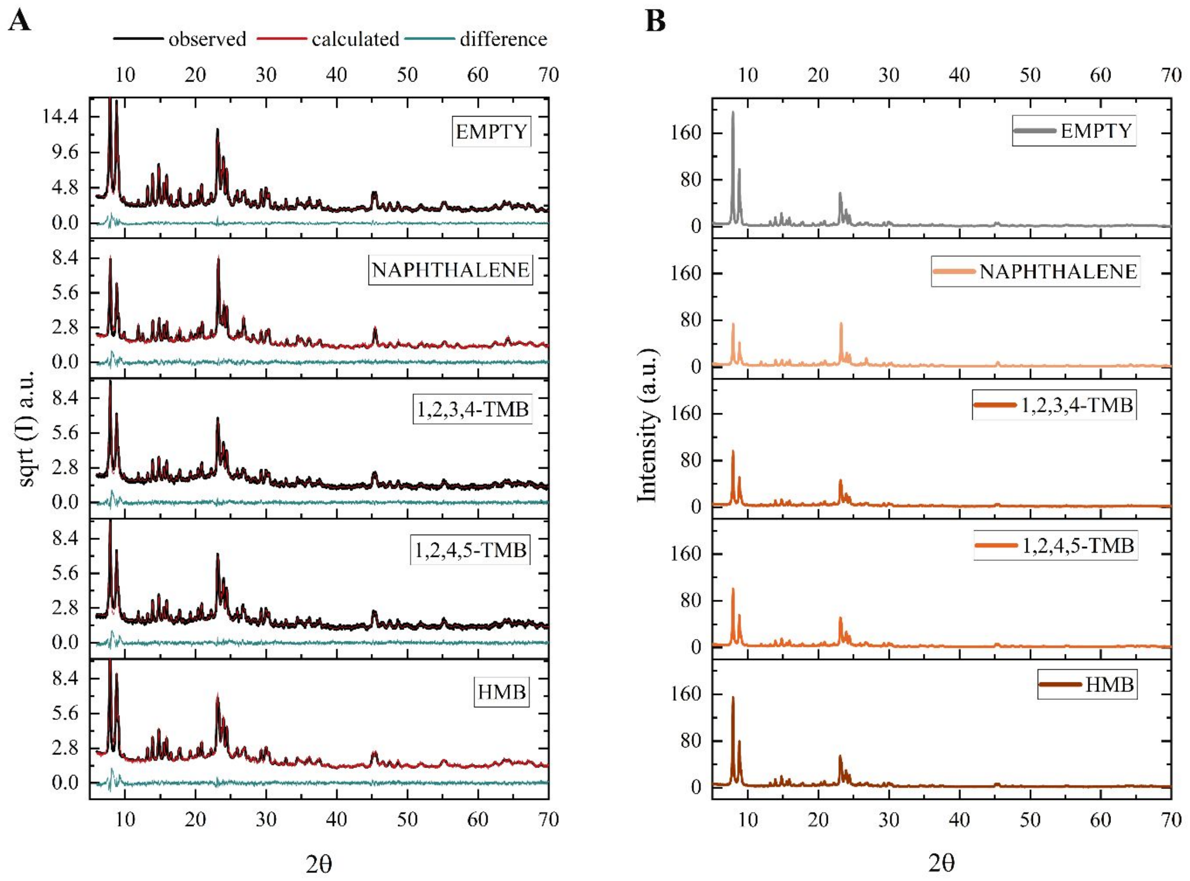

Figure S11. **A:** Quality of Rietveld fit, **B:** Full X-ray diffractograms.

Supporting Information 13. Atomic coordinates for dummy carbon atoms

Table S12. The dummy carbon atom positions identified via difference Fourier map analysis.

Site	Num_posns	x	y	z
**C1**	4	0.00000	0.00000	0.50000
**C2**	8	0.00000	0.29000	0.62000
**C3**	8	0.00000	0.29000	0.42000
**C4**	4	0.30000	0.25000	0.85000
**C5**	4	0.58000	0.25000	0.67000
**C6**	8	0.42145	0.17194	0.87513
**C7**	8	0.03960	0.77341	0.43790
